# Unveiling a family of spiro-β-lactams with anti-HIV and antiplasmodial activity *via* phosphine-catalyzed [3+2] annulation of 6-alkylidene-penicillanates and allenoates

**DOI:** 10.3389/fchem.2022.1017250

**Published:** 2022-10-07

**Authors:** Américo J. S. Alves, Nuno G. Alves, Inês Bártolo, Diana Fontinha, Soraia Caetano, Miguel Prudêncio, Nuno Taveira, Teresa M. V. D. Pinho e Melo

**Affiliations:** ^1^ Coimbra Chemistry Centre-Institute of Molecular Sciences and Department of Chemistry, University of Coimbra, Coimbra, Portugal; ^2^ Instituto de Investigação do Medicamento (iMed.ULisboa), Faculdade de Farmácia, Universidade de Lisboa, Lisboa, Portugal; ^3^ Instituto de Medicina Molecular João Lobo Antunes, Faculdade de Medicina, Universidade de Lisboa, Lisboa, Portugal; ^4^ Centro de Investigação Interdisciplinar Egas Moniz (CiiEM), Instituto Universitário Egas Moniz (IUEM), Caparica, Portugal

**Keywords:** spiro-penicillanates, β-Lactams, spirocyclic compounds, spiro-β-lactams, allenoates, anti-HIV, antiplasmodial activities, phosphane-catalyzed annulations

## Abstract

The molecular architecture of spirocyclic compounds has been widely explored within the medicinal chemistry field to obtain new compounds with singular three-dimensional pharmacophoric features and improved bioactivity. Herein, the synthesis of 68 new spirocyclopentene-β-lactams is described, resulting from a rational drug design and structural modulation of a highly promising lead compound BSS-730A, previously identified as having dual antimicrobial activity associated with a novel mechanism of action. Among this diverse library of new compounds, 22 were identified as active against HIV-1, with eight displaying an IC_50_ lower than 50 nM. These eight compounds also showed nanomolar activity against HIV-2, and six of them displayed micromolar antiplasmodial activity against both the hepatic and the blood stages of infection by malaria parasites, in agreement with the lead molecule’s bioactivity profile. The spirocyclopentene-β-lactams screened also showed low cytotoxicity against TZM-bl and Huh7 human cell lines. Overall, a family of new spirocyclopentene penicillanates with potent activity against HIV and/or *Plasmodium* was identified. The present structure–activity relationship open avenues for further development of spirocyclopentene-β-lactams as multivalent, highly active broad spectrum antimicrobial agents.

## Graphical Abstract

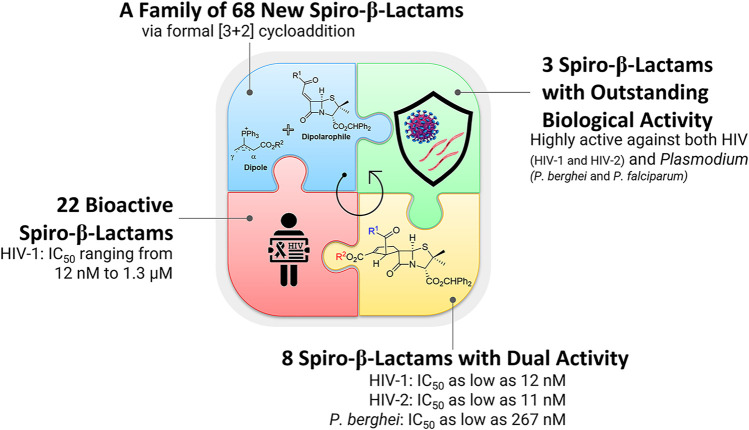

## 1 Introduction

**FIGURE 1:**
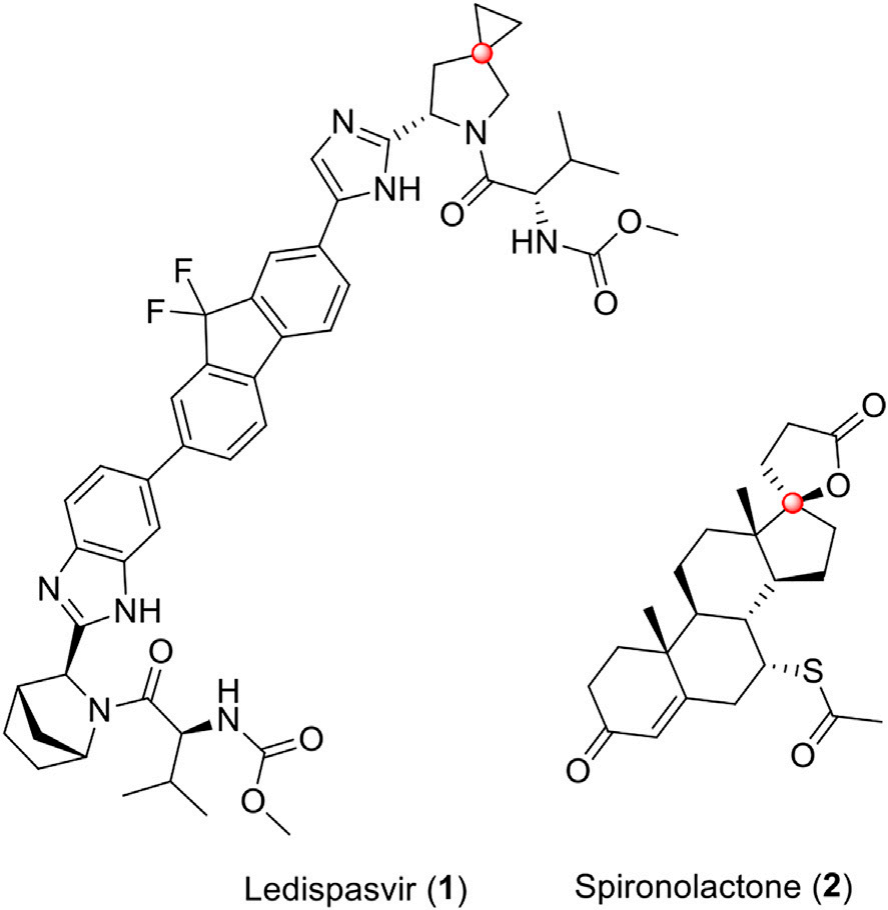
Ledipasvir (**1**) and spironolactone (**2**) are the two drugs in clinical use, which contain a spirocyclic scaffold in their molecular structures. Spiro carbons are highlighted in red.

Spirocyclic compounds are characterized by having two different rings that share the same *sp3* (Zheng et al., 2014) carbon, which allows a unique structural architecture (Ding et al., 2018; Xu et al., 2019). The conformational restraints and the rigidity of spirocyclic compounds mean that this class of molecules presents a reduced conformational entropy penalty, which favors putative target-ligand interactions (Zheng et al., 2014; Chupakhin et al., 2019). Thus, several spirocyclic compounds with promising and diverse bioactivities are known (Zhou et al., 2020; Alves et al., 2021a; Acosta-Quiroga et al., 2021; Boddy and Bull, 2021; Yang et al., 2022). Ledipasvir (**1**), (Link et al., 2014), an inhibitor of the hepatitis C virus (HCV) NS5A protein and spironolactone (**2**), (Gabbard et al., 2020), a potassium-sparing diuretic steroid, are examples of drugs in clinical use presenting a spirocyclic scaffold in its molecular structure (Figure 1).

Spiro-β-lactams represent one of the most versatile and promising subclasses of spirocyclic compounds. Several spiro-β-lactams have been shown to display pharmacological properties as varied as antimicrobial, anticancer, or inhibition of cholesterol absorption activities (Dhooghe and Thi, 2019; Alves et al., 2021b). Our research team has focused a lot of its efforts on the synthesis and investigation of the reactivity of new spirocyclic lactams derived from 6-aminopenicillanic acid (6-APA). By using 6-diazo- and 6-alkylidenepenicillanates in different [3+2] dipolar cycloadditions and [3+2] annulation reaction-based synthetic strategies, an extensive and diverse library of new spiropenicillanates containing pyrazole, cyclopentene, pyrazoline, cyclopropane, and isoxazolidine rings, spiro-fused to the penicillin core’s lactam ring was generated (Santos et al., 2012; Santos and Pinho e Melo, 2013; Gomes et al., 2014; Alves et al., 2020; Alves and Pinho e Melo, 2020; Alves et al., 2021c).

**FIGURE 2:**
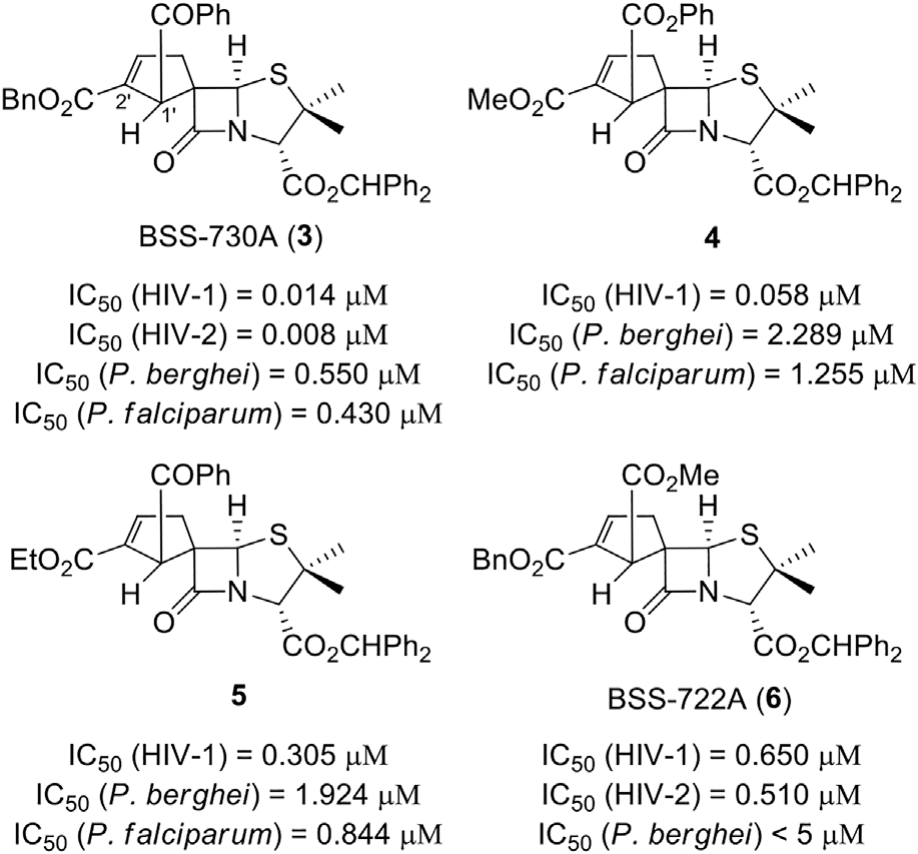
Spirocyclopentene-β-lactams with potent activity against HIV and *Plasmodium*.

Among the new chiral spiropenicillanates, spirocyclopentene-β-lactam BSS-730A (**3**) stood out by presenting a bioactivity profile characterized by a nanomolar activity against both HIV-1 and HIV-2 (IC_50 HIV-1_ = 0.014 μM and IC_50 HIV-2_ = 0.008 μM, respectively), and a submicromolar activity against *Plasmodium berghei* hepatic infection (IC_50_ = 0.550 μM) and *Plasmodium falciparum* erythrocytic infection (IC_50_ = 0.430 μM) (Figure 2) (Bártolo et al., 2021). The antiviral target and mechanism of action of BSS-730A (**3**) remain elusive, as the compound has no HIV antiprotease activity and acts equally well against HIV whether it is added before or at different time-points after cell infection. Moreover, no resistance mutations could be selected in HIV in the presence of BSS-730A. These results, together with the potent activity of BSS-730A against two very different microorganisms (a virus and a parasite), indicate that the molecule may act on the host cells rather than on the viruses/parasites.

BSS-730A was the starting point for the pursuit of new bioactive spirocyclopentene-β-lactamic derivatives. In the first instance, a limited number of structural modulations were carried out, allowing a preliminary, yet relevant, qualitative structure–activity relationship (SAR) evaluation of the spirocyclopentene-β-lactams class (Alves et al., 2021c). This evaluation demonstrated that BSS-730A (**3**) benzhydryl ester appear to play a crucial role for the molecule bioactivity, as its removal led to an inactive derivative. On the other hand, the endowment of position 2′ of BSS-730A’s spirocyclopentene ring with small alkyl esters (*i.e.* methyl and ethyl ester) led to highly bioactive compounds **4** and **5** (IC_50 HIV-1_ < 0.3 μM). A similar behavior was observed when the benzoyl group of BSS-730A (**3**) was replaced by a methyl ester in molecule BSS-722A (**6**), (Bártolo et al., 2021), although its replacement by a bulkier benzyl group lead to a total loss of activity (Alves et al., 2021c). In addition, similar to lead molecule BSS-730A (**3**), spirocyclopentene-β-lactams **4**, **5** and BSS-722A (**6**) also display anti-*Plasmodium* activity.

Overall, positions 1′ and 2′ of the cyclopentene ring in the spirocyclic core of BSS-730A (**3**) were suitable for structural modulation without compromising the resulting spirocyclopentene-β-lactams bioactivity. Thus, the present work constitutes a step forward in the development of a new class of broad-spectrum antimicrobials, through the synthesis of a family of new chiral spiro-β-lactams. The rational structural modulation of the highly promising lead molecule BSS-730A (**3**) led to the synthesis of 68 new spirocyclic penicillanates through the individual and cumulative modulation of positions 1′ and 2′ of BSS-730A’s (**3**) spirocyclopentene ring. Among the entire set of new molecules presented herein, 41 were evaluated for their cytotoxicity and anti-HIV-1 activity. The eight compounds most active against HIV-1 were further evaluated for their anti-HIV-2 and anti-*Plasmodium* activities*.* The results obtained show unequivocally the potential of spirocyclopentenepenicillanates development as a new generation of innovative antimicrobials with broad spectrum activity.

## 2 Results and discussion

### 2.1 Spirocyclopentene-β-lactams synthesis

**FIGURE 3:**
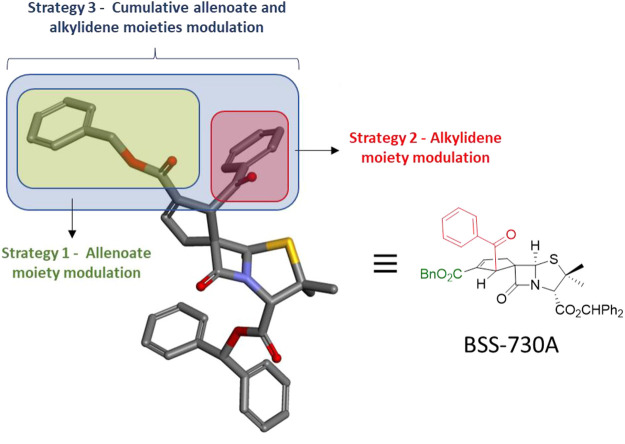
Designed structural modulation of lead molecule BSS-730A (**3**).

The strategy for the structural modulation focused on lead molecule BSS-730A (**3**) comprised the endowment of two of its cyclopentene ring positions (1′ and 2′), with different functional groups. These functional groups included bulky alkyl esters, varied aryl esters, esters containing a propargyl or a cinnamyl group, and benzoyl groups. The aromatic rings were endowed with a variety of substituents, mainly at the *para* position. This strategy aimed both at the synthesis of new bioactive spirocyclopentenepenicillanates and the gathering of significant amount of information regarding structure–activity relationships. As illustrated in Figure 3, the general strategy comprised three rounds of structural modulation of lead spiropenicillanate BSS-730A (**3**), namely: 1) the first round focused on the exclusive modulation of position 2′ of the BSS-730A (**3**) cyclopentene ring (Structural Modulation Strategy 1); 2) the second round focused on the exclusive modulation of position 1′ of the BSS-730A (**3**) cyclopentene ring (Structural Modulation Strategy 2) and; 3) the third round where those structural modulations from Synthetic Strategies 1 and 2 related with higher anti-HIV-1 activities were combined, affording new compounds with structural modulations at both positions 1′ and 2’ of the BSS-730A (**3**) cyclopentene ring (Structural Modulation Strategy 3).

**Scheme 1:**
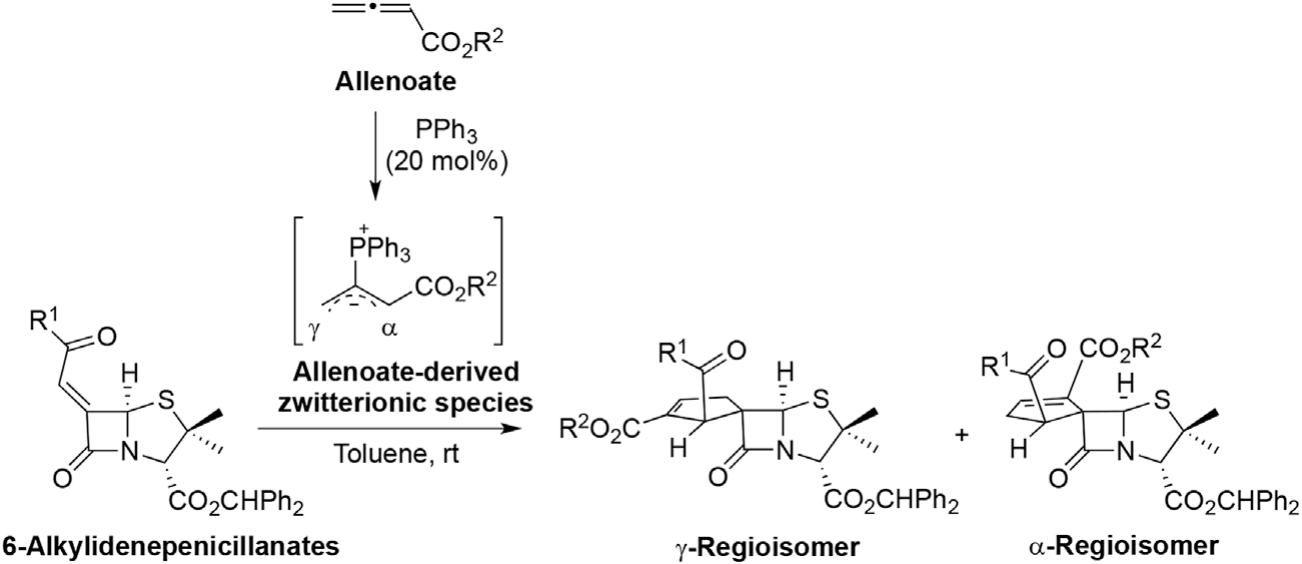
Phosphine-catalyzed [3+2] annulation of allenoates with 6-alkylidenepenicillanates as the 2π-component.

It is known that phosphine-catalyzed [3+2] annulations of allenoates with electron-deficient alkenes are an effective strategy for the synthesis of cyclopentene derivatives (Guo et al., 2018; Meng et al., 2022; Song et al., 2022). In the presence of phosphines, allenoates act as three-carbon synthons giving regioisomeric cyclopentenes upon formal [3+2] cycloaddition with asymmetrically substituted alkenes. When applied to exocyclic alkenes, this synthetic strategy provides an excellent approach for the synthesis of spirocyclic compounds. In this context, we reported for the first time the phosphine-catalyzed [3+2] cycloaddition of allenoates to 6-alkylidenepenicillanates as a route to chiral spirocyclopentene-β-lactams (Scheme 1) (Santos and Pinho e Melo, 2013; Alves et al., 2021c). The annulation reaction occurs *via* conjugate addition of the allenoate-derived zwitterionic species to the 6-alkylidenepenicillanates, followed by ring closure, proton transfer, and triphenylphosphine elimination reaction. The formation of α- and/or γ-regioisomers will depend on the initial attack of the dipole at C-6 of the β-lactam, which can involve either the α- or the γ-position. It is noteworthy that due to the inherent chirality of the penicillin-derived alkylidenes and its “butterfly”-like conformation, the approach of the formal 1,3-dipole to the 6-alkylidenepenicillanate is through its less hindered α-side, affording the spirocyclopentene-β-lactams in a diastereoselective manner.

As previously stated, the first structural modulation strategy of BSS-730A (**3**) focused on the exclusive modulation of position 2′ of the lead molecule cyclopentene ring. Such purpose was achieved by varying the ester group of the monosubstituted allenoate **8** used as dipole precursor in the phosphine-catalyzed [3+2] annulations with chiral 6-alkylidenepenicillanate **7** (Table 1). The allenoates were obtained in moderate to high yields (46%–85%) through a known procedure, (Mo et al., 2014), in a process that involves a 3-step synthetic sequence, which starts with the reaction of bromoacetyl bromide with an alcohol to give an α-bromoacetate, which reacts with triphenylphosphine to afford the corresponding phosphorous ylide. The final step is the Wittig reaction of the latter with ketene, generated *in situ* from acetyl chloride and triethylamine, to afford the expected monosubstituted allenoates (Scheme S01). The enantiopure spirocyclic products **9** and **10** were obtained in moderate to excellent overall yields (47%–98%), and the reaction time varied from 3 to 7 h, except in the case of reaction with allenoate **8n**, containing (*E*)-cinnamyl ester, which needed 72 h to be completed. It should also be noted that five cycloaddition reactions were carried out using a mixture of allenoate with the corresponding 2-butynoate isomer. However, this was not considered a potential problem because, as previously described in the literature (Zhang and Lu, 1995; Mbofana and Miller, 2014), the treatment of 2-butynoates with triphenylphosphine is an alternative to the use of allenoates as 1,3-dipole precursors. Concerning the cycloaddition regioselectivity, α-regioisomer **10** was always obtained as a major product.

**TABLE 1:** Structural modulation strategy focused on the exclusive modulation of the position 2′ of the lead molecule cyclopentene ring.

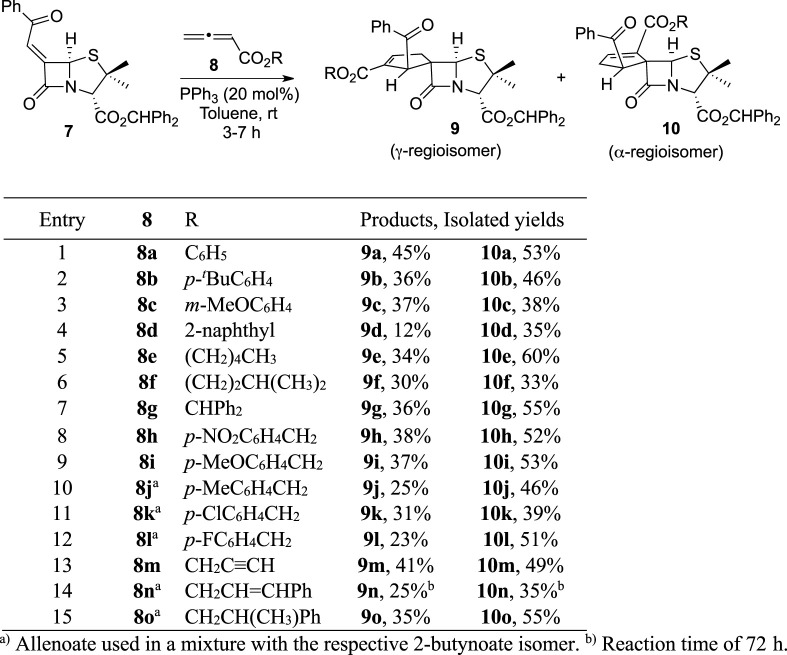

A second structural modulation focused on the substituents of position 1’ cyclopentene ring was carried out (Table 2). The synthesis of these new derivatives was achieved by exploring the annulation reaction of a library of chiral alkylidenepenicillanates **11** with benzyl allenoate (**8p**). By carrying out the reaction in the presence of triphenylphosphine at room temperature for a few hours, the expected cycloadducts **12** and **13** were obtained in good to excellent overall yields, ranging from 66% to 97%. Unfortunately, spiro-lactams **12g/13g** and **12j**/**13j** could not be separated by flash chromatography and were obtained as an inseparable mixture of regioisomers. Curiously, this modulation on the alkylidene moiety led to a different regioselectivity, with γ-regioisomer being obtained as major product in some reactions. This trend was more pronounced in reactions, which were carried out with alkylidenes bearing strong electron-withdrawing substituents (eg., *p*-NO_2_, *p*-CF_3_, and 3,5-(CF_3_)_2_), indicating that such aromatic substituents may act as regioselectivity inducers within this specific reactivity context.

**TABLE 2:** Structural modulation strategy focused on the exclusive modulation of the position 1′ of the lead molecule cyclopentene ring.

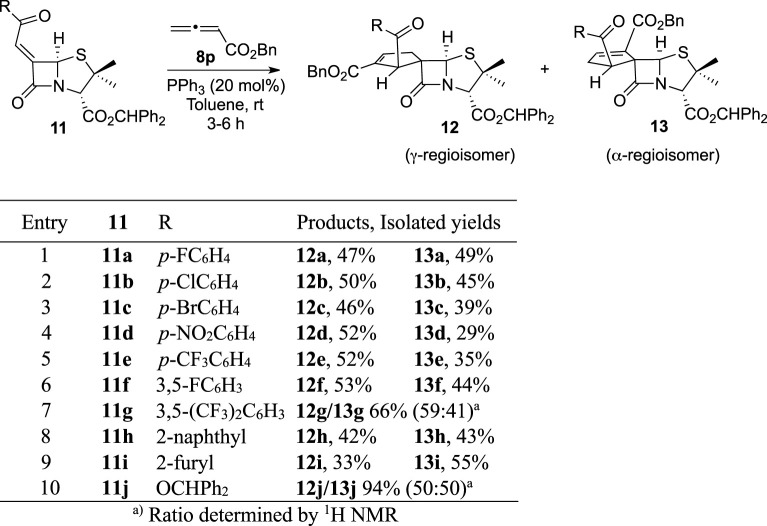

Next, a third modulation was carried out, aiming the conjugation of both previously described modulation approaches (Table 3). In fact, this last modulation was designed aiming the synthesis of spiro-β-lactams, which includes on positions 1′ and 2′ a combination of substituents present in the compounds synthesized *via* Strategies 1 and 2, with higher anti-HIV-1 activity. This allowed the simultaneous structural modulation on both positions 1′ and 2′ of the BSS-730A (**3**) cyclopentene ring.

**TABLE 3:** Structural modulation strategy focused on the exclusive modulation of both positions 1′ and 2′ of the lead molecule cyclopentene ring.

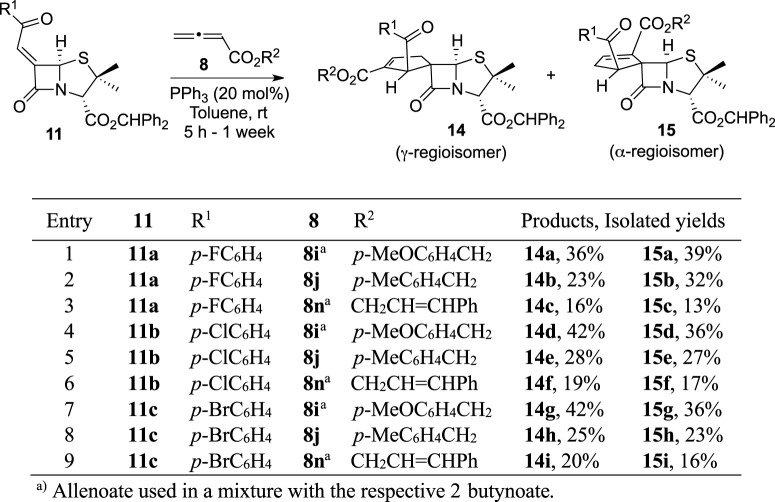

The formal cycloaddition reaction of alkylidenes **11a–c** with allenoates **8i**, **8j**, and **8n** afforded the corresponding chiral spirocyclic adducts **14** and **15**. Unfortunately, (*E*)-cinnamyl ester derivatives **14c** and **15c** were obtained in low overall yield. On the other hand, moderate to good overall yields were observed for *p*-methoxybenzyl and *p*-methylbenzyl derivatives. It is noteworthy that in comparison with the previously described reactions, these required longer reaction times, up to 1 week.

### 2.2 Biological assays

The cytotoxicity and antiviral assays against HIV-1 were performed in two rounds. The first round comprised the spiropenicillanates synthesized through rational structural modulation of positions 1′ and 2′ of BSS-730A’s cyclopentene ring (Structural Modulation Strategies 1 and 2, respectively). The results obtained in the first round guided the rational design and subsequent synthesis of new compounds, which were evaluated against HIV-1 in a second round. The compounds with more potent activity against HIV-1 (IC_50 HIV-1_ < 50 nM) were further tested against HIV-2 and *Plasmodium*.

Concerning the antiviral assays, the maximum percentage of inhibition (MPI) of all the compounds was determined at 25 μM. Only those showing MPI values higher than 80% were considered active and their IC_50_ and IC_90_ values were determined in dose-response curves.

#### 2.2.1 Anti-HIV activity

##### 2.2.1.1 Cytotoxicity

Before the antiviral activity screening, all spirocyclopentene-β-lactams were assayed for their *in vitro* cytotoxic activity (CC_50_) in TZM-bl cells (Table 4).

**TABLE 4:** Activity of spiro-β-lactams against HIV-1.

Compound	CC_50_ μM	MPI (%) 25 μg/mL	IC_50 HIV-1_ μM	IC_90 HIV-1_ μM	Therapeutic index (CC_50_/IC_50_)
**BSS-730A**	76.84	99	0.014	0.025	5488.57
**9a**	215.46	0	Nd	Nd	Nd
**9b**	54.67	0	Nd	Nd	Nd
**9c**	58.13	0	Nd	Nd	Nd
**9d**	38.02	54	Nd	Nd	Nd
**9e**	60.29	44	Nd	Nd	Nd
**9f**	53.40	21	Nd	Nd	Nd
**9g**	45.28	0	Nd	Nd	Nd
**9h**	55.99	0	Nd	Nd	Nd
**9i**	35.78	100	0.015	0.020	2385.33
**9j**	60.03	93	0.089	0.209	674.49
**9k**	54.06	84	0.366	Nd	148.11
**9l**	55.83	100	0.111	0.221	502.97
**9m**	60.11	94	0.203	0.437	296.11
**9n**	62.29	100	0.017	0.046	3664.12
**9o**	44.74	0	Nd	Nd	Nd
**12a**	58.14	93	0.019	0.054	3060.00
**12b**	55.99	92	0.063	0.104	888.73
**12c**	55.02	87	0.050	0.116	1100.40
**12d**	53.00	80	0.178	0.413	297.75
**12e**	64.40	76	1.304	1.504	49.39
**12f**	70.14	88	0.132	0.585	531.34
**12h**	73.63	0	Nd	Nd	Nd
**12i**	78.30	87	0.140	0.223	559.29
**13a**	64.77	0	Nd	Nd	Nd
**13b**	63.68	0	Nd	Nd	Nd
**13c**	50.65	0	Nd	Nd	Nd
**13d**	73.79	0	Nd	Nd	Nd
**13e**	54.74	0	Nd	Nd	Nd
**13f**	58.75	0	Nd	Nd	Nd
**13h**	56.17	0	Nd	Nd	Nd
**13i**	61.21	0	Nd	Nd	Nd
**14a**	75.06	100	0.012	0.048	6255.00
**14b**	78.26	95	0.146	0.338	494.93
**14c**	77.61	100	0.015	0.018	5174.00
**14d**	133.60	100	0.015	0.019	8906.67
**14e**	100.10	94	0.211	0.720	474.41
**14f**	87.48	97	0.067	0.198	1305.67
**14g**	100.60	100	0.030	0.099	3353.33
**14h**	72.12	93	0.111	0.297	649.73
**14i**	66.87	95	0.143	0.305	467.62

None of the assayed compounds showed significant toxicity, with the CC_50_ values varying from 35.78 μM, for molecule **9i**, to 215.46 μM, for molecule **9a**. These results are concordant with previous reports in the literature (Alves et al., 2020; Bártolo et al., 2021; Alves et al., 2021c) and confirm the low *in vitro* cytotoxic profile of spirocyclopentene-β-lactams.

##### 2.2.1.2 Anti-HIV-1 activity

The anti-HIV-1 activity of the synthesized compounds (section 2.1) will be presented individually (Table 4).

The Structural Modulation Strategy 1 afforded 15 new spirocyclopentene-β-lactamic γ-regioisomers, analogs of lead molecule BSS-730A, of which six (**9i–n**) showed activity against HIV-1 virus (MPI at 25 μg/ml > 80%). Three molecules (**9i**, **9l**, and **9n**) presented an MPI value of 100%. The six identified bioactive molecules displayed submicromolar IC_50_ values against HIV-1 with spiro-β-lactam **9k** being the less active with IC_50 HIV-1_ of 0.366 μM. The most active molecules of the set were spiro-β-lactams **9i** and **9n**, with IC_50 HIV-1_ values of 0.015 µM and 0.017 µM, respectively, values comparable to the ones presented by BSS-730A (IC_50__HIV-1_ = 0.014 µM). Furthermore, molecule **9i** presented an IC_90 HIV-1_ of 0.020 µM, a value slightly lower than BSS-730A (IC_90__HIV-1_ = 0.025 µM). The antiviral activity of spiro-β-lactams **10** was not evaluated because previous studies showed that α-regioisomers derived from modulation of position 2’ of cyclopentene ring did not lead to active compounds (Santos and Pinho e Melo, 2013; Alves et al., 2021c).

Next, anti-HIV-1 activity of both γ- and α-regioisomers (**12** and **13**) derived from the modulation of the position 1′ of the lead molecule was accessed. Among these novel 16 derivatives, seven γ-regioisomers (**12**) showed interesting anti-HIV-1 activity. The most active molecule of this set was spiro-β-lactams **12a** with an IC_50 HIV-1_ value of 0.019 μM. However, it was possible to identify five more molecules with IC_50 HIV-1_ values under 0.200 μM. On the other hand, no anti-HIV activity was observed for α-regioisomers **13**, a behavior concordant with what has been previously observed for similar spirocyclopentenepenicillanates (Bártolo et al., 2021).

Notably, nine derivatives comprising the simultaneous structural modulation on both positions 1′ and 2′ of the BSS-730A (**3**) cyclopentene ring showed high inhibition values (MPI at 25 μg/ml > 90%) and low IC_50 HIV-1_ values (compounds **14a**–**14i**). Three molecules (**14a**, **14c**, and **14d**) should be highlighted for their IC_50 HIV-1_ values under 0.020 μM. These molecules have similar or lower IC_50 HIV-1_ values when compared with our lead molecule BSS-730A [BSS-730A (IC_50 HIV-1_ = 0.014 µM), **14a** (IC_50 HIV-1_ = 0.012 µM), **14c** (IC_50 HIV-1_ = 0.015 µM), and **14d** (IC_50 HIV-1_ = 0.015 µM)]. Moreover, molecules **14c** and **14d** showed lower IC_90 HIV-1_ values than BSS-730A (IC_90 HIV-1_ = 0.025 µM), 0.018 µM and 0.019 µM, respectively.

##### 2.2.1.3 Anti-HIV-2 activity

In order to get more information about the antiviral activity of our compounds, the eight compounds with better anti-HIV-1 activity were evaluated against HIV-2 (Table 5). All compounds were also very active against HIV-2 with an inhibition of 100% of viral replication at 25 μg/ml and IC_50 HIV-2_ values, ranging from 0.011 to 0.111 μM (Table 5). Spiro-β-lactams **12a**, **14a**, and **9n** were the most active with IC_50 HIV-2_ values of 0.011, 0.013, and 0.016 µM, respectively. It should also be highlighted that compounds **12a** and **14a** showed lower IC_90_ values (0.055and 0.051 µM, respectively) against HIV-2 virus than lead molecule BSS-730A (**3**) (IC_90 HIV-2_ = 0.064 µM) (Bártolo et al., 2021).

**TABLE 5:** Activity of selected spiro-β-lactams against HIV-2 (structures of the studied compounds are also included).

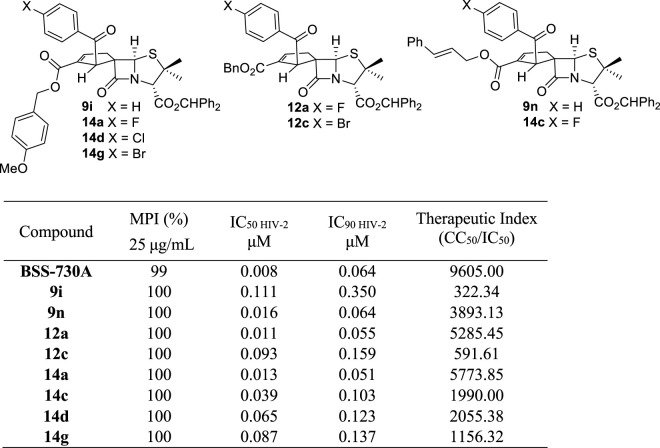

#### 2.2.2 Anti-*Plasmodium* activity

##### 2.2.2.1 Hepatic stage of infection

The anti-*Plasmodium* activity of the set of eight spirocyclopentene-β-lactams most active against HIV-1 was also determined. In a preliminary activity screen, the percentage of inhibition of hepatic infection by the rodent *P. berghei* parasite was determined at 1 and 10 μM (Supplementary Material, Supplementary Figure S94), for the entire set of compounds. The results showed that none of the compounds tested displayed cytotoxicity against the Huh-7 host cells. All compounds displayed potent inhibition at 10 μM, while compounds **9i**, **12a**, and **14a** showed relatively low inhibitory activity at 1 μM (lower than 50%).

Given these encouraging results, the IC_50_ values of six spirocyclic derivatives against *P. berghei* hepatic infection were determined and were found to range from 0.285 to 2.593 μM (Table 6). The lowest activity was observed for compound **14g**, with an IC_50__*P. berghei*_ of 2.593 μM, followed by compounds **14d** (IC_50__*P. berghei*_ = 1.997 μM) and **9n** (IC_50__*P. berghei*_ = 1.575 μM). Of note, three of the spirocyclopentene-β-lactams screened showed submicromolar activity against the parasite’s hepatic stage of infection, with compound **9i** displaying the highest activity (IC_50__*P. berghei*_ = 0.285 μM), two-fold more potent than the lead molecule BSS-730A (**3**) (IC_50__*P. berghei*_ = 0.550 μM).

**TABLE 6:** Compound activity against the *P. berghei* hepatic stage.

Compound	IC_50__*P. berghei*_ μM
**BSS-730A**	0.550 ± 0.14
**9i**	0.285 ± 0.044
**9n**	1.575 ± 0.191
**12a**	0.924 ± 0.027
**12c**	Between 1 and 10
**14a**	0.750 ± 0.107
**14c**	Between 1 and 10
**14d**	1.997 ± 0.458
**14g**	2.593 ± 0.219

##### 2.2.2.2 Blood stage of infection

Due to its promising submicromolar activity against hepatic infection by *Plasmodium*, the activity of the three most promising spirocyclopentene-β-lactams (**9i**, **12a**, and **14a**) against the erythrocytic stage of *P. falciparum* infection was further assessed (Table 7). The three compounds showed remarkable activity, with both compounds **9i** and **14a** showing higher activity than BSS-730A (**3**) against this phase of the parasite’s life cycle, with IC_50__*P. falciparum*_ values of 0.336 and 0.292 μM, respectively. On the other hand, the lowest activity was observed for **12a**, with an IC_50__*P. falciparum*_ of 0.560 μM, a value nevertheless comparable to the one presented by the lead molecule BSS-730A (**3**) (IC_50__*P. falciparum*_ = 0.430 μM).

**TABLE 7:** Compound activity against the *P. falciparum* blood stage.

Compound	IC_50__*P. berghei*_ μM
**BSS-730A**	0.430 ± 0.04
**9i**	0.336 ± 0.067
**12a**	0.560 ± 0.126
**14a**	0.292 ± 0.062

### 2.3 Structure–activity relationships

#### 2.3.1. Anti-HIV activity

The synthesis and antiviral activity determination of 40 different BSS-730A’s new analogs allowed an extensive and qualitative analysis of structure–activity relationships of this family of spirocyclopentenepenicillanates as HIV inhibitors.

Contrary to what was observed for small alkyl ester (*i.e.,* methyl and ethyl esters) (Alves et al., 2021c), the introduction of longer chain alkyl esters like pentyl and isopentyl esters at position 2′ of the cyclopentene ring led to molecules with no relevant anti-HIV activity. These results demonstrate that the antiviral activity of this class of spirocyclic lactams is only compatible with a limited chain length of alkyl substituents on the referred position. Aryl esters at the same position 2′ of the cyclopentene ring also proved to fully compromise the respective spirocyclopentenepenicillanates antiviral activity against HIV. Although containing a benzyl ester as substructure, benzhydryl ester at position 2′ of the cyclopentene ring also caused a total loss of anti-HIV activity, the behavior potentially imputable to the presence of an additional phenyl group leading to higher bulkiness of this substituent, which may preclude an effective interaction between the ligand and its putative biological target.

The inclusion of *para* substituents with electron-donating and withdrawing properties in the aromatic ring of the benzyl ester group has proven to be a successful strategy to obtain new BSS-730A derivatives with potent antiviral activity. Among the five new spirocyclopentenepenicillanates obtained through this structural modulation (**9h**–**9l**), four displayed submicromolar activity against HIV-1 (IC_50 HIV-1_ < 0.4 μM). Considering compounds **9h**–**l** it was possible to establish a direct correlation between the magnitude of the electron effect of the *para* substituent (NO_2_ < Cl < F < Me < MeO) (Hansch et al., 1991) and the observed anti-HIV-1 activity (**9h** < **9k** < **9l** < **9j** < **9i**). The presence of the highly electron-withdrawing NO_2_ group led to an inactive molecule **9h**, while molecule **9i** containing a *para*-methoxy substituent, the most electron-donating substituent of the series, presents the higher activity against HIV (IC_50 HIV-1_ = 0.015 μM). This trend indicates that a benzyl ester group at cyclopentene’s 2′ position incorporating an electron-rich aromatic ring potentiates the antiviral activity of spirocyclopentenepenicillanates. Interestingly, adding conjugation to the phenyl group, going from benzyl ester derivative BSS-730A (**3**) to cinnamyl ester derivative **9n** had also a positive impact, the latter molecule displaying nanomolar anti-HIV-1 activity.

Molecule, **9m** containing a propargyl ester, should also be highlighted. Despite being one of the less active molecules resulting from Structural Modulation Strategy 1, it still presents a remarkable IC_50_ of 0.203 μM against HIV-1. Although the terminal alkyne of propargyl ester is also an electron-rich residue, spiropenicillanate **9m** lower antiviral activity may be explained by the fact that propargyl ester is unable to participate in π–π stacking interactions. However, the presence of a terminal alkyne on a yet highly active compound gives the opportunity to explore azide–alkyne click chemistry for the synthesis of conjugates (Luo et al., 2014; Kaur et al., 2021).

Through structural modulation of 6-alkylidenepenicillanates precursors, the addition of halogens to the benzoyl group of BSS-730A (**3**) was also performed. It was observed that compounds **12a**–**c** bearing weak withdrawing groups (R = F, Cl, and Br) showed higher anti-HIV-1 activity than compounds **12d**,**e** with strong withdrawing substituents (R = NO_2_ and CF_3_).

The *para* nitro aromatic substituent is the only substituent common to Structural Modulation Strategies 1 and 2 that leads to different outcomes when placed on the benzoyl or the benzyl ester group of lead molecule BSS-730A (**3**). While on the benzoyl group of BSS-730A (**3**), the nitro substituent leads to a slight decrease of the antiviral activity (molecule **12d**, with submicromolar activity IC_50 HIV-1_ = 0.178 μM), the same substituent causes a total loss of antiviral activity when present on the benzyl ester group (molecule **9h**). Concerning the rational design of new potentially bioactive spirocyclopentene-β-lactams, this result demonstrates that the benzoyl group at cyclopentene’s 1′position is more tolerant to the inclusion of highly electron-withdrawing substituents than the benzyl group at cyclopentene’s 2′position.

Although being 10 times less active than lead molecule BSS-730A, the fact that 1′-furoyl-substituted molecule **12i** displays a remarkable submicromolar antiviral activity against HIV-1 (IC_50 HIV-1_ = 0.140 μM), which is an important outcome, as it shows that cyclopentene position 1′ can have its ketone group modulated by the incorporation of heteroaromatic rings without causing an abrupt loss of antiviral activity.

From the simultaneous structural modulation on both positions 1′ and 2′ of the BSS-730A cyclopentene ring, interesting conclusions can be drawn. It was possible to observe that the combined introduction of *p*-halobenzoyl groups at position 1′ together with the introduction of a *p*-methylbenzyl ester group at position 2′ (see compounds **14b**, **14e**, and **14h**) did not increase the antiviral activity when compared to the outcome of the changes carried out separately (see compounds **9j**, **12a**, **12b**, and **12c**). However, combining *p*-halobenzoyl groups at spirocyclopentenepenicillanates’ 1′position with the introduction of a benzyl ester group at position 2′ with a strong electron donating substituent (*p*-methoxybenzyl ester group) led to compounds (**14a**, **14d**, and **14g**) as highly active as the lead molecule BSS-730A. The molecule **14c**, containing a *p*-fluorobenzoyl group at position 1′ and a cinnamyl ester at position 2′, also led to very interesting results, corroborating the importance of adding conjugation to the phenyl group at position 2′ to achieve high anti-HIV activity.

**FIGURE 4:**
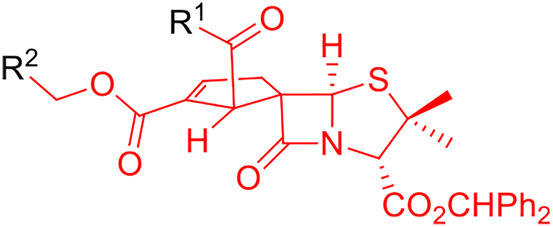
Common substructure of all bioactive spirocyclopentenepenicillanates.

Overall, the qualitative structure–activity relationship results discussed herein show that the presence of electron-rich aromatic groups in both positions 1′ and 2′ of the cyclopentene ring are associated with higher anti-HIV-1 activity. It was also possible to establish the crucial relevance of a methylene group in the ester substituent at position 2′ of the cyclopentene ring (CO_2_CH_2_-R) because it is a common substructure of all the bioactive spirocyclopentenepenicillanates previously (Bártolo et al., 2021; Alves et al., 2021c) and presently identified (Figure 4). This structure–activity relationship is also corroborated by the lack of antiviral activity observed for spirocyclopentene-β-lactam **9a**, in which the molecular structure differs from lead molecule BSS-730A (**3**) exclusively by the absence of this methylene group, since molecule **9a** has a phenyl ester at position 2’ while BSS-730A (**3**) has a benzyl ester.

Nine spirocyclopentenepenicillanates were evaluated as anti-HIV-2 agents, all of them displaying nanomolar activity. Despite the extraordinary results, the presence of the *p*-methoxybenzyl ester group at spirocyclopentene-β-lactams’ 2′position does not have a similar impact as the one observed for the anti-HIV-1 activity, leading to a slight decrease of the anti-HIV-2 activity (compounds **9i**, **14a**, **14d**, and **14g**). Molecule **9n** containing a cinnamyl ester in position 2′ displayed high anti-HIV-2 activity, with an IC_50 HIV-2_ value of 0.016 μM similar to the one shown against HIV-1.

The importance of a fluorine atom to enhance the anti-HIV-2 activity of the synthesized molecules is noteworthy. Three of the four molecules with higher anti-HIV-2 activity contain a *p*-fluorobenzoyl group at position 1’ [**12a** (IC_50 HIV-2_ = 0.011 μM), **14a** (IC_50 HIV-2_ = 0.013 μM), and **14c** (IC_50 HIV-2_ = 0.039 μM)]. It should be highlighted that fluorine atoms are well known bioisosteres of hydrogen atoms being the introduction of fluorine into a molecule a strategy used in the drug design to improve drug-like features (Gillis et al., 2015). This rational drug design strategy has already proven valuable on the drug development of other β-lactamic core containing drugs, namely, a series of orally active cholesterol absorption inhibitors (Rosenblum et al., 1998).

#### 2.3.2. Anti-*Plasmodium* activity

The analysis of the inhibitory activity of spirocyclopentene-β-lactams against *Plasmodium* hepatic infection indicates that the addition of a halogen to the benzoyl aromatic ring at 1’ position causes a decrease of activity against the *Plasmodium* hepatic infectious stage when compared to its counterparts without aromatic substituents on such moiety. This behavior can be clearly observed in the series of compounds **9i**, **14a**, **14d**, and **14g** and in the series of compounds **3**, **12a**, and **12c**.

Unlike what was demonstrated for both HIV-1 and HIV-2 viruses, the substitution of the benzyl ester on BSS-730A (**3**) by a cinnamyl ester led to a three-fold decrease of the molecule’s inhibitory activity against the parasite hepatic infectious stage. One can speculate that such result may be attributed to the increasing of the ester group chain length, which can obstruct an effective molecule-target interaction.

On the other hand, it is noteworthy that the presence of a *p*-MeO group on benzyl ester at position 2′ of the lead molecule’s cyclopentene ring leads to a two-fold increase on its hepatic stage antiplasmodial activity [**9i** (IC_50__*P. berghei*_ = 0.285 μM) vs. BSS-730A (**3**) (IC_50__*P. berghei*_ = 0.550 μM). However, a positive effect of the benzyl ester’s *p*-MeO substituent on the molecules’ activity can also be observed for the *p*-fluorobenzoyl derivatives **14a** (IC_50__*P. berghei*_ = 0.750 μM) and **12a** (IC_50__*P. berghei*_ = 0.924 μM).

The study on the antiplasmodial activity during the parasite’s blood stage of spirocyclopentene-β-lactams **9i**, **12a**, and **14a** also allows to carry out a qualitative structure–activity analysis. Comparing to BSS-730A (**3**) activity (IC_50__*P. falciparum*_ = 0.430 μM), it was observed that the introduction of a *p-*MeO substituent on benzyl ester aromatic ring potentiates the molecule’s antiplasmodial activity [**9i** (IC_50__*P. falciparum*_ = 0.336 μM)], an effect that it is even more pronounced when combined with the presence of a *p-*F substituent on the molecule’s benzoyl group [**14a** (IC_50__*P. falciparum*_ = 0.292 μM)]. However, in the absence of the methoxy aromatic substituent, the presence of the fluorine substituent at the benzoyl group *para* position *per se* leads to a slight decrease in the molecule antiplasmodial activity [**12a** (IC_50__*P. falciparum*_ = 0.560 μM)].

**FIGURE 5:**
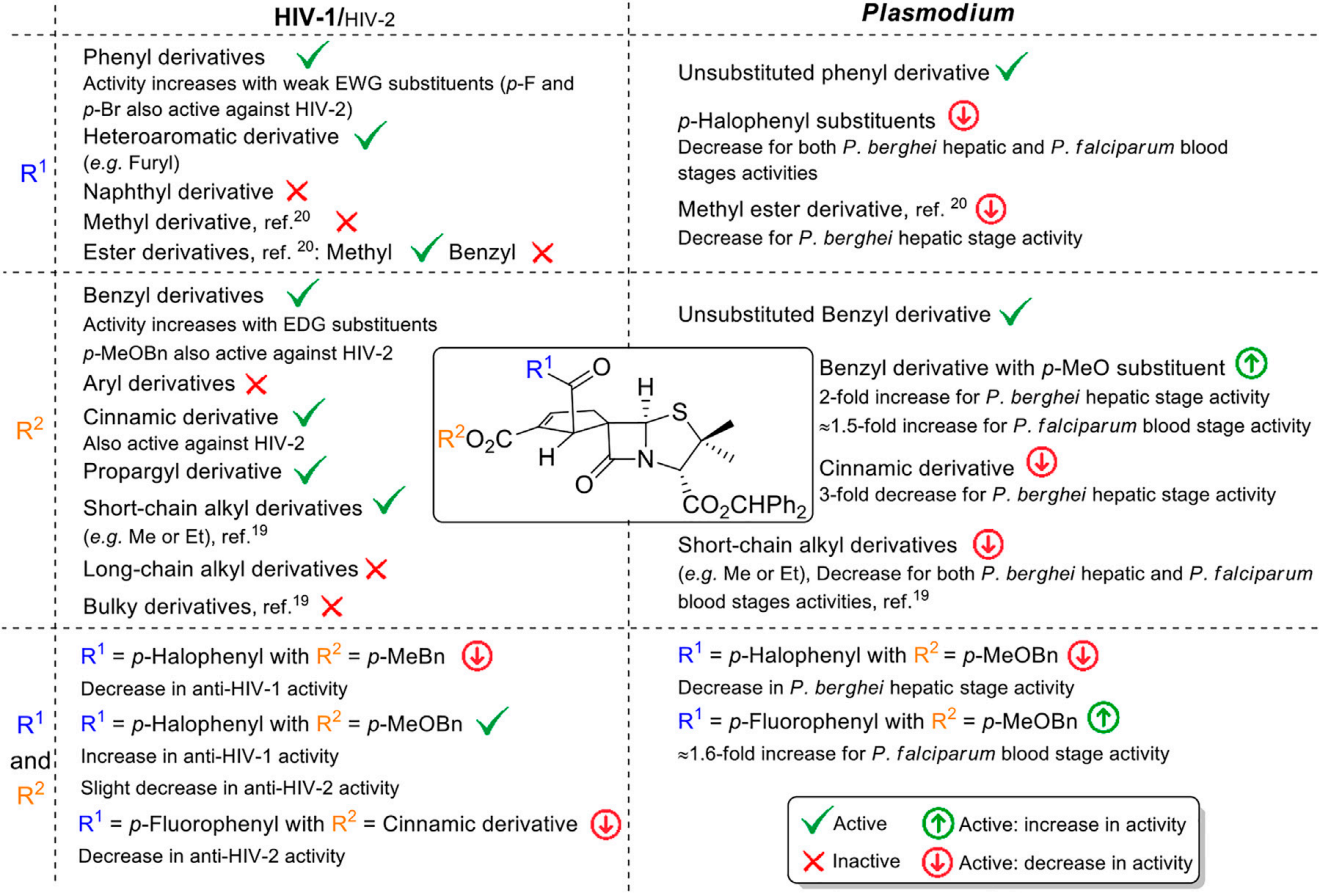
Overview of the identified structure–activity relationships.

Overall, the identified qualitative structure–activity relationships concerning the antimicrobial activity of the molecules developed along the present work represent an important achievement for the future rationale design of new spirocyclopentene-β-lactams with potential broad spectrum antimicrobial activity (Figure 5).

## 3 Conclusion

A library of 68 new spirocyclopentene-β-lactams was synthesized through the rational drug design and structure modulation of a highly promising lead molecule BSS-730A. The strategy focused mainly on the endowment of two of the cyclopentene ring positions with different functional groups either by replacing the current substituents with bulkier alkyl esters, varied aryl esters, and esters moieties incorporating double- or triple carbon-carbon bonds (cinnamyl and propargyl esters), or by including substituents, mainly in *para* position, on the terminal aromatic rings of such positions.

All the screened molecules showed low cytotoxicity against TZM-bl cell line, with CC_50_ values ranging from 35.78 to 215.46 μM. Twenty-two new spirocyclopentene-β-lactams inhibited HIV-1 replication, with IC_50 HIV-1_ values as low as 0.012 μM. The derivatives with electron-rich aromatic groups in both positions 1′ and 2′ displayed higher anti-HIV-1 activity. Among those, the eight compounds with higher anti-HIV-1 activity (IC_50 HIV-1_ < 0.050 μM) were also active against HIV-2 and *Plasmodium* hepatic and blood infectious stage. The most active compound against HIV-2 showed a remarkable IC_50 HIV-2_ value of 0.011 μM. It should be highlighted that among the four molecules with higher anti-HIV-2 activity three of them contain a *p*-fluorobenzoyl group at position 1′.

Concerning the compounds’ antiplasmodial activities, the most active spiro-β-lactam against the *Plasmodium* infectious hepatic stage have an IC_50__*P. berghei*_ value of 0.285 μM, while the spiro-β-lactam which displayed the lower IC_50_ against the parasite blood stage showed an IC_50__*P. falciparum*_ value of 0.267 μM. Both the anti-HIV and antiplasmodial results presented herein represent an improvement toward the previously observed bioactivity of lead molecule BSS-730A. Given the high structural similarity between BSS-730A and its new bioactive analogs, it is estimated that the latter ones also share the lead molecule innovative antimicrobial host-based mechanism of action.

As a result of the wide pool of screened compounds, it was also possible to gather a considerable and representative amount of qualitative structure–activity information regarding the spirocyclopentene-β-lactams family of compounds, providing a strong background of structural and pharmacophoric information for further rational design and synthesis of new potentially bioactive molecules.

Altogether, the present work comprises a successful strategy of rational structure modulation and optimization of a lead molecule, allowing the identification of new twenty-two highly bioactive spirocyclopentene-β-lactams, three of which display improved antimicrobial activity against HIV and/or *Plasmodium* when compared to BSS-730A.

## 4 Experimental section

### 4.1 Chemistry

Thin-layer chromatography (TLC) analyses were performed using precoated silica gel plates. Flash column chromatography was performed with silica gel 60 as the stationary phase. ^1^H nuclear magnetic resonance (NMR) spectra (400 MHz) and ^13^C NMR spectra (100 MHz) were recorded in CDCl_3_ or hexadeuterated dimethyl sulfoxide (DMSO-d_*6*_). Chemical shifts are expressed in parts per million (ppm) relatively to internal tetramethylsilane (TMS), and coupling constants (J) are expressed in hertz (Hz). Infrared spectra (IR) were recorded in a Fourier transform spectrometer coupled with a diamond attenuated total reflectance (ATR) sampling accessory. Elemental analyses were carried out with an Elemental Vario Micro Cube analyzer. High-resolution mass spectra (HRMS) were obtained on a TOF VG Autospect M spectrometer with electrospray ionization (ESI). Melting points (mp) were determined in open glass capillaries. Optical rotations were measured on an Optical Activity AA-5 electrical polarimeter. 6-Alkylidenepenicillanates **7** and **11** were synthesized as described in the literature (Santos and Pinho e Melo, 2013; Alves and Pinho e Melo, 2020). Full characterization of allenoates **8a**–**d** and **8f**–**o** (the synthesis of allenoates **8a–d** (Himbert et al., 1988; Himbert et al., 1989; Conner and Brown, 2016) and **8m** (Conner and Brown, 2016) has been previously described) and spiro-β-lactams **9b–o**, **10b–o**, **12b–j**, **13b–j**, **14b–i**, and **15b–i** can be found in the Supplementary Material.

### 4.2 General procedure for the synthesis of monosubstituted allenoates

The monosubstituted allenoates were obtained through a synthetic procedure, previously reported in the literature (Mo et al., 2014).

Bromoacetyl bromide (0.252 ml, 2.90 mmol) was added dropwise to a solution of alcohol (2.90 mmol) and pyridine (0.232 mL, 2.90 mmol) in DCM (10 mL) at 0°C, forming a white suspension. The suspension was stirred for 20 min at 0°C and then for an additional 30 min at 25°C, after which distilled water (15 mL) was added to the reaction mixture. The organic layer was separated, and the aqueous layer was then extracted with DCM (2 × 15 mL). The combined organic layers were washed with brine (15 mL), dried over with MgSO_4_, and concentrated to give the respective α-bromoacetate as oil, which was used directly without purification for the next reaction step.

The α-bromoacetate obtained in the previous step was added dropwise to a solution of triphenylphospine (0.754 g, 2.9 mmol) in toluene (20 mL) and was left stirring overnight. The resulting precipitate was filtered, washed sequentially with toluene and hexane, and then dissolved in distilled water (20 mL). NaOH (2 M) was added to maintain the pH > 7 and the mixture was left stirring. After 30 min, DCM (20 mL) was added. The organic layer was then separated, washed with brine (15 mL), dried over with MgSO_4_, and the filtrate was concentrated affording the desired phosphorus ylide, which was used directly without further purification for the next step.

The phosphorus ylide was then dissolved in anhydrous DCM (5 mL) in a two-neck round-bottled flask, and NEt_3_ (0.405 mL, 2.90 mmol) was added dropwise to the solution. After stirring for 15 min, a previously prepared solution of acetyl chloride (0.212 mL, 2.90 mmol) in anhydrous DCM (5 mL) was added dropwise over 30 min. The reaction mixture was left stirring for 12 h under nitrogen. A precipitate was formed, which was filtered and discarded, and the solvent was carefully evaporated under reduced pressure. The desired allenoate was purified by flash chromatography [ethyl acetate/hexane], being obtained as oil or a low melting point solid.

#### 4.2.1 Pentyl 2,3-butadienoate (**8e**)

Allenoate **8e** was obtained as described in the general procedure from the corresponding alcohol (pentanol) and purified by flash chromatography [ethyl acetate/hexane (1:4)], being obtained as colorless oil (0.299 g, 1.943 mmol, 67%). NMR ^1^H (400 MHz, CDCl_3_) δ = 0.89 (t, *J* = 6.8 Hz, 3H), 1.32 (m, 4H), 1.64 (m, 2H), 4.13 (t, *J* = 6.8 Hz, 2H), 5.20 (d, *J* = 6.4 Hz, 2H), and 5.62 (t, *J* = 6.4 Hz, 1H); ^13^C NMR (100 MHz, CDCl_3_) δ = 14.1, 22.4, 28.1, 28.4, 65.3, 79.3, 88.2, 166.0, and 215.9; HRMS (ESI) m/z: calculated for C_9_H_14_NaO_2_ [M + Na]^+^ 177.0886; found 177.0885.

### 4.3 General procedure for the phosphine-catalyzed [3+2] annulation of allenoates with 6-alkylidenepenicillanates

The general procedure for the synthesis of spirocyclopentene-β-lactams through phosphine-catalyzed [3+2] annulation of allenoates with 6-alkylidenepenicillanates is described in the literature (Santos and Pinho e Melo, 2013).

To a mixture of the appropriate 6-alkylidenepenicillanate (1 equiv.) and PPh_3_ (20 mol%) in toluene (2–3 mL), a solution of allene (1 equiv.) in toluene (1–2 mL) was added. The reaction mixture was stirred at room temperature under nitrogen for the time indicated in each case, being monitored through TLC. Upon completion, the solvent was removed under reduced pressure and the crude product was purified by flash chromatography.

(1′*R*,2′*R*)-Benzhydryl spiro[(2-benzoyl-3-phenoxycarbonylcyclopent-3-ene)-1′,6-penicillanate] (**9a**) and (1′*R*,2′*R*)-Benzhydryl spiro[(2-benzoyl-5-phenoxycarbonylcyclopent-4-ene)-1′,6-penicillanate] (**10a**).

Obtained from phenyl 2,3-butadienoate (**8a**) (33 mg, 0.21 mmol) and 6-alkylidenepenicillanate **7** (100 mg, 0.21 mmol) as described in the general procedure. The reaction mixture was stirred for 6 h. Purification of the crude product by flash chromatography [(ethyl acetate/hexane (1:4)] gave, in order of elution, **9a** as a colorless solid (61 g, 0.095 mmol, 46%) and **10a** as a colorless solid (71 g, 0.110 mmol, 53%).

Compound **9a**: mp > 137.0°C (with decomposition); ${\left[\alpha \right]}_{D}^{25}$ = + 340 (*c* 0.25 in CH_2_Cl_2_); IR (ATR): ν = 1,772, 1,735, 1,657, and 1,490 cm ^−1^; ^1^H NMR (400 MHz, CDCl_3_) δ = 1.13 (s, 3H), 1.53 (s, 3H), 3.21 (dd, *J* = 18.8 and 3.2 Hz, 1H), 3.64 (dt, *J* = 18.8 and 2.4 Hz, 1H), 4.56 (s, 1H), 5.27 (d, *J* = 1.2 Hz, 1H), 5.48 (s, 1H), 6.80–6.83 (m, 2H), 6.93 (s, 1H), 7.15 (m, 1H), 7.25 (br s, 1H), 7.27–7.36 (m, 12H), 7.41–7.45 (m, 2H), 7.53–7.56 (m, 1H), and 8.10–8.13 (m, 2H); ^13^C NMR (100 MHz, CDCl_3_) δ = 26.1, 32.6, 41.0, 53.0, 64.3, 69.1, 70.8, 71.2, 78.5, 121.5, 126.0, 127.1, 127.7, 128.5, 128.6, 128.8, 129.4, 129.5, 133.7, 135.6, 137.5, 139.2, 139.3, 147.6, 150.3, 161.4, 167.0, 176.4, and 201.2; HRMS (ESI) m/z: calculated for C_39_H_33_NNaO_6_S [M + Na]^+^ 666.1921; found 666.1913.

Compound **10a**: mp 92.5–95.0°C; ${\left[\alpha \right]}_{D}^{25}$ = + 420 (*c* 0.5 in CH_2_Cl_2_); IR (ATR): ν = 1,769, 1,735, 1,684, 1,591, and 1,490 cm ^−1^; ^1^H NMR (400 MHz, CDCl_3_) δ = 1.11 (s, 3H), 1.53 (s, 3H), 2.62 (dd, *J* = 19.2 and 2.4 Hz, 1H), 3.29 (ddd, *J* = 18.8, 9.2 and 2.0 Hz, 1H), 4.54 (s, 1H), 4.62 (d, *J* = 8.4 Hz, 1H), 6.34 (s, 1H), 6.89 (s, 1H), 7.12–7.20 (m, 6H), 7.24–7.30 (m, 4H), 7.35–7.42 (m, 6H), 7.50–7.54 (m, 2H), 7.61–7.64 (m, 1H), and 7.97–7.99 (m, 2H); ^13^C NMR (100 MHz, CDCl_3_) δ = 25.8, 33.1, 36.2, 49.5, 62.8, 69.6, 71.1, 74.1, 78.3, 122.0, 126.0, 127.3, 127.6, 128.0, 128.2, 128.6, 128.6, 129.1, 129.5, 133.8, 134.0, 135.1, 139.5, 139.6, 146.3, 150.6, 161.0, 166.7, 174.2, and 198.5; HRMS (ESI) m/z: calculated for C_39_H_33_NNaO_6_S [M + Na]^+^ 666.1921; found 666.1910.

(1′*R*,2′*R*)-benzhydryl spiro[(2-(4-fluorobenzoyl)-3-benzyloxycarbonylcyclopent-3-ene)-1′,6-penicillanate] (**12a**) and (1′*R*,2′*R*)-benzhydryl spiro[(2-(4-fluorobenzoyl)-5-benzyloxycarbonylcyclopent-4-ene)-1′,6-penicillanate] (**13a**).

Obtained from allene **8p** (65 mg, 0.375 mmol) and 6-alkylidenepenicillanate **11a** (188 mg, 0.375 mmol) as described in the general procedure (reaction time: 6.5 h). Purification of the crude product by flash chromatography (hexane/ethyl acetate, 4:1) gave, in order of elution, **12a** as a colorless solid (119 mg, 0.175 mmol, 47%) and **13a** as a colorless solid (125 mg, 0.185 mmol, 49%).

Compound **12a**: mp 64.5–66.5°C; ${\left[\alpha \right]}_{D}^{25}$ = + 270 (c 0.5 in CH_2_Cl_2_); IR (ATR): $\tilde{\nu }$ = 1,063, 1,155, 1,201, 1,234, 1,330, 1,456, 1,593, 1,670, 1,718, and 1,773 cm^−1^; ^1^H NMR (400 MHz, CDCl_3_): δ = 1.11 (s, 3H), 1.50 (s, 3H), 3.11 (dd, *J* = 18.6 and 3.1 Hz, 1H), 3.53 (dt, *J* = 18.6 and 2.3 Hz, 1H), 4.52 (s, 1H), 4.96 (d, *J* = 12.2 Hz, 1H), 5.00 (d, *J* = 12.2 Hz, 1H), 5.10 (d, *J* = 1.2 Hz, 1H), 5.41 (s, 1H), 6.91 (s, 1H), 7.01 (t, *J* = 8.6 Hz, 2H), 7.08 (s, 1H), 7.13–7.18 (m, 2H), 7.28–7.37 (m, 13H), and 8.07 (dd, *J* = 5.4 and 8.9 Hz, 2H); ^13^C NMR (100 MHz, CDCl_3_): δ = 26.1, 32.4, 40.8, 52.8, 64.3, 66.9, 69.1, 70.6, 71.1, 78.5, 115.6 (d, *J* = 22 Hz, 2C), 127.2, 127.7, 128.4, 128.5, 128.5, 128.6, 128.7, 128.7, 128.8, 132.1 (d, *J* = 10 Hz, 2C), 133.8 (d, *J* = 3 Hz, 1C), 135.3, 135.7, 139.2, 139.3, 146.2, 162.9, 166.1 (d, *J* = 256 Hz, 1C), 167.0, 176.3, and 199.6; ^19^F NMR (376 MHz, CDCl_3_): -104.44 (s, 1F); HRMS (ESI-TOF) m/z: [M + H]^+^ calculated C_40_H_35_FNO_6_S 676.2164; found 676.2162.

Compound **13a**: mp 73.9–75.9°C; ${\left[\alpha \right]}_{D}^{25}$ = + 430 (c 0.5 in CH_2_Cl_2_); IR (ATR): $\tilde{\nu }$ = 977, 1,012, 1,151, 1,176, 1,200, 1,306, 1,374, 1,453, 1,596, 1,679, 1,718, 1,744, and 1,776 cm^−1^; ^1^H NMR (400 MHz, CDCl_3_): δ = 1.11 (s, 3H), 1.51 (s, 3H), 2.49 (dd, *J* = 18.6 and 2.5 Hz, 1H), 3.19 (ddd, *J* = 18.6, 9.1 and 2.0 Hz, 1H), 4.50 (d, *J* = 8.4 Hz, 1H), 4.54 (s, 1H), 5.21 (s, 2H), 6.27 (s, 1H), 6.91 (dd, *J* = 2.8 and 2.2 Hz, 1H), 6.94 (s, 1H), 7.16 (t, *J* = 8.6 Hz, 2H), 7.27–7.38 (m, 11H), 7.40–7.48 (m, 4H), and 7.96 (dd, *J* = 5.3 and 8.8 Hz, 2H);

^13^C NMR (100 MHz, CDCl_3_): δ = 25.9, 32.8, 36.0, 49.4, 62.8, 66.6, 69.1, 71.0, 74.0, 78.4, 116.3 (d, *J* = 22 Hz, 2C), 127.5, 127.5, 128.2, 128.2, 128.4, 128.5, 128.7, 128.7, 131.2 (d, *J* = 9 Hz, 2C), 131.6 (d, *J* = 3 Hz, 1C), 134.4, 135.8, 139.6, 144.7, 162.5, 166.1 (d, *J* = 256 Hz, 1C), 166.7, 174.2, and 196.9; ^19^F NMR (376 MHz, CDCl_3_): -104.18 (s, 1F); HRMS (ESI-TOF) m/z: [M + H]^+^ calculated C_40_H_35_FNO_6_S 676.2164; found 676.2156.

(1′*R*,2′*R*)-benzhydryl spiro[(2-(4-fluorobenzoyl)-3-(4-methoxybenzyloxycarbonyl)cyclopent-3-ene)-1′,6-penicillanate] (**14a**) and (1′*R*,2′*R*)-benzhydryl spiro[(2-(4-fluorobenzoyl)-5-(4-methoxybenzyloxycarbonyl)cyclopent-4-ene)-1′,6-penicillanate] (**15a**).

Obtained from allene **8i** (51 mg, 0.250 mmol) and 6-alkylidenepenicillanate **11a** (125 mg, 0.250 mmol) as described in the general procedure (reaction time: 24 h). Purification of the crude product by flash chromatography (hexane/ethyl acetate, 4:1) gave, in order of elution, **14a** as a colorless solid (64 mg, 0.091 mmol, 36%) and **15a** as a colorless solid (69 mg, 0.097 mmol, 39%).

Compound **14a**: mp 72.9–74.9°C; ${\left[\alpha \right]}_{D}^{25}$ = + 390 (c 0.5 in CH_2_Cl_2_); IR (ATR): $\tilde{\nu }$ = 1,001, 1,104, 1,156, 1,176, 1,203, 1,237, 1,330, 1,515, 1,593, 1,670, 1,710, 1,746, and 1,773 cm^−1^; ^1^H NMR (400 MHz, CDCl_3_): δ = 1.11 (s, 3H), 1.49 (s, 3H), 3.10 (dd, *J* = 18.6 and 3.1 Hz, 1H), 3.51 (dt, *J* = 18.5 and 2.2 Hz, 1H), 3.81 (s, 3H), 4.51 (s, 1H), 4.88 (d, *J* = 11.9 Hz, 1H), 4.93 (d, *J* = 11.9 Hz, 1H), 5.08 (d, *J* = 1.1 Hz, 1H), 5.40 (s, 1H), 6.80–6.82 (m, 2H), 6.91 (s, 1H), 7.01 (t, *J* = 8.6 Hz, 2H), 7.06–7.09 (m, 3H), 7.29–7.34 (m, 10H), and 8.06 (dd, *J* = 5.4 and 8.9 Hz, 2H); ^13^C NMR (100 MHz, CDCl_3_): δ = 26.1, 32.5, 40.8, 52.8, 55.4, 64.3, 66.7, 69.1, 70.7, 71.1, 78.5, 114.0, 115.5 (d, *J* = 21.9 Hz, 2C), 127.1, 127.3, 127.7, 128.3, 128.5, 128.7, 130.5, 132.1 (d, *J* = 9.4 Hz, 2C), 133.9 (d, *J* = 2.7 Hz, 1C), 135.8, 139.2, 139.3, 146.0, 159.9, 163.0, 166.10 (d, *J* = 255.4 Hz, 1C), 167.0, 176.4, and 199.6; ^19^F NMR (376 MHz, CDCl_3_): δ = -104.56 (s, 1F); HRMS (ESI-TOF) m/z: [M + NH_4_]^+^ calculated C_41_H_40_FN_2_O_7_S 723.2535; found 723.2531.

Compound **15a**: mp 84.6–86.6°C; ${\left[\alpha \right]}_{D}^{25}$ = +350 (c 0.5 in CH_2_Cl_2_); IR (ATR): $\tilde{\nu }$ = 983, 1,028, 1,122, 1,152, 1,210, 1,237, 1,331, 1,457, 1,514, 1,595, 1,676, 1,712, and 1,764 cm^−1^; ^1^H NMR (400 MHz, CDCl_3_): δ = 1.11 (s, 3H), 1.51 (s, 3H), 2.47 (dd, *J* = 18.6 and 2.5 Hz, 1H), 3.17 (ddd, *J* = 18.5, 9.1 and 2.0 Hz, 1H), 3.80 (s, 3H), 4.49 (d, *J* = 8.4 Hz, 1H), 4.54 (s, 1H), 5.13 (d, *J* = 12.0 Hz, 1H), 5.17 (d, *J* = 12.0 Hz, 1H), 6.26 (s, 1H), 6.81–6.89 (m, 3H), 6.95 (s, 1H), 7.16 (t, *J* = 8.6 Hz, 1H), 7.27–7.37 (m, 8H), 7.40–7.50 (m, 4H), and 7.93–7.98 (m, 2H); ^13^C NMR (100 MHz, CDCl_3_): δ = 25.9, 32.8, 35.9, 49.4, 55.4, 62.7, 66.4, 69.1, 71.0, 74.0, 78.4, 114.1, 116.2 (d, *J* = 21.9 Hz, 2C), 127.5, 127.5, 128.0, 128.2, 128.3, 128.7, 128.7, 130.4, 131.2 (d, *J* = 9.4 Hz, 2C), 131.6 (d, *J* = 2.9 Hz, 1C), 134.5, 139.7, 144.5, 159.8, 162.3, 166.1 (d, *J* = 255.6 Hz, 1C), 166.8, 174.3, and 196.9; ^19^F NMR (376 MHz, CDCl_3_): δ = -104.21 (s, 1F); HRMS (ESI-TOF) m/z: [M + NH_4_]^+^ calculated C_41_H_40_FN_2_O_7_S 723.2535; found 723.2529.

### 4.4 Biological evaluation

#### 4.4.1 Cell lines

TZM-bl cells (AIDS Research and Reference Reagent Program, National Institutes of Health, United States) were cultured in complete growth medium consisting of Dulbecco’s Minimal Essential Medium (DMEM) supplemented with 10% fetal bovine serum (FBS), 100 U/ml of penicillin–streptomycin (Gibco/Invitrogen, United States), 1 mM of sodium pyruvate (Gibco/Invitrogen, United States), 2 mM of L-glutamine (Gibco/Invitrogen, United States), and 1 mM of non-essential amino acids (Gibco/Invitrogen, United States). Cell cultures were maintained at 37°C in 5% CO_2_.

### 4.5 Viruses and titration

The primary isolates of HIV-1 and HIV-2 used in this study were previously described and characterized for coreceptor usage and susceptibility to antiretroviral drugs (Borrego et al., 2011). The HIV-1 SG3.1 is a reference subtype B strain that uses the CXCR4 coreceptor. It was obtained by transfection of HEK293T cells with pSG3.1 plasmid using jetPRIME transfection reagent (Polyplus-transfection SA, Illkirch, France), according to the instructions of the manufacturer. The HIV-2 03PTHCC19 is a primary isolate that uses a CCR5 coreceptor (Borrego et al., 2011). The 50% tissue culture infectious dose (TCID_50_) of each virus was determined in a single-round viral infectivity assay using a luciferase reporter gene assay in TZM-bl cells (Davis et al., 2009; Borrego et al., 2011) and calculated using the statistical method of Reed and Muench.

### 4.6 Cellular viability assays

The *in vitro* cytotoxicity of test compounds was evaluated in TZM-bl cells using alamarBlue cell viability reagent (Life Technologies, United States), according to the instructions of the manufacturer. The cells were cultured in the presence and absence of serial-fold dilutions of the test compounds. Each dilution of each compound was performed in triplicate wells. Medium controls (only growth medium), cell controls (cells without test compound), and cytotoxicity controls (a compound that kills cells) were employed in each assay. The cytotoxicity of each test compound was expressed by the 50% cytotoxic concentration (CC_50_), corresponding to the concentration of compound causing a 50% decrease of cellular viability.

### 4.7 Anti-HIV assays

The antiviral activity of test compounds was determined in a single-round viral infectivity assay using TZM-bl reporter cells, as previously described (Davis et al., 2009; Borrego et al., 2011; Borrego et al., 2013). Briefly, TZM-bl cells were infected with 200 TCID_50_ of HIV-1 or HIV-2 in the presence of serial-fold dilutions of the compounds in the growth medium, supplemented with diethylaminoethyl-dextran (DEAE-dextran). After 48 h of infection, luciferase expression was quantified with Pierce Firefly Luc One-Step Glow Assay Kit (ThermoFisher Scientific, Rockford, United States), according to the instructions of the manufacturer. For each compound dilution, the assay was set up in triplicate wells. Virus controls, cell controls, and inhibitors controls (drugs with a known action against each virus) were employed. Statistical analysis was performed using Prism version 5.01 for Windows (GrahPad Software, San Diego, California, United States, www.graphpad.com), with a level of significance of 5%. IC_50_ and IC_90_ were estimated by the sigmoidal dose-response (variable slope) equation in Prism version 5.01 for Windows (GraphPad Software, United States).

### 4.8 Evaluation of hepatic stage antiplasmodial activity

The inhibitory activity of test compounds on *in vitro* hepatic infection by *P. berghei* was determined by comparing the parasite load in compound- and solvent-treated *P. berghei*-infected Huh7 cells. Infection load was assessed by measurement of luminescence intensity in Huh7 cells infected, with a firefly luciferase-expressing *P. berghei* line, as previously described (Ploemen et al., 2009; António et al., 2017). Briefly, Huh7 cells were seeded in 96-well plates (1.0 × 10^4^ cells per well) the day before drug treatment and infection. The medium was replaced by another medium containing the appropriate concentration of each compound approximately 1 h prior to infection with sporozoites freshly obtained through disruption of salivary glands of infected female *Anopheles stephensi* mosquitoes. Sporozoite addition was followed by centrifugation at 1,800 *g* for 5 min. Parasite infection load was measured 48 h after infection by a bioluminescence assay (Biotium). Cell confluence, a surrogate measure of compound toxicity to Huh7 cells, was assessed by fluorescence measurements using the alamarBlue assay, according to the manufacturer’s instructions. IC_50_ values were determined by evaluating the activity of the tested compounds at seven different concentrations ranging from 0.01 to 1, 5, or 10 μM. Specifically, non-linear regression analysis was employed to fit the normalized results of the dose-response curves, and IC_50_ values were determined using GraphPad Prism 8.0 (GraphPad Software, La Jolla, California, United States).

### 4.9 Evaluation of blood stage antiplasmodial activity

Ring-stage synchronized cultures of *P. falciparum* strain NF54 at 2.5% hematocrit and at approximately 1% parasitemia were incubated with test compounds or dimethyl sulfoxide (DMSO, vehicle control) in 96-well plates for 48 h at 37°C in a 5% CO_2_ and 5% O_2_ atmosphere. Stock solutions of chloroquine (positive control) and compounds **9i**, **12a**, and **14a** were prepared in DMSO. Working solutions were prepared from the stock solutions in complete malaria culture medium (CMCM), which consists of RPMI 1640 supplemented with 25 mM HEPES, 2.4 mM L-glutamine, 50 μg/ml gentamicin, 0.5% w/v Albumax, 11 mM glucose, 1.47 mM hypoxanthine, and 37.3 mM NaHCO_3_. For each measurement, 5 μl of the culture (approximately 800,000 cells) were stained with the DNA-specific dye SYBR green I. After 20 min of incubation in the dark, the stained sample was analyzed by flow cytometry. Approximately 100,000 events were analyzed in each flow cytometry measurement. Two independent experiments were performed, and all samples were analyzed in triplicate.

## Data Availability

The original contributions presented in the study are included in the article/Supplementary Material, further inquiries can be directed to the corresponding authors.
