# Supplementary material for: Unveiling a family of spiro-β-lactams with anti-HIV and antiplasmodial activity via phosphine-catalyzed [3+2] annulation of 6-alkylidene-penicillanates and allenoates
Source: Front Chem. 2022 Oct 7;10:1017250. doi: 10.3389/fchem.2022.1017250 (PMC9585939; doi:10.3389/fchem.2022.1017250)
Supplement: Supplementary file 2 [file DataSheet1.docx]

**Unveiling a family of spiro-β-lactams with anti-HIV and anti-plasmodial activity via phosphine-catalyzed [3+2] annulation of 6-alkylidene-penicillanates and allenoates**

Américo J. S. Alves,^a,#^ Nuno G. Alves,^a,#^ Inês Bártolo,^b^ Diana Fontinha,^c^ Soraia Caetano,^c^ Miguel Prudêncio,^c^ Nuno Taveira,^b,d,^* Teresa M. V. D. Pinho e Melo^a,^*

^a^ University of Coimbra, Coimbra Chemistry Centre-Institute of Molecular Sciences and Department of Chemistry, 3004-535 Coimbra, Portugal.

^b^ Instituto de Investigação do Medicamento (iMed.ULisboa), Faculdade de Farmácia, Universidade de Lisboa, Av. Prof. Gama Pinto, 1649-003 Lisboa, Portugal

^c^ Instituto de Medicina Molecular João Lobo Antunes, Faculdade de Medicina, Universidade de Lisboa, Avenida Professor Egas Moniz, 1649-028 Lisboa, Portugal

^d^ Centro de Investigação Interdisciplinar Egas Moniz (CiiEM), Instituto Universitário Egas Moniz (IUEM), Caparica, Portugal

*^#^ These authors contributed equally to this work.*

**SUPPLEMENTARY DATA**

**Table of Contents**

Synthesis of allenoates **8** S2

Synthesis of spiro-b-lactams **9**, **10**, **12**, **13**, **14** and **15** S8

Copies of ^1^H, ^13^C NMR Spectra for New Compounds S37

Preliminary screen of *in vitro* activity of compounds against the hepatic

stage of *P. berghei* infection S130

qHNMR of selected compounds S131

**Scheme S1.** Synthesis of allenoates **8**

**General procedure for the synthesis of monosubstituted allenoates**

The monosubstituted allenoates were obtained through a synthetic procedure, previously reported in the literature.^1^

Bromoacetyl bromide (0.252 mL, 2.90 mmol) was added dropwise to a solution of an alcohol (2.90 mmol) and pyridine (0.232 mL, 2.90 mmol) in DCM (10 mL) at 0 ºC, forming a white suspension. The suspension was stirred for 20 min at 0 ºC and then for an additional 30 min at 25 ºC, after which distilled water (15 mL) was added to the reaction mixture. The organic layer was separated, and the aqueous layer was then extracted with DCM (2 x 15 mL). The combined organic layers were washed with brine (15 mL) dried over with MgSO_4_ and concentrated to give the respective α-bromoacetate as an oil, which was used directly without purification for the next reaction step.

The α-bromoacetate obtained in the previous step was added dropwise to a solution of triphenylphospine (0.754 g, 2.9 mmol) in toluene (20 mL), and was left stirring overnight. The resulting precipitated was filtered, washed sequentially with toluene and hexane, and then dissolved in distilled water (20 mL). NaOH (2 M) was added to keep the pH > 7 and the mixture was left stirring. After 30 min, DCM (20 mL) was added. The organic layer was then separated, washed with brine (15 mL), dried over with MgSO_4_ and the filtrate was concentrated affording the desired phosphorus ylide, which was used directly without further purification for the next step.

The phosphorus ylide was then dissolved in anhydrous DCM (5 mL) in a two-neck round-bottled flask, and NEt_3_ (0.405 mL, 2.90 mmol) was added dropwise to the solution. After stirring for 15 min, a previously prepared solution of acetyl chloride (0.212 mL, 2.90 mmol) in anhydrous DCM (5 mL) was added dropwise over 30 min. The reaction mixture was left stirring for 12 h under nitrogen. A precipitate was formed which was filtered and discarded, and the solvent was carefully evaporated under reduced pressure. The desired allenoate was purified by flash chromatography [ethyl acetate/hexane], being obtained as an oil or a low melting point solid.

Phenyl 2,3-butadienoate (**8a**)

Allenoate **8a**^2^ was obtained as described in the general procedure from the corresponding alcohol (phenol) and purified by flash chromatography [ethyl acetate/hexane (1:2)], being obtained as a low melting point white solid (0.274 g, 1.711 mmol, 59%). ^1^H NMR (400 MHz, CDCl_3_) δ = 5.34 (d, *J* = 6.8 Hz, 2H), 5.82 (t, *J* = 6.4 Hz, 1H), 7.12-7.14 (m, 2H), 7.22-7.26 (m, 1H), 7.37-7.41 (m, 2H); ^13^C NMR (100 MHz, CDCl_3_) δ = 79.9, 87.9, 121.7, 126.0, 129.6, 150.9, 164.3, 216.8.

4-*tert*-Butylphenyl 2,3-butadienoate (**8b**)

Allenoate **8b**^3^ was obtained as described in the general procedure from the corresponding alcohol (*tert*-butylphenol) and purified by flash chromatography [ethyl acetate/hexane (1:8)], being obtained as a white solid (0.451 g, 2.088 mmol, 72%). ^1^H NMR (400 MHz, CDCl_3_) δ = 1.32 (s, 9H), 5.32 (d, *J* = 6.4 Hz, 2H), 5.82 (t, *J* = 6.4 Hz, 1H), 7.02-7.06 (m, 2H), 7.37-7.40 (m, 2H); ^13^C NMR (100 MHz, CDCl_3_) δ = 31.6, 34.6, 79.9, 87.9, 120.9, 126.5, 148.6, 148.8, 164.5, 216.7; HRMS (ESI) m/z: Calcd for C_14_H_16_NaO_2_ [M+Na]^+^ 239.1043; Found 239.1037.

3-Metoxyphenyl 2,3-butadienoate (**8c**)

Allenoate **8c**^4^ was obtained as described in the general procedure from the corresponding alcohol (3-methoxyphenol) and purified by flash chromatography [ethyl acetate/hexane (1:4)], being obtained as a low melting point white solid (0.337 g, 1.769 mmol, 61%). ^1^H NMR (400 MHz, CDCl_3_) δ = 3.80 (s, 3H), 5.34 (d, *J* = 6.4 Hz, 2H), 5.82 (t, *J* = 6.4 Hz, 1H), 6.68-6.80 (m, 3H), 7.25-7.29 (m, 1H); ^13^C NMR (100 MHz, CDCl_3_) δ = 55.6, 80.00, 87.8, 107.6, 112.00, 113.9, 129.9, 151.9, 160.6, 164.2, 216.8; HRMS (ESI) m/z: Calcd for C_11_H_10_NaO_3_ [M+Na]^+^ 213.0522; Found 213.0518.

2-Naphthyl 2,3-butadienoate (**8d**)

Allenoate **8d**^2^ was obtained as described in the general procedure from the corresponding alcohol (2-naphthol) and purified by flash chromatography [ethyl acetate/hexane (1:8)], being obtained as a white solid (0.402 g, 1.914 mmol, 66%). ^1^H NMR (400 MHz, CDCl_3_) δ = 5.37 (d, *J* = 6.8 Hz, 2H), 5.89 (t, *J* = 6.4 Hz, 1H), 7.28 (dd, *J* = 8.8 and 2.4 Hz, 1H), 7.45-7.52 (m, 2H), 7.61 (d, *J* = 2.4 Hz, 1H), 7.80-7.87 (m, 3H); ^13^C NMR (100 MHz, CDCl_3_) δ =80.0, 87.9, 118.7, 121.2, 125.9, 126.7, 127.8, 127.9, 129.5, 131.6, 133.9, 148.6, 164.5, 216.9.

Isopentyl 2,3-butadienoate (**8f**)

Allenoate **8f** was obtained as described in the general procedure from the corresponding alcohol (isopentanol) and purified by flash chromatography [ethyl acetate/hexane (1:4)], being obtained as a colourless oil (0.322 g, 2.088 mmol, 72%). ^1^H NMR (400 MHz, CDCl_3_) δ = 0.92 (d, *J* = 6.4 Hz, 6H), 1.55 (q, *J* = 6.8, 2H), 1.69 (m, 1H), 4.18 (t, *J* = 6.8 Hz, 2H), 5.21 (d, *J* = 6.4 Hz, 2H), 5.63 (t, *J* = 6.4 Hz, 1H); ^13^C NMR (100 MHz, CDCl_3_) δ = 22.6, 25.2, 37.4, 63.9, 79.4, 88.2, 166.0, 215.9; HRMS (ESI) m/z: Calcd for C_9_H_14_NaO_2_ [M+Na]^+^ 177.0886; Found 177.0884.

Benzhydryl 2,3-butadienoate (**8g**)

Allenoate **8g** was obtained as described in the general procedure from the corresponding alcohol (benzhydryl alcohol) and purified by flash chromatography [ethyl acetate/hexane (1:4)], being obtained as a low melting point colourless solid (0.558 g, 2.233 mmol, 77%). ^1^H NMR (400 MHz, CDCl_3_) δ = 5.27 (d, *J* = 6.8 Hz, 2H), 5.74 (t, *J* = 6.4 Hz, 1H), 6.93 (s, 1H), 7.27-7.38 (m, 10H); ^13^C NMR (100 MHz, CDCl_3_) δ = 79.6, 88.2, 127.2, 128.1, 128.6, 140.3, 164.9, 216.4; HRMS (ESI) m/z: Calcd for C_17_H_14_NaO_2_ [M+Na]^+^ 273.0886; Found 273.0881.

4-Nitrobenzyl 2,3-butadienoate (**8h**)

Allenoate **8h** was obtained as described in the general procedure from the corresponding alcohol (4-nitrobenzyl alcohol) and purified by flash chromatography [ethyl acetate/hexane (1:3)], being obtained as a colourless solid (0.494 g, 2.253 mmol, 77%). ^1^H NMR (400 MHz, CDCl_3_) δ = 5.28 (s, 2H), 5.28 (d, *J* = 6.4 Hz, 2H), 5.72 (t, *J* = 6.4 Hz, 1H), 7.52-7.54 (m, 2H), 8.21-8.24 (m, 2H); ^13^C NMR (100 MHz, CDCl_3_) δ = 65.2, 79.8, 87.6, 124.0, 128.4, 143.3, 147.9, 165.4, 216.5; HRMS (ESI) m/z: Calcd for C_11_H_8_NO_4_ [M-H] 218.0459; Found 218.0456.

4-Methoxybenzyl 2,3-butadienoate (**8i**)

Allenoate **8i** was obtained as described in the general procedure from the corresponding alcohol (4-methoxybenzyl alcohol) and purified by flash chromatography [ethyl acetate/hexane (1:4)], being obtained as a colourless oil (0.385 g, 1.885 mmol, 65%). ^1^H NMR (400 MHz, CDCl_3_) δ = 3.81 (s, 3H), 5.13 (s, 2H), 5.22 (d, *J* = 6.4 Hz, 2H), 5.66 (t, *J* = 6.4 Hz, 1H), 6.87-6.91 (m, 2H), 7.30-7.33 (m, 2H); ^13^C NMR (100 MHz, CDCl_3_) δ = 55.4, 66.6, 79.5, 88.1, 114.1, 128.1, 130.2, 159.8, 165.8, 216.1; HRMS (ESI) m/z: Calcd for C_12_H_12_NaO_3_ [M+Na]^+^ 227.0679; Found 227.0676.

4-Methylbenzyl 2,3-butadienoate (**8j**) and 4-methylbenzyl 2-butinoate (**8j’**)

Obtained as described in the general procedure from the corresponding alcohol (4-methylbenzyl alcohol). After flash chromatography [ethyl acetate/hexane (1:4)], a mixture of products **8j** and **8j’** (1:0.55) was obtained as a colourless oil (0.300 g, 1.595 mmol, 55%).

Compound **8j**: ^1^H NMR (400 MHz, CDCl_3_) δ = 2.36 (s, 3H), 5.15 (s, 2H), 5.23 (d, *J* = 6.8 Hz, 2H), 5.67 (t, *J* = 6.4 Hz, 1H), 7.16-7.18 (m, 2H), 7.25-7.28 (m, 2H); HRMS (ESI) m/z: Calcd for C_12_H_13_O_2_ [M+H]^+^ 189.0910; Found 189.0908.

Compound **8j’**: ^1^H NMR (400 MHz, CDCl_3_) δ = 2.09 (s, 3H), 2.36 (s, 3H), 5.07 (s, 2H), 7.16-7.18 (m, 2H), 7.25-7.28 (m, 2H); HRMS (ESI) m/z: Calcd for C_12_H_13_O_2_ [M+H]^+^ 189.0910; Found 189.0908.

4-Chlorobenzyl 2,3-butadienoate (**8k**) and 4-chlorobenzyl 2-butinoate (**8k’**)

Obtained as described in the general procedure from the corresponding alcohol (4-chlorobenzyl alcohol). After flash chromatography [ethyl acetate/hexane (1:4)], a mixture of products **8k** and **8k’** (1:0.35) was obtained as a colourless oil (0.356 g, 1.711 mmol, 59%).

Compound **8k**: ^1^H NMR (400 MHz, CDCl_3_) δ = 5.15 (s, 2H), 5.24 (d, *J* = 6.8 Hz, 2H), 5.67 (t, *J* = 6.4 Hz, 1H), 7.28-7.34 (m, 4H); HRMS (ESI) m/z: Calcd for C_11_H_10_O_2_Cl [M+H]^+^ 209.0364; Found 209.0362.

Compound **8k’**: ^1^H NMR (400 MHz, CDCl_3_) δ = 2.10 (s, 3H), 5.06 (s, 2H), 7.28-7.34 (m, 4H); HRMS (ESI) m/z: Calcd for C_11_ClH_10_O_2_ [M+H]^+^ 209.0364; Found 209.0362.

4-Fluorobenzyl 2,3-butadienoate (**8l**) and 4-fluorobenzyl 2-butinoate (**8l’**)

Obtained as described in the general procedure from the corresponding alcohol (4-fluorobenzyl alcohol). After flash chromatography [ethyl acetate/hexane (1:4)], a mixture of products (**8l**) and (**8l’**) (1:0.35) was obtained as a colourless oil (0.340 g, 1.769 mmol, 61%).

Compound **8l**: ^1^H NMR (400 MHz, CDCl_3_) δ = 5.15 (s, 2H), 5.24 (d, *J* = 6.4 Hz, 2H), 5.67 (t, *J* = 6.8 Hz, 1H), 7.02-7.06 (m, 2H), 7.32-7.37 (m, 2H); HRMS (ESI) m/z: Calcd for C_11_H_10_O_2_F [M+H]^+^ 193.0659; Found 193.0658.

Compound **8l’**: ^1^H NMR (400 MHz, CDCl_3_) δ = 2.09 (s, 3H), 5.07 (s, 2H), 7.02-7.06 (m, 2H), 7.32-7.37 (m, 2H); HRMS (ESI) m/z: Calcd for C_11_FH_10_O_2_ [M+H]^+^ 193.0659; Found 193.0658.

Propargyl 2,3-butadienoate (**8m**)

Allenoate **8m**^2^ was obtained as described in the general procedure from the corresponding alcohol (propargyl alcohol) and purified by flash chromatography [ethyl acetate/hexane (1:2)], being obtained as a low melting point white solid (0.296 g, 2.465 mmol, 85%). ^1^H NMR (CDCl_3_, 400 MHz) δ = 2.48 (t, *J* = 2.4 Hz, 1H), 4.74 (d, *J* = 2.4 Hz, 2H), 5.26 (d, *J* = 6.4 Hz, 2H), 5.67 (t, *J* = 6.4 Hz, 1H); ^13^C NMR (100 MHz, CDCl_3_) δ = 52.5, 75.1, 77.6, 79.8, 87.4, 165.0, 216.4.

(*E*)-Cinnamyl 2,3-butadienoate (**8n**) and (*E*)-cinnamyl 2-butinoate (**8n’**)

Obtained as described in the general procedure from the corresponding alcohol ((*E*)-cinnamyl alcohol). After flash chromatography [ethyl acetate/hexane (1:4)], a mixture of products **8n** and **8n’** (1:0.25) was obtained as a yellow oil (0.383 g, 1.914 mmol, 66%).

Compound **8n**: ^1^H NMR (400 MHz, CDCl_3_) δ = 4.82 (dd, *J* = 6.4 and 1.2 Hz, 2H), 5.25 (d, *J* = 6.4 Hz, 2H), 5.69 (t, *J* = 6.8 Hz, 1H), 6.27-6.34 (m, 1H), 6.67 (d, *J* = 16.0 Hz, 1H), 7.24-7.41 (m, 5H); HRMS (ESI) m/z: Calcd for C_13_H_13_O_2_ [M+H]^+^ 201.0910; Found 201.0909.

Compound **8n’**: ^1^H NMR (400 MHz, CDCl_3_) δ = 2.11 (s, 3H), 4.73 (dd, *J* = 6.4 and 1.2 Hz, 2H), 6.27-6.34 (m, 1H), 6.66 (d, *J* = 16.0 Hz, 1H), 7.24-7.41 (m, 5H); HRMS (ESI) m/z: Calcd for C_13_H_13_O_2_ [M+H]^+^ 201.0910; Found 201.0909.

1-Phenylpropan-2-yl 2,3-butadienoate (**8o**) and 1-Phenylpropan-2-yl 2-butinoate (**8o’**)

Obtained as described in the general procedure from the corresponding alcohol (2-phenylpropanol). After flash chromatography [ethyl acetate/hexane (1:4)], a mixture of products (**8o**) and (**8o’**) (1:0.75) was obtained as a yellow oil (0.269 g, 1.334 mmol, 46%).

Compound **8o**: ^1^H NMR (400 MHz, CDCl_3_) δ = 1.32 (t, *J* = 6.8 Hz, 3H), 3.11-3.14 (m, 1H), 4.14-4.28 (m, 2H), 5.20 (d, *J* = 6.4 Hz, 2H), 5.61 (t, *J* = 6.8 Hz, 1H), 7.21-7.33 (m, 5H); HRMS (ESI) m/z: Calcd for C_13_H_14_NaO_2_ [M+Na]^+^ 225.0886; Found 225.0882.

Compound **8o’**: ^1^H NMR (400 MHz, CDCl_3_) δ = 1.32 (t, *J* = 6.8 Hz, 3H), 2.02 (s, 3H), 3.11-3.14 (m, 1H), 4.14-4.28 (m, 2H), 7.21-7.33 (m, 5H); HRMS (ESI) m/z: Calcd for C_13_H_14_NaO_2_ [M+Na]^+^ 225.0886; Found 225.0882.

**General procedure for the phosphine-catalyzed [3+2] annulation of allenoates with 6-alkylidenepenicillanates**

The general procedure for the synthesis of spirocyclopentenyl-β-lactams through phosphine-catalyzed [3+2] annulation of allenoates with 6-alkylidenepenicillanates is described in the literature.^5^

To a mixture of the appropriate 6-alkylidenepenicillanate (1 equiv.) and PPh_3_ (20 mol%) in toluene (2-3 mL), a solution of allene (1 equiv.) in toluene (1-2 mL) was added. The reaction mixture was stirred at room temperature under nitrogen for the time indicated in each case, being monitored through TLC. Upon completion, the solvent was removed under reduced pressure and the crude product was purified by flash chromatography.

(1’*R*,2’*R*)-Benzhydryl spiro[(2-benzoyl-3-(4-*tert*-butylphenoxycarbonyl)cyclopent-3-ene)-1’,6-penicillanate] (**9b**) and (1’*R*,2’*R*)-Benzhydryl spiro[(2-benzoyl-5-(4-*tert*-butylphenoxycarbonyl)cyclopent-4-ene)-1’,6-penicillanate] (**10b**)

Obtained from 4-*tert*-butylphenyl 2,3-butadienoate (**8b**) (0.045 mg, 0.21 mmol) and 6-alkylidenepenicillanate **7** (0.100 mg, 0.21 mmol) as described in the general procedure. The reaction mixture was stirred for 6 h. Purification of the crude product by flash chromatography [(ethyl acetate/hexane (1:4)] gave, in order of elution, **9b** as a colourless solid (0.052 g, 0.074 mmol, 36%) and **10b** as a colourless solid (0.066 g, 0.094 mmol, 46%).

Compound **9b**: mp 206.7-208.9 ^o^C; ${[\alpha]}_{D}^{25}$= + 320 (*c* 0.25 in CH_2_Cl_2_); IR (ATR): ν = 1770, 1734, 1671, 1626 cm ^–1^; ^1^H NMR (400 MHz, CDCl_3_) δ = 1.13 (s, 3H), 1.26 (s, 9H), 1.53 (s, 3H), 3.20 (dd, *J* = 18.8 and 3.2 Hz, 1H), 3.63 (dt, *J* = 18.8 and 2.0 Hz, 1H), 4.56 (s, 1H), 5.27 (d, *J* = 1.2 Hz, 1H), 5.48 (s, 1H), 6.73-6.77 (m, 2H), 6.93 (s, 1H), 7.25-7.36 (m, 13H), 7.42-7.46 (m, 2H), 7.53-7.57 (m, 1H), 8.11-8.133 (m, 2H); ^13^C NMR (100 MHz, CDCl_3_) δ = 26.1, 31.5, 32.5, 34.6, 41.0, 53.0, 64.3, 69.1, 70.8, 71.2, 78.5, 120.8, 126.4, 127.1, 127.7, 128.4, 128.6, 128.6, 128.8, 129.5, 133.7, 135.7, 137.5, 139.2, 139.3, 147.4, 148.0, 148.8, 161.6, 167.0, 176.4, 201.2; HRMS (ESI) m/z: Calcd for C_43_H_41_NNaO_6_S [M+Na]^+^ 722.2547; Found 722.2536.

Compound **10b**: mp 91.8-93.5 ^o^C; ${[\alpha]}_{D}^{25}$= + 390 (*c* 0.5 in CH_2_Cl_2_); IR (ATR): ν = 1768, 1735, 1676, 1448 cm ^–1^; ^1^H NMR (400 MHz, CDCl_3_) δ = 1.11 (s, 3H), 1.35 (s, 9H), 1.52 (s, 3H), 2.61 (dd, *J* = 18.8 and 2.4 Hz, 1H), 3.29 (ddd, *J* = 18.8, 9.2 and 2.0 Hz, 1H), 4.55 (s, 1H), 4.62 (d, *J* = 8.4 Hz, 1H), 6.34 (s, 1H), 6.89 (s, 1H), 7.08-7.14 (m, 6H), 7.25-7.29 (m, 3H), 7.33-7.36 (m, 4H), 7.39-7.41 (m, 2H), 7.50-7.54 (m, 2H), 7.61-7.64 (m, 1H), 7.97-7.99 (m, 2H); ^13^C NMR (100 MHz, CDCl_3_) δ = 25.9, 31.6, 33.0, 34.7, 36.2, 49.4, 62.8, 69.2, 71.1, 74.1, 78.3, 121.4, 126.3, 127.2, 127.6, 127.9, 128.2, 128.6, 128.6, 129.1, 133.8, 134.1, 135.1, 139.5, 139.7, 146.0, 148.3, 148.9, 161.3, 166.8, 174.2, 198.5; HRMS (ESI) m/z: Calcd for C_43_H_41_NNaO_6_S [M+Na]^+^ 722.2547; Found 722.2537.

(1’*R*,2’*R*)-Benzhydryl spiro[(2-benzoyl-3-(3-methoxyphenoxycarbonyl)cyclopent-3-ene)-1’,6-penicillanate] (**9c**) and (1’*R*,2’*R*)-Benzhydryl spiro[(2-benzoyl-5-(3-methoxyphenoxycarbonyl)cyclopent-4-ene)-1’,6-penicillanate] (**10c**)

Obtained from 3-methoxyphenyl 2,3-butadienoate (**8c**) (0.039 mg, 0.21 mmol) and 6-alkylidenepenicillanate **7** (0.100 mg, 0.21 mmol) as described in the general procedure. The reaction mixture was stirred for 3 h. Purification of the crude product by flash chromatography [(ethyl acetate/hexane (1:4)] gave, in order of elution, **9c** as a colourless solid (0.051 g, 0.076 mmol, 37%) and **10c** as a colourless solid (0.053 g, 0.079 mmol, 38%).

Compound **9c**: mp 67.8-69.7 ^o^C; ${[\alpha]}_{D}^{25}$= + 240 (*c* 0.25 in CH_2_Cl_2_); IR (ATR): ν = 1773, 1735, 1669, 1591, 1490 cm^–1^; ^1^H NMR (400 MHz, CDCl_3_) δ = 1.13 (s, 3H), 1.54. (s, 3H), 3.20 (dd, *J* = 18.8 and 3.2 Hz, 1H), 3.63 (dt, *J* = 18.8 and 2.4 Hz, 1H), 3.71 (s, 3H), 4.56 (s, 1H), 5.26 (d, *J* = 1.2 Hz, 1H), 5.48 (s, 1H), 6.34-6.35 (m, 1H), 6.42-6.44 (m, 1H), 6.69-6.72 (m, 1H), 6.93 (s, 1H), 7.14-7.18 (m, 1H), 7.29-7.36 (m, 11H), 7.42-7.45 (m, 2H), 7.53-7.57 (m, 1H), 8.10-8.13 (m, 2H); ^13^C NMR (100 MHz, CDCl_3_) δ = 26.1, 29.8, 32.6, 40.9, 53.0, 55.5, 64.3, 69.1, 70.8, 71.2, 78.5, 107.3, 112.2, 113.7, 127.1, 127.7, 128.4, 128.6, 128.6, 128.8, 129.5, 129.8, 133.7, 139.2, 147.7, 151.2, 160.5, 161.3, 167.0, 176.4, 201.2; HRMS (ESI) m/z: Calcd for C_40_H_35_NNaO_7_S [M+Na]^+^ 696.2026; Found 696.2018.

Compound **10c**: mp 74.2-76.7 ^o^C; ${[\alpha]}_{D}^{25}$= + 360 (*c* 0.375 in CH_2_Cl_2_); IR (ATR): ν = 1763, 1735, 1676, 1592, 1490 cm ^–1^; ^1^H NMR (400 MHz, CDCl_3_) δ = 1.11 (s, 3H), 1.53 (s, 3H), 2.62 (dd, *J* = 18.8 and 2.4 Hz, 1H), 3.29 (ddd, *J* = 18.8, 9.2 and 2.0 Hz, 1H), 3.80 (s, 3H), 4.54 (s, 1H), 4.62 (d, *J* = 8.4 Hz, 1H), 6.34 (s, 1H), 6.76-6.83 (m, 3H), 6.89 (s, 1H), 7.12-7.17 (m, 4H), 7.24-7.31 (m, 4H), 7.34-7.37 (m, 4H), 7.50-7.54 (m, 2H), 7.61-7.64 (m, 1H), 7.96-7.98 (m, 2H); ^13^C NMR (100 MHz, CDCl_3_) δ = 25.9, 33.0, 36.2, 49.5, 55.6, 62.8, 69.2, 71.1, 74.1, 78.4, 107.7, 112.3, 114.2, 127.3, 127.5, 128.0, 128.2, 128.6, 129.1, 129.8, 133.8, 134.0, 135.1, 139.4, 139.6, 146.3, 151.6, 160.6, 160.9, 166.7, 174.2, 198.5; HRMS (ESI) m/z: Calcd for C_40_H_35_NNaO_7_S [M+Na]^+^ 696.2026; Found 696.2014.

(1’*R*,2’*R*)-Benzhydryl spiro[(2-benzoyl-3-(2-naphthoxycarbonyl)cyclopent-3-ene)-1’,6-penicillanate] (**9d**) and (1’*R*,2’*R*)-Benzhydryl spiro[(2-benzoyl-5-(2-naphthoxycarbonyl)cyclopent-4-ene)-1’,6-penicillanate] (**10d**)

Obtained from 2-naphthyl 2,3-butadienoate (**8d**) (0.044 mg, 0.21 mmol) and 6-alkylidenepenicillanate **7** (0.100 mg, 0.21 mmol) as described in the general procedure. The reaction mixture was stirred for 4 h. Purification of the crude product by flash chromatography [(dichloromethane/ethyl acetate/hexane (1:0,5:4)] gave, in order of elution, **9d** as a colourless solid (0.017 g, 0.024 mmol, 12%) and **10d** as a colourless solid (0.051 g, 0.073 mmol, 35%).

Compound **9d**: mp > 206.5 ^o^C (with decomposition); ${[\alpha]}_{D}^{25}$= + 300 (*c* 0.25 in CH_2_Cl_2_); IR (ATR): ν = 1767, 1727, 1663, 1628 cm ^–1^; ^1^H NMR (400 MHz, CDCl_3_) δ = 1.14 (s, 3H), 1.55 (s, 3H), 3.23 (dd, *J* = 18.8 and 3.2 Hz, 1H), 3.66 (dt, *J* = 18.8 and 2.4 Hz, 1H), 4.57 (s, 1H), 5.31 (d, *J* = 1.2 Hz, 1H), 5.50 (s, 1H), 6.94 (s, 1H), 6.94-6.97 (m, 1H), 7.23 (d, *J* = 2.0 Hz, 1H), 7.30-7.36 (m, 11H), 7.42-7.46 (m, 4H), 7.53-7.56 (m, 1H), 7.73-7.79 (m, 3H), 8.13-8.15 (m, 2H); ^13^C NMR (100 MHz, CDCl_3_) δ = 26.1, 32.6, 41.0, 53.1, 64.3, 69.1, 70.8, 71.2, 78.5, 118.6, 121.0, 125.9, 126.6, 127.2, 127.7, 127.8, 127.8, 128.4, 128.6, 128.6, 128.8, 129.5, 131.6, 133.7, 135.5, 137.5, 139.2, 139.3, 147.8, 161.6, 167.0, 176.4, 201.2; HRMS (ESI) m/z: Calcd for C_43_H_35_NNaO_6_S [M+Na]^+^ 716.2077; Found 716.2066.

Compound **10d**: mp 195.6-198.0 ^o^C; ${[\alpha]}_{D}^{25}$= + 400 (*c* 0.375 in CH_2_Cl_2_); IR (ATR): ν = 1777, 1739, 1724, 1681 cm ^–1^; ^1^H NMR (400 MHz, CDCl_3_) δ = 1.12 (s, 3H), 1.54 (s, 3H), 2.65 (dd, *J* = 18.8 and 2.4 Hz, 1H), 3.32 (ddd, *J* = 18.8, 9.2 and 2.0 Hz, 1H), 4.56 (s, 1H), 4.64 (d, *J* = 8.4 Hz, 1H), 6.38 (s, 1H), 6.87 (s, 1H), 6.97-7.07 (m, 3H), 7.19-7.25 (m, 4H), 7.32-7.34 (m, 5H), 7.47-7.55 (m, 4H), 7.61-7.65 (m, 1H), 7.70 (d, *J* = 2.0 Hz, 1H), 7.81-7.88 (m, 3H), 7.98-8.00 (m, 2H); ^13^C NMR (100 MHz, CDCl_3_) δ = 25.9, 33.1, 36.3, 49.5, 62.8, 69.2, 71.1, 74.2, 78.3, 119.0, 121.4, 125.9, 126.7, 127.3, 127.5, 127.9, 128.0, 128.2, 128.5, 128.6, 128.6, 129.1, 129.4, 131.6, 133.8, 133.9, 134.1, 135.1, 139.4, 139.6, 146.4, 148.3, 161.2, 166.7, 174.2, 198.5; HRMS (ESI) m/z: Calcd for C_43_H_35_NNaO_6_S [M+Na]^+^ 716.2077; Found 716.2068.

(1’*R*,2’*R*)-Benzhydryl spiro[(2-benzoyl-3-pentoxycarbonylcyclopent-3-ene)-1’,6-penicillanate] (**9e**) and (1’*R*,2’*R*)-Benzhydryl spiro[(2-benzoyl-5-pentoxycarbonylcyclopent-4-ene)-1’,6-penicillanate] (**10e**)

Obtained from pentyl 2,3-butadienoate (**8e**) (0.032 mg, 0.21 mmol) and 6-alkylidenepenicillanate **7** (0.100 mg, 0.21 mmol) as described in the general procedure. The reaction mixture was stirred for 6 h. Purification of the crude product by flash chromatography [(ethyl acetate/hexane (1:5)] gave, in order of elution, **9e** as a low melting point yellow solid (0.045 g, 0.071 mmol, 34%) and **10e** as a low melting point yellow solid (0.079 g, 0.124 mmol, 60%).

Compound **9e**: ${[\alpha]}_{D}^{25}$= + 320 (*c* 0.25 in CH_2_Cl_2_); IR (ATR): ν = 1773, 1744, 1710, 1669 cm ^–1^; ^1^H NMR (400 MHz, CDCl_3_) δ = 0.84 (t, *J* = 7.2 Hz, 3H) 1.12 (s, 3H), 1.15-1.26 (m, 4H), 1.36-1.41 (m, 2H), 1.52 (s, 3H), 3.13 (dd, *J* = 18.4 and 3.2 Hz, 1H), 3.54 (dt, *J* = 18.4 and 2.4 Hz, 1H), 3.88-3.96 (m, 2H), 4.54 (s, 1H), 5.16 (d, *J* = 1.2 Hz, 1H), 5.43 (s, 1H), 6.92 (s, 1H), 7.02 (br s, 1H), 7.28-7.35 (m, 10H,), 7.45-7.49 (m, 2H), 7.56-7.60 (m, 1H), 8.09-8.11 (m, 2H); ^13^C NMR (100 MHz, CDCl_3_) δ =14.0, 22.4, 26.1, 28.0, 28.1, 32.5, 40.8, 52.9, 64.2, 65.1, 69.1, 70.7, 71.2, 78.4, 127.1, 127.7, 128.3, 128.4, 128.5, 128.7, 128.7, 129.4, 133.6, 136.3, 137.6, 139.2, 139.3, 145.2, 163.1, 167.0, 176.5, 201.3. HRMS (ESI) m/z: Calcd for C_38_H_39_NNaO_6_S [M+Na]^+^ 660.2390; Found 660.2382.

Compound **10e**: ${[\alpha]}_{D}^{25}$= + 420 (*c* 0.5 in CH_2_Cl_2_); IR (ATR): ν = 1766, 1712, 1676, 1448 cm ^–1^; ^1^H NMR (400 MHz, CDCl_3_) δ = 0.88-0.91 (m, 3H), 1.12 (s, 3H), 1.23-1.38 (m, 4H), 1.52 (s, 3H), 1.62-1.69 (m, 2H), 2.51 (dd, *J* = 18.8 and 2.4 Hz, 1H), 3.19 (ddd, *J* = 18.4, 9.2 and 2.0 Hz, 1H), 4.14-4.19 (m, 2H), 4.55-4.57 (m, 2H), 6.28 (s, 1H), 6.87 (s, 1H), 6.95 (br s, 1H), 7.27-7.36 (m, 6H), 7.41-7.51 (m, 6H), 7.58-7.62 (m, 1H), 7.93-7.96 (m, 2H); ^13^C NMR (100 MHz, CDCl_3_) δ = 14.1, 14.3, 22.5, 22.8, 25.9, 28.2, 28.3, 29.5, 29.8, 29.9, 32.1, 32.9, 35.9, 49.5, 62.7, 65.1, 69.1, 71.2, 74.1, 78.4, 127.4, 127.5, 128.2, 128.2, 128.6, 128.7, 129.1, 133.6, 134.8, 135.2, 139.7, 144.2, 162.9, 166.8, 174.5, 198.4; HRMS (ESI) m/z: Calcd for C_38_H_39_NNaO_6_S [M+Na]^+^ 660.2390; Found 660.2382.

(1’*R*,2’*R*)-Benzhydryl spiro[(2-benzoyl-3-isopentoxycarbonylcyclopent-3-ene)-1’,6-penicillanate] (**9f**) and (1’*R*,2’*R*)-Benzhydryl spiro[(2-benzoyl-5-isopentoxycarbonylcyclopent-4-ene)-1’,6-penicillanate] (**10f**)

Obtained from isopentyl 2,3-butadienoate (**8f**) (0.032 mg, 0.21 mmol) and 6-alkylidenepenicillanate **7** (0.100 mg, 0.21 mmol) as described in the general procedure. The reaction mixture was stirred for 7 h. Purification of the crude product by flash chromatography [(ethyl acetate/hexane (1:4)] gave, in order of elution, **9f** as a low melting point yellow solid (0.040 g, 0.063 mmol, 30%) and **10f** as a low melting point colourless solid (0.044 g, 0.069 mmol, 33%).

Compound **9f**: ${[\alpha]}_{D}^{25}$= + 360 (*c* 0.25 in CH_2_Cl_2_); IR (ATR): ν = 1773, 1744, 1711, 1664 cm ^–1^; ^1^H NMR (400 MHz, CDCl_3_) δ = 0.81 (dd, *J* = 9.2 and 6.4 Hz, 6H), 1.12 (s, 3H), 1.24-1.30 (m, 2H), 1.45-1.50 (m, 1H), 1.52 (s, 3H), 3.11 (dd, *J* = 18.4 and 3.2 Hz, 1H), 3.53 (dt, *J* = 18.4 and 2.4 Hz, 1H), 3.93-3.98 (m, 2H), 4.53 (s, 1H), 5.15 (d, *J* = 1.2 Hz, 1H), 5.43 (s, 1H), 6.92 (s, 1H), 7.00 (br s, 1H), 7.29-7.35 (m, 8H), 7.45-7.51 (m, 3H), 7.56-7.60 (m, 1H), 7.80-7.82 (m, 1H), 8.08-8.10 (m, 2H); ^13^C NMR (100 MHz, CDCl_3_) δ = 22.5, 25.0, 26.1, 32.5, 37.1, 40.8, 52.9, 63.6, 64.2, 60.1, 70.7, 71.2, 78.4, 127.1, 127.7, 128.3, 128.4, 128.5, 128.5, 128.8, 129.4, 130.2, 132.6, 133.6, 136.3, 139.2, 139.3, 145.2, 163.2, 167.0, 176.6, 201.4; HRMS (ESI) m/z: Calcd for C_38_H_39_NNaO_6_S [M+Na]^+^ 660.2390; Found 660.2382.

Compound **10f**: ${[\alpha]}_{D}^{25}$= + 430 (*c* 0.3 in CH_2_Cl_2_); IR (ATR): ν = 1762, 1739, 1717, 1683 cm ^–1^; ^1^H NMR (400 MHz, CDCl_3_) δ = 0.91 (dd, *J* = 6.4 and 4.0 Hz, 6H), 1.11 (s, 3H), 1.52 (s, 3H), 1.53-1.58 (m, 2H), 1.64-1.74 (m, 1H), 2.51 (dd, *J* = 18.8 and 2.4 Hz, 1H), 3.19 (ddd, *J* = 18.4, 9.2 and 2.0 Hz, 1H), 4.21 (t, *J* = 6.88 Hz, 2H), 4.55 (s, 1H), 4.56 (d, *J* = 8.4 Hz, 1H), 6.28 (s, 1H), 6.86 (br s, 1H), 6.95 (s, 1H), 7.27-7.36 (m, 6H), 7.41-7.51 (m, 6H), 7.58-7.62 (m, 1H), 7.93-7.95 (d, *J* = 7.20, 2H); ^13^C NMR (100 MHz, CDCl_3_) δ = 22.6, 22.6, 25.3, 25.9, 33.0, 35.9, 37.3, 49.5, 62.7, 63.6, 69.1, 71.2, 74.1, 78.4, 127.4, 127.5, 128.2, 128.6, 128.7, 128.7, 129.1, 133.6, 134.8, 135.2, 139.7, 144.1, 162.9, 166.8, 174.8, 198.4; HRMS (ESI) m/z: Calcd for C_38_H_39_NNaO_6_S [M+Na]^+^ 660.2390; Found 660.2381.

(1’*R*,2’*R*)-Benzhydryl spiro[(2-benzoyl-3-benzhydryloxycarbonylcyclopent-3-ene)-1’,6-penicillanate] (**9g**) and (1’*R*,2’*R*)-Benzhydryl spiro[(2-benzoyl-5-benzhydryloxycarbonylcyclopent-4-ene)-1’,6-penicillanate] (**10g**)

Obtained from benzhydryl 2,3-butadienoate (**8g**) (0.052 mg, 0.21 mmol) and 6-alkylidenepenicillanate **7** (0.100 mg, 0.21 mmol) as described in the general procedure. The reaction mixture was stirred for 6 h. Purification of the crude product by flash chromatography [(ethyl acetate/hexane (1:5)] gave, in order of elution, **9g** as a colourless solid (0.054 g, 0.074 mmol, 36%) and **10g** as a colourless solid (0.084 g, 0.114 mmol, 55%).

Compound **9g**: mp 83.4-85.2 ^o^C; ${[\alpha]}_{D}^{25}$= + 320 (*c* 0.25 in CH_2_Cl_2_); IR (ATR): ν = 1773, 1744, 1715, 1669 cm ^–1^; ^1^H NMR (400 MHz, CDCl_3_) δ = 1.10 (s, 3H), 1.49 (s, 3H), 3.15 (dd, *J* = 18.8 and 3.2 Hz, 1H), 3.54 (dt, *J* = 18.4 and 2.4 Hz, 1H), 4.52 (s, 1H), 5.19 (d, *J* = 1.2 Hz, 1H), 5.42 (s, 1H), 6.78 (s, 1H), 6.92 (s, 1H), 7.07-7.10 (m, 2H), 7.12-7.15 (m, 3H), 7.24-7.38 (m, 18H), 7.50-7.54 (m, 1H), 8.04-8.06 (m, 2H); ^13^C NMR (100 MHz, CDCl_3_) δ = 26.1, 32.4, 40.9, 52.8, 64.2, 69.1, 70.6, 71.2, 78.4, 127.1, 127.3, 127.7, 128.0, 128.1, 128.3, 128.5, 128.5, 128.6, 128.7, 129.5, 133.7, 136.0, 137.4, 139.2, 139.3, 139.7, 139.7, 146.0, 162.1, 167.0, 176.4, 201.1; HRMS (ESI) m/z: Calcd for C_46_H_38_NO_6_S [M-H] 732.2425; Found 732.2428.

Compound **10g**: mp 95.1-96.8 ^o^C; ${[\alpha]}_{D}^{25}$= + 350 (*c* 0.5 in CH_2_Cl_2_); IR (ATR): ν = 1763, 1718, 1676, 1448 cm ^–1^; ^1^H NMR (400 MHz, CDCl_3_) δ = 1.08 (s, 3H), 1.51 (s, 3H), 2.54 (dd, *J* = 18.8 and 2.4 Hz, 1H), 3.21 (ddd, *J* = 18.8, 9.2 and 2.0 Hz, 1H), 4.54 (s, 1H), 4.58 (d, *J* = 8.4 Hz, 1H), 6.25 (s, 1H), 6.92 (s, 1H), 7.02-7.04 (m, 2H), 7.23-7.34 (m, 12H), 7.39-7.44 (m, 8H), 7.47-7.51 (m, 2H), 7.58-7.62 (m, 1H), 7.93-7.95 (m, 2H); ^13^C NMR (100 MHz, CDCl_3_) δ = 25.9, 32.5, 36.0, 49.6, 62.6, 69.2, 70.9, 73.9, 78.3, 127.1, 127.4, 127.5, 127.6, 128.0, 128.1, 128.2, 128.6, 128.7, 128.7, 128.7, 129.1, 133.7, 134.4, 135.1, 139.6, 139.7, 140.1, 140.2, 145.2, 161.7, 166.7, 174.5, 198.3; HRMS (ESI) m/z: Calcd for C_46_H_39_NNaO_6_S [M+Na]^+^ 756.2390; Found 756.2376.

(1’*R*,2’*R*)-Benzhydryl spiro[(2-benzoyl-3-(4-nitrobenzyloxycarbonyl)cyclopent-3-ene)-1’,6-penicillanate] (**9h**) and (1’*R*,2’*R*)-Benzhydryl spiro[(2-benzoyl-5-(4-nitrobenzyloxycarbonyl)cyclopent-4-ene)-1’,6-penicillanate] (**10h**)

Obtained from 4-nitrobenzyl 2,3-butadienoate (**8h**) (0.068 mg, 0.31 mmol) and 6-alkylidenepenicillanate **7** (0.150 mg, 0.31 mmol) as described in the general procedure. The reaction mixture was stirred for 4 h. Purification of the crude product by flash chromatography [(ethyl acetate/hexane (1:4)] gave, in order of elution, **9h** as a colourless solid (0.083 g, 0.118 mmol, 38%) and **10h** as a colourless solid (0.112 g, 0.160 mmol, 52%).

Compound **9h**: mp 81.2-83.1 ^o^C; ${[\alpha]}_{D}^{25}$= + 340 (*c* 0.25 in CH_2_Cl_2_); IR (ATR): ν = 1770, 1744, 1719, 1669, 1518, 1345, 1330 cm ^–1^; ^1^H NMR (400 MHz, CDCl_3_) δ = 1.12 (s, 3H), 1.51 (s, 3H), 3.14 (dd, *J* = 18.4 and 2.8 Hz, 1H), 3.56 (dt, *J* = 18.8 and 2.4 Hz, 1H), 4.53 (s, 1H), 5.05 (q, *J* = 13.2 Hz, 2H), 5.17 (d, *J* = 1.2 Hz, 1H), 5.43 (s, 1H), 6.92 (s, 1H), 7.12 (br s, 1H), 7.23-7.25 (m, 2H), 7.28-7.35 (m, 10H), 7.39-7.43 (m, 2H), 7.53-7.57 (m, 1H), 8.04-8.06 (m, 2H), 8.09-8.12 (m, 2H); ^13^C NMR (100 MHz, CDCl_3_) δ = 26.1, 32.5, 40.8, 52.8, 64.3, 65.1, 69.1, 70.6, 71.1, 78.5, 123.9, 127.1, 127.7, 128.4, 128.5, 128.6, 128.7, 128.8, 129.4, 133.7, 135.4, 137.4, 139.2, 139.3, 142.6, 146.9, 147.8, 162.5, 166.9, 176.3, 200.9; HRMS (ESI) m/z: Calcd for C_40_H_34_N_2_NaO_8_S [M+Na]^+^ 725.1928; Found 725.1927.

Compound **10h**: mp 83.8-85.2 ^o^C; ${[\alpha]}_{D}^{25}$= + 370 (*c* 0.5 in CH_2_Cl_2_); IR (ATR): ν = 1763, 1718, 1676, 1521, 1345 cm ^–1^; ^1^H NMR (400 MHz, CDCl_3_) δ = 1.11 (s, 3H), 1.53 (s, 3H), 2.53 (dd, *J* = 19.2 and 2.4 Hz, 1H), 3.22 (ddd, *J* = 18.4, 8.8 and 2.0 Hz, 1H), 4.56 (s, 1H), 4.58 (d, *J* = 8.4 Hz, 1H), 5.28 (q, *J* = 9.6 Hz, 2H) 6.23 (s, 1H), 6.93 (s, 1H), 6.97 (br s, 1H), 7.27-7.35 (m, 6H), 7.39-7.44 (m, 4H), 7.50-7.54 (m, 4H), 7.59-7.63 (m, 1H), 7.93-7.95 (m, 2H), 8.18-8.20 (m, 2H); ^13^C NMR (100 MHz, CDCl_3_) δ = 26.0, 32.6, 36.0, 49.6, 62.7, 65.1, 69.2, 71.0, 73.8, 78.5, 124.0, 127.3, 127.6, 128.3, 128.3, 128.6, 128.7, 129.1, 133.8, 134.1, 135.0, 139.5, 139.5, 143.0, 146.0, 147.9, 162.3, 166.8, 174.3, 198.3; HRMS (ESI) m/z: Calcd for C_40_H_34_N_2_NaO_8_S [M+Na]^+^ 725.1928; Found 725.1920.

(1’*R*,2’*R*)-Benzhydryl spiro[(2-benzoyl-3-(4-methoxybenzyloxycarbonyl)cyclopent-3-ene)-1’,6-penicillanate] (**9i**) and (1’*R*,2’*R*)-Benzhydryl spiro[(2-benzoyl-5-(4-methoxybenzyloxycarbonyl)cyclopent-4-ene)-1’,6-penicillanate] (**10i**)

Obtained from 4-methoxybenzyl 2,3-butadienoate (**8i**) (0.064 mg, 0.31 mmol) and 6-alkylidenepenicillanate **7** (0.150 mg, 0.31 mmol) as described in the general procedure. The reaction mixture was stirred for 4 h. Purification of the crude product by flash chromatography [(ethyl acetate/hexane (1:4)] gave, in order of elution, **9i** as a colourless fluffy (0.079 g, 0.115 mmol, 37%) and **10i** as a colourless fluffy (0.114 g, 0.166 mmol, 53%).

Compound **9i**: mp 65.3-67.4 ^o^C; ${[\alpha]}_{D}^{25}$= + 300 (*c* 0.5 in CH_2_Cl_2_); IR (ATR): ν = 1773, 1744, 1713, 1676 cm ^–1^; ^1^H NMR (400 MHz, CDCl_3_) δ = 1.11 (s, 3H), 1.50 (s, 3H), 3.10 (dd, *J* = 18.8 and 3.2 Hz, 1H), 3.53 (dt, *J* = 18.4 and 2.4 Hz, 1H), 3.80 (s. 3H), 4.52 (s, 1H), 4.89 (s, 2H), 5.16 (d, *J* = 1.2 Hz, 1H), 5.42 (s, 1H), 6.80 (d, *J* = 8.8 Hz, 2H), 6.91 (s, 1H), 7.03 (br s, 1H), 7.05-7.07 (m, 2H), 7.29-7.34 (m, 10H), 7.39-7.42 (m, 2H), 7.53-7.57 (m, 1H), 8.05-8.07 (m, 2H); ^13^C NMR (100 MHz, CDCl_3_) δ = 26.1, 32.5, 40.8, 52.8, 55.4, 64.2, 66.5, 69.1, 70.7, 71.2, 78.4, 114.0, 127.1, 127.5, 127.7, 128.3, 128.4, 128.5, 128.7, 129.4, 130.4, 133.6, 136.0, 137.5, 139.2, 139.3, 145.8, 159.8, 162.9, 167.0, 176.5, 201.3; HRMS (ESI) m/z: Calcd for C_41_H_37_NNaO_7_S [M+Na]^+^ 710.2183; Found 710.2172.

Compound **10i**: mp 74.4-76.3 ^o^C; ${[\alpha]}_{D}^{25}$= + 350 (*c* 0.5 in CH_2_Cl_2_); IR (ATR): ν = 1763, 1740, 1713, 1676 cm ^–1^; ^1^H NMR (400 MHz, CDCl_3_) δ = 1.11 (s, 3H), 1.52 (s, 3H), 2.50 (dd, *J* = 18.8 and 2.4 Hz, 1H), 3.18 (ddd, *J* = 18.4, 9.2 and 2.0 Hz, 1H), 3.80 (s, 3H), 4.55 (s, 1H), 4.54-4.56 (m, 1H), 5.15 (q, *J* = 12.0 Hz, 2H) 6.28 (s, 1H), 6.85-6.88 (m, 3H), 6.95 (s, 1H), 7.28-7.36 (m, 8H), 7.42-7.51 (m, 6H), 7.58-7.62 (m, 1H), 7.92-7.94 (m, 2H); ^13^C NMR (100 MHz, CDCl_3_) δ = 25.9, 32.8, 36.0, 49.5, 55.4, 62.7, 66.4, 69.1, 71.1, 73.9, 78.4, 114.1, 127.4, 127.5, 128.0, 128.2, 128.2, 128.6, 128.6, 128.7, 129.1, 130.4, 133.7, 134.5, 135.1, 139.7, 144.7, 159.8, 162.6, 166.8, 174.4, 198.4; HRMS (ESI) m/z: Calcd for C_41_H_37_NNaO_7_S [M+Na]^+^ 710.2183; Found 710.2172.

(1’*R*,2’*R*)-Benzhydryl spiro[(2-benzoyl-3-(4-methylbenzyloxycarbonyl)cyclopent-3-ene)-1’,6-penicillanate] (**9j**) and (1’*R*,2’*R*)-Benzhydryl spiro[(2-benzoyl-5-(4-methylbenzyloxycarbonyl)cyclopent-4-ene)-1’,6-penicillanate] (**10j**)

Obtained from 4-methylbenzyl 2,3-butadienoate (**8j**) (0.059 mg, 0.31 mmol) and 6-alkylidenepenicillanate **7** (0.150 mg, 0.31 mmol) as described in the general procedure. The reaction mixture was stirred for 4 h. Purification of the crude product by flash chromatography [(ethyl acetate/hexane (1:5)] gave, in order of elution, **9j** as a colourless fluffy (0.052 g, 0.077 mmol, 25%) and **10j** as a colourless fluffy (0.096 g, 0.143 mmol, 46%).

Compound **9j**: mp 59.0-60.8 ^o^C; ${[\alpha]}_{D}^{25}$= + 340 (*c* 0.25 in CH_2_Cl_2_); IR (ATR): ν = 1773, 1742, 1710, 1669 cm ^–1^; ^1^H NMR (400 MHz, CDCl_3_) δ = 1.11 (s, 3H), 1.50 (s, 3H), 2.33 (s. 3H), 3.10 (dd, *J* = 18.4 and 3.2 Hz, 1H), 3.53 (dt, *J* = 18.8 and 2.4 Hz, 1H), 4.52 (s, 1H), 4.92 (s, 2H), 5.16 (d, *J* = 1.2 Hz, 1H), 5.42 (s, 1H), 6.91 (s, 1H), 7.01-7.09 (m, 5H), 7.29-7.34 (m, 10H), 7.38-7.42 (m, 2H), 7.53-7.57 (m, 1H), 8.05-8.07 (m, 2H); ^13^C NMR (100 MHz, CDCl_3_) δ = 21.3, 26.1, 32.5, 40.8, 52.9, 64.2, 66.7, 69.1, 70.7, 71.2, 78.4, 127.1, 127.7, 128.3, 128.4, 128.5, 128.7, 128.8, 129.3, 129.4, 132.4, 133.6, 136.0, 137.4, 138.2, 139.2, 139.3, 145.8, 162.9, 167.0, 176.5; HRMS (ESI) m/z: Calcd for C_41_H_37_NNaO_6_S [M+Na]^+^ 694.2234; Found 694.2228.

Compound **10j**: mp 60.7-62.7 ^o^C; ${[\alpha]}_{D}^{25}$= + 375 (*c* 0.4 in CH_2_Cl_2_); IR (ATR): ν = 1763, 1718, 1676, 1448 cm ^–1^; ^1^H NMR (400 MHz, CDCl_3_) δ = 1.11 (s, 3H), 1.52 (s, 3H), 2.34 (s, 3H), 2.50 (dd, *J* = 18.4 and 2.4 Hz, 1H), 3.18 (ddd, *J* = 18.4, 9.2 and 2.0 Hz, 1H), 4.54-4.56 (m, 2H), 5.17 (s, 2H) 6.29 (s, 1H), 6.89 (br s, 1H), 6.95 (s, 1H), 7.13-7.15 (m, 2H), 7.25-7.36 (m, 8H), 7.42-7.51 (m, 6H), 7.58-7.62 (m, 1H), 7.92-7.94 (m, 2H); ^13^C NMR (100 MHz, CDCl_3_) δ = 21.4, 25.9, 32.8, 36.0, 49.5, 62.7, 66.5, 69.2, 71.1, 74.0, 78.4, 127.5, 128.2, 128.2, 128.6, 128.6, 128.7, 129.1, 129.4, 132.9, 133.7, 134.5, 135.2, 138.2, 139.7, 144.7, 162.6, 166.8, 174.4, 198.4; HRMS (ESI) m/z: Calcd for C_41_H_37_NNaO_6_S [M+Na]^+^ 694.2234; Found 694.2222.

(1’*R*,2’*R*)-Benzhydryl spiro[(2-benzoyl-3-(4-chlorobenzyloxycarbonyl)cyclopent-3-ene)-1’,6-penicillanate] (**9k**) and (1’*R*,2’*R*)-Benzhydryl spiro[(2-benzoyl-5-(4-chlorobenzyloxycarbonyl)cyclopent-4-ene)-1’,6-penicillanate] (**10k**)

Obtained from 4-chlorobenzyl 2,3-butadienoate (**8k**) (0.065 mg, 0.31 mmol) and 6-alkylidenepenicillanate **7** (0.150 mg, 0.31 mmol) as described in the general procedure. The reaction mixture was stirred for 5 h. Purification of the crude product by flash chromatography [(ethyl acetate/hexane (1:5)] gave, in order of elution, **9k** as a colourless fluffy (0.045 g, 0.065 mmol, 21%) and **10k** as a colourless fluffy (0.083 g, 0.120 mmol, 39%).

Compound **9k**: mp 49.8-51.5 ^o^C; ${[\alpha]}_{D}^{25}$= + 320 (*c* 0.25 in CH_2_Cl_2_); IR (ATR): ν = 1773, 1744, 1715, 1667 cm ^–1^; ^1^H NMR (400 MHz, CDCl_3_) δ = 1.11 (s, 3H), 1.51 (s, 3H), 3.12 (dd, *J* = 18.8 and 3.2 Hz, 1H), 3.54 (dt, *J* = 18.8 and 2.4 Hz, 1H), 4.52 (s, 1H), 4.92 (d, *J* = 5.6 Hz, 2H), 5.16 (d, *J* = 1.2 Hz, 1H), 5.42 (s, 1H), 6.91 (s, 1H), 7.02-7.04 (m, 2H), 7.07 (br s, 1H), 7.21-7.24 (m, 2H), 7.29-7.35 (m, 8H), 7.38-7.41 (m, 2H), 7.47-7.60 (m, 2H), 7.80-7.82 (m, 1H), 8.02-8.05 (m, 2H); ^13^C NMR (100 MHz, CDCl_3_) δ = 26.1, 32.5, 40.8, 52.8, 64.3, 65.9, 69.1, 70.7, 71.1, 78.5, 127.1, 127.7, 128.3, 128.4, 128.5, 128.7, 128.8, 129.4, 129.9, 130.2, 132.6, 133.6, 133.7, 134.3, 135.7, 137.4, 139.2, 139.3, 146.3, 162.7, 167.0, 176.4, 201.1; HRMS (ESI) m/z: Calcd for C_40_ClH_34_NNaO_6_S [M+Na]^+^ 714.1688; Found 714.1680.

Compound **10k**: mp 86.0-88.3 ^o^C; ${[\alpha]}_{D}^{25}$= + 360 (*c* 0.375 in CH_2_Cl_2_); IR (ATR): ν = 1763, 1715, 1676, 1490 cm ^–1^; ^1^H NMR (400 MHz, CDCl_3_) δ = 1.11 (s, 3H), 1.52 (s, 3H), 2.52 (dd, *J* = 18.8 and 2.4 Hz, 1H), 3.19 (ddd, *J* = 18.8, 9.2 and 2.0 Hz, 1H), 4.55-4.57 (m, 2H), 5.16 (s, 2H) 6.25 (s, 1H), 6.91 (br s, 1H), 6.94 (s, 1H), 7.28-7.35 (m, 10H), 7.41-7.51 (m, 6H), 7.59-7.62 (m, 1H), 7.92-7.94 (m, 2H); ^13^C NMR (100 MHz, CDCl_3_) δ = 25.9, 32.7, 36.0, 49.6, 62.7, 65.8, 69.1, 71.0, 73.9, 78.4, 127.4, 127.6, 128.3, 128.6, 128.6, 128.7, 128.9, 129.1, 129.9, 133.7, 134.3, 134.3, 135.1, 139.5, 139.6, 145.2, 162.4, 166.8, 174.4, 198.3; HRMS (ESI) m/z: Calcd for C_40_ClH_33_NO_6_S [M-H] 690.1723; Found 690.1724.

(1’*R*,2’*R*)-Benzhydryl spiro[(2-benzoyl-3-(4-fluorobenzyloxycarbonyl)cyclopent-3-ene)-1’,6-penicillanate] (**9l**) and (1’*R*,2’*R*)-Benzhydryl spiro[(2-benzoyl-5-(4-fluorobenzyloxycarbonyl)cyclopent-4-ene)-1’,6-penicillanate] (**10l**)

Obtained from 4-fluorobenzyl 2,3-butadienoate (**8l**) (0.060 mg, 0.31 mmol) and 6-alkylidenepenicillanate **7** (0.150 mg, 0.31 mmol) as described in the general procedure. The reaction mixture was stirred for 7 h. Purification of the crude product by flash chromatography [(ethyl acetate/hexane (1:5)] gave, in order of elution, **9l** as a colourless fluffy (0.049 g, 0.073 mmol, 23%) and **10l** as a colourless fluffy (0.106 g, 0.157 mmol, 51%).

Compound **9l**: mp 67.2-69.3 ^o^C; ${[\alpha]}_{D}^{25}$= + 340 (*c* 0.25 in CH_2_Cl_2_); IR (ATR): ν = 1769, 1744, 1711, 1664 cm ^–1^; ^1^H NMR (400 MHz, CDCl_3_) δ = 1.11 (s, 3H), 1.51 (s, 3H), 3.11 (dd, *J* = 18.4 and 3.2 Hz, 1H), 3.54 (dt, *J* = 18.4 and 2.4 Hz, 1H), 4.52 (s, 1H), 4.92 (d, *J* = 3.2 Hz, 2H), 5.16 (d, *J* = 1.2 Hz, 1H), 5.42 (s, 1H), 6.91-6.96 (m, 3H), 7.06-7.10 (m, 3H), 7.29-7.35 (m, 10H), 7.38-7.42 (m, 2H), 7.54-7.57 (m, 1H), 8.03-8.06 (m, 2H); ^13^C NMR (100 MHz, CDCl_3_) δ = 26.1, 32.5, 40.8, 52.8, 64.3, 66.0, 69.1, 70.7, 71.2, 78.5, 115.6 (d, *J* = 22 Hz, 1C), 127.1, 127.7, 128.3, 128.4, 128.5, 128.7, 128.8, 129.4, 130.6 (d, *J* = 8 Hz, 2C), 131.2 (d, *J* = 3 Hz, 2C), 133.6, 135.8, 137.4, 139.2, 139.3, 146.1, 162.7 (d, *J* = 246 Hz, 1C), 162.8, 167.0, 176.4, 201.1; HRMS (ESI) m/z: Calcd for C_40_FH_34_NNaO_6_S [M+Na]^+^ 698.1983; Found 698.1971.

Compound **10l**: mp 157.3-159.0 ^o^C; ${[\alpha]}_{D}^{25}$= + 380 (*c* 0.3 in CH_2_Cl_2_); IR (ATR): ν = 1760, 1736, 1716, 1677 cm ^–1^; ^1^H NMR (400 MHz, CDCl_3_) δ = 1.11 (s, 3H), 1.52 (s, 3H), 2.51 (dd, *J* = 18.4 and 2.4 Hz, 1H), 3.19 (ddd, *J* = 18.4, 8.8 and 2.0 Hz, 1H), 4.55-4.57 (m, 2H), 5.16 (s, 2H) 6.25 (s, 1H), 6.90 (br s, 1H), 6.94 (s, 1H), 6.98-7.03 (s, 2H), 7.28-7.36 (m, 8H), 7.41-7.51 (m, 6H), 7.58-7.62 (m, 1H), 7.92-7.94 (m, 2H); ^13^C NMR (100 MHz, CDCl_3_) δ = 25.9, 32.7, 36.0, 49.6, 62.7, 65.9, 69.1, 71.0, 73.9, 78.4, 115.6 (d, *J* = 22 Hz, 1C), 127.4, 127.6, 128.3, 128.6, 128.7, 129.1, 129.4, 130.6 (d, *J* = 8 Hz, 2C), 131.7 (d, *J* = 3 Hz, 2C), 133.7, 134.4, 135.1, 139.6, 139.6, 145.1, 162.5, 162.8 (d, *J* = 245 Hz, 1C), 166.8, 174.4, 198.4; HRMS (ESI) m/z: Calcd for C_40_FH_34_NNaO_6_S [M+Na]^+^ 698.1983; Found 698.1975.

(1’*R*,2’*R*)-Benzhydryl spiro[(2-benzoyl-3-(prop-2-yn-1-yloxycarbonyl)cyclopent-3-ene)-1’,6-penicillanate] (**9m**) and (1’*R*,2’*R*)-Benzhydryl spiro[(2-benzoyl-5-(prop-2-yn-1-yloxycarbonyl)cyclopent-4-ene)-1’,6-penicillanate] (**10m**)

Obtained from propargyl 2,3-butadienoate (**8m**) (0.025 mg, 0.21 mmol) and 6-alkylidenepenicillanate **7** (0.100 mg, 0.21 mmol) as described in the general procedure. The reaction mixture was stirred for 6 h. Purification of the crude product by flash chromatography [(ethyl acetate/hexane (1:3)] gave, in order of elution, **9m** as a colourless solid (0.054 g, 0.085 mmol, 41%) and **10m** as a colourless solid (0.061 g, 0.102 mmol, 49%).

Compound **9m**: mp 62.2-64.8 ^o^C; ${[\alpha]}_{D}^{25}$= + 340 (*c* 0.25 in CH_2_Cl_2_); IR (ATR): ν = 1773, 1740, 1719, 1669 cm ^–1^; ^1^H NMR (400 MHz, CDCl_3_) δ = 1.12 (s, 3H), 1.52 (s, 3H), 2.38 (t, *J* = 2.4 Hz, 1H) 3.13 (dd, *J* = 18.8 and 3.2 Hz, 1H), 3.56 (dt, *J* = 18.4 and 2.4 Hz, 1H), 4.54 (d, *J* = 0.8 Hz, 2H), 4.55 (s, 1H), 5.18 (d, *J* = 1.2 Hz, 1H), 5.44 (s, 1H), 6.92 (s, 1H), 7.10 (br s, 1H), 7.28-7.35 (m, 10H), 7.45-7.49 (m, 2H), 7.57-7.60 (m, 1H), 8.10-8.12 (m, 2H); ^13^C NMR (100 MHz, CDCl_3_) δ = 26.1, 32.6, 40.8, 52.2, 52.9, 64.2, 69.1, 70.7, 71.1, 75.3, 78.5, 127.1, 127.7, 128.3, 128.5, 128.8, 129.5, 133.7, 135.2, 137.4, 139.2, 139.3, 146.9, 162.1, 167.0, 176.4, 201.0; HRMS (ESI) m/z: Calcd for C_36_H_31_NNaO_6_S [M+Na]^+^ 628.1764; Found 628.1756.

Compound **10m**: mp 78.0-79.9 ^o^C; ${[\alpha]}_{D}^{25}$= + 480 (*c* 0.25 in CH_2_Cl_2_); IR (ATR): ν = 1763, 1719, 1676, 1448 cm ^–1^; ^1^H NMR (400 MHz, CDCl_3_) δ = 1.13 (s, 3H), 1.52 (s, 3H), 2.44 (t, *J* = 2.4 Hz, 1H), 2.54 (dd, *J* = 18.8 and 2.4 Hz, 1H), 3.21 (ddd, *J* = 18.8, 9.2 and 2.0 Hz, 1H), 4.55-4.57 (m, 2H), 4.75 (t, *J* = 2.4 Hz, 2H), 6.28 (s, 1H), 6.95-6.97 (m, 2H), 7.27-7.37 (m, 6H), 7.41-7.52 (m, 6H), 7.59-7.63 (m, 1H), 7.93-7.95 (m, 2H); ^13^C NMR (100 MHz, CDCl_3_) δ =25.9, 33.0, 36.1, 49.4, 52.2, 62.8, 69.1, 71.0, 74.0, 75.3, 78.4, 127.4, 127.5, 128.2, 128.2, 128.5, 128.6, 128.7, 129.1, 133.7, 135.1, 139.6, 139.7, 145.7, 161.8, 166.8, 174.1, 198.3. HRMS (ESI) m/z: Calcd for C_36_H_31_NNaO_6_S [M+Na]^+^ 628.1764; Found 628.1755.

(1’*R*,2’*R*)-Benzhydryl spiro[(2-benzoyl-3-cinnamyloxycarbonylcyclopent-3-ene)-1’,6-penicillanate] (**9n**) and (1’*R*,2’*R*)-Benzhydryl spiro[(2-benzoyl-5-cinnamyloxycarbonylcyclopent-4-ene)-1’,6-penicillanate] (**10n**)

Obtained from (*E*)-cinnamyl 2,3-butadienoate (**8n**) (0.062 mg, 0.31 mmol) and 6-alkylidenepenicillanate **7** (0.150 mg, 0.31 mmol) as described in the general procedure. The reaction mixture was stirred for 72 h. Purification of the crude product by flash chromatography [(ethyl acetate/hexane (1:4)] gave, in order of elution, **9n** as a light yellow fluffy (0.052 g, 0.076 mmol, 25%) and **10n** as a light yellow fluffy (0.075 g, 0.110 mmol, 35%).

Compound **9n**: mp 67.8-69.4 ^o^C; ${[\alpha]}_{D}^{25}$= + 340 (*c* 0.25 in CH_2_Cl_2_); IR (ATR): ν = 1773, 1744, 1713, 1669 cm ^–1^; ^1^H NMR (400 MHz, CDCl_3_) δ = 1.12 (s, 3H), 1.52 (s, 3H), 3.12 (dd, *J* = 18.4 and 3.2 Hz, 1H), 3.55 (dt, *J* = 18.4 and 2.4 Hz, 1H), 4.52-4.62 (m, 3H), 5.18 (d, *J* = 1.2 Hz, 1H), 5.44 (s, 1H), 5.91-5.98 (m, 1H), 6.46 (d, *J* = 16.0 Hz, 1H), 6.92 (s, 1H), 7.08 (br s, 1H), 7.29-7.35 (m, 15H), 7.39-7.43 (m, 2H), 7.48-7.52 (m, 1H), 8.07-8.09 (m, 2H); ^13^C NMR (100 MHz, CDCl_3_) δ = 26.1, 32.6, 40.8, 53.0, 64.2, 65.5, 69.1, 70.7, 71.2, 78.4, 122.7, 126.8, 127.1, 127.7, 128.2, 128.3, 128.4, 128.5, 128.6, 128.7, 129.4, 133.6, 134.7, 136.0, 136.2, 137.5, 139.2, 139.3, 145.9, 162.8, 167.0, 176.5, 201.3; HRMS (ESI) m/z: Calcd for C_42_H_37_NNaO_6_S [M+Na]^+^ 706.2234; Found 706.2225.

Compound **10n**: mp 70.3-73.6 ^o^C; ${[\alpha]}_{D}^{25}$= + 400 (*c* 0.375 in CH_2_Cl_2_); IR (ATR): ν = 1763, 1714, 1672, 1448 cm ^–1^; ^1^H NMR (400 MHz, CDCl_3_) δ = 1.11 (s, 3H), 1.52 (s, 3H), 2.53 (dd, *J* = 18.8 and 2.8 Hz, 1H), 3.21 (ddd, *J* = 18.8, 9.2 and 2.0 Hz, 1H), 4.55-4.57 (m, 2H), 4.84 (dd, *J* = 6.8 and 1.2 Hz, 2H), 6.30 (s, 1H), 6.32-6.37 (m, 1H), 6.65 (d, *J* = 16.0 Hz, 1H), 6.92-6.94 (m, 2H), 7.27-7.41 (m, 13H), 7.45-7.51 (m, 4H), 7.58-7.62 (m, 1H), 7.93-7.95 (m, 2H); ^13^C NMR (100 MHz, CDCl_3_) δ = 26.1, 32.6, 40.8, 53.0, 64.2, 65.5, 69.1, 70.7, 71.2, 78.4, 122.7, 126.8, 127.1, 127.7, 128.2, 128.3, 128.4, 128.5, 128.6, 128.7, 129.4, 133.6, 134.7, 136.0, 136.2, 137.5, 139.2, 139.3, 145.9, 162.8, 167.0, 176.5, 201.3; HRMS (ESI) m/z: Calcd for C_42_H_37_NNaO_6_S [M+Na]^+^ 706.2234; Found 706.2222.

(1’*R*,2’*R*)-Benzhydryl spiro[(2-benzoyl-3-(1-phenylpropan-2-yloxycarbonyl)cyclopent-3-ene)-1’,6-penicillanate] (**9o**) and (1’*R*,2’*R*)-Benzhydryl spiro[(2-benzoyl-5-(1-phenylpropan-2-yloxycarbonyl)cyclopent-4-ene)-1’,6-penicillanate] (**10o**)

Obtained from 1-phenylpropan-2-yl 2,3-butadienoate (**8o**) (0.062 mg, 0.31 mmol) and 6-alkylidenepenicillanate **7** (0.150 mg, 0.31 mmol) as described in the general procedure. The reaction mixture was stirred for 7 h. Purification of the crude product by flash chromatography [(ethyl acetate/hexane (1:4)] gave, in order of elution, **9o** as a colourless fluffy (0.075 g, 0.109 mmol, 35%) and **10o** as a light yellow fluffy (0.116 g, 0.169 mmol, 55%).

Compound **9o**: IR (ATR): ν = 1773, 1744, 1713, 1669 cm ^–1^; ^1^H NMR (400 MHz, CDCl_3_) δ = 1.11 (s, 3H), 1.16 (m, 3H) 1.51 (s, 3H), 2.85-2.92 (m, 1H), 3.10 (dt, *J* = 18.4 and 1.2 Hz, 1H), 3.52 (dt, *J* = 18.8 and 2.4 Hz, 1H), 3.89-4.08 (m, 2H), 4.53 (d, *J* = 1.2 Hz, 1H), 5.11 (dd, *J* = 19.6 and 1.2 Hz, 1H), 5.43 (s, 1H), 6.92 (s, 1H), 6.94 (m, 1H), 7.11-7.14 (m, 2H), 7.19-7.22 (m, 1H), 7.25-7.35 (m, 12H), 7.42-7.48 (m, 2H), 7.57-7.60 (m, 1H), 8.0-8.02 (m, 1H), 8.06-8.09 (m, 1H); ^13^C NMR (100 MHz, CDCl_3_) δ = 18.0, 26.1, 32.6, 38.9, 40.8, 52.9, 64.2, 69.1, 69.7, 69.8, 70.7, 70.8, 71.2, 78.4, 126.9, 127.1, 127.3, 127.4, 127.7, 128.3, 128.4, 128.5, 128.6, 128.6, 128.8, 129.4, 129.5, 133.6, 136.1, 137.5, 139.3, 143.1, 145.4, 167.0, 201.2; HRMS (ESI) m/z: Calcd for C_42_H_39_NNaO_6_S [M+Na]^+^ 708.2390; Found 708.2379.

Compound **10o**: IR (ATR): ν = 1763, 1715, 1676, 1448 cm ^–1^; ^1^H NMR (400 MHz, CDCl_3_) δ = 1.11 (s, 3H), 1.26-1.31 (m, 3H), 1.51 (s, 3H), 2.49 (dd, *J* = 18.8 and 3.2 Hz, 1H), 3.13-3.18 (m, 2H), 4.21-4.29 (m, 2H), 4.53-4.55 (m, 2H), 6.24 (d, *J* = 6.4 Hz, 1H), 6.80 (t, *J* = 2.4 Hz, 1H), 6.95 (d, *J* = 2.4 Hz, 1H), 7.19-7.25 (m, 3H), 7.27-7.35 (m, 8H), 7.40-7.51 (m, 6H), 7.58-7.62 (m, 1H), 7.93-7.95 (m, 2H); ^13^C NMR (100 MHz, CDCl_3_) δ = 18.2, 25.9, 32.9, 33.1, 35.9, 39.0, 49.5, 62.7, 69.0, 69.8, 71.2, 74.1, 78.4, 126.8, 127.5, 128.2, 128.2, 128.6, 128.7, 128.7, 129.1, 133.7, 134.5, 134.6, 135.1, 135.2, 139.7, 143.2, 144.5, 162.7, 166.8, 174.4, 198.4; HRMS (ESI) m/z: Calcd for C_42_H_39_NNaO_6_S [M+Na]^+^ 708.2390; Found 708.2378.

(1’*R*,2’*R*)-Benzhydryl spiro[(2-(4-chlorobenzoyl)-3-benzyloxycarbonylcyclopent-3-ene)-1’,6-penicillanate] (**12b**) and (1’*R*,2’*R*)-Benzhydryl spiro[(2-(4-chlorobenzoyl)-5-benzyloxycarbonylcyclopent-4-ene)-1’,6-penicillanate] (**13b**)

Obtained from allene **8p** (65 mg, 0.375 mmol) and 6-alkylidenepenicillanate **11b** (194 mg, 0.375 mmol) as described in the general procedure (reaction time: 4.5 h). Purification of the crude product by flash chromatography (hexane/ethyl acetate, 4:1), gave, in order of elution, **12b** as a colourless solid (130 mg, 0.188 mmol, 50%) and **13b** as a colourless solid (116 mg, 0.168 mmol, 45%).

Compound **12b**: mp 145.2-146.5 ºC; ${[\alpha]}_{D}^{25}$ = + 290 (c 0.5 in CH_2_Cl_2_); IR (ATR): $\tilde{\nu}$ = 1000, 1065, 1177, 1198, 1236, 1328, 1456, 1588, 1668, 1718, 1735 and 1773 cm^–1^; ^1^H NMR (400 MHz, CDCl_3_): δ = 1.11 (s, 3H), 1.50 (s, 3H), 3.11 (dd, *J* = 18.6 and 3.1 Hz, 1H), 3.52 (dt, *J* = 18.6 and 2.3 Hz, 1H), 4.52 (s, 1H), 4.95 (d, *J* = 12.2 Hz, 1H), 5.00 (d, *J* = 12.2 Hz, 1H), 5.09 (d, *J* = 1.0 Hz, 1H), 5.40 (s, 1H), 6.91 (s, 1H), 7.08 (s, 1H), 7.14 (dd, *J* = 7.5 and 1.9 Hz, 2H), 7.29-7.35 (m, 14H), 7.97 (d, *J* = 8.7 Hz, 2H); ^13^C NMR (100 MHz, CDCl_3_): δ = 26.1, 32.5, 40.8, 52.8, 64.4, 66.9, 69.1, 70.8, 71.1, 78.5, 127.1, 127.7, 128.4, 128.5, 128.6, 128.7, 128.8, 130.1, 135.2, 135.7, 139.2, 139.3, 140.1, 146.2, 162.9, 165.5, 166.9, 170.3, 176.3, 200.1; HRMS (ESI-TOF) m/z: [M+NH_4_]^+^ Calcd C_40_H_38_ClN_2_O_6_S 709.2134, found 709.2125.

Compound **13b**: mp 116.3-118.3 ºC; ${[\alpha]}_{D}^{25}$ = + 370 (c 0.5 in CH_2_Cl_2_); IR (ATR): $\tilde{\nu}$ = 977, 1011, 1088, 1176, 1200, 1306, 1453, 1589, 1676, 1718, 1744 and 1774 cm^–1^; ^1^H NMR (400 MHz, CDCl_3_): δ = 1.11 (s, 3H), 1.51 (s, 3H), 2.47 (dd, *J* = 18.7 and 2.5 Hz, 1H), 3.18 (ddd, *J* = 18.8, 9.1 and 2.0 Hz, 1H), 4.49 (d, *J* = 8.4 Hz, 1H), 4.54 (s, 1H), 5.21 (s, 2H), 6.26 (s, 1H), 6.90 (t, *J* = 2.5 Hz, 1H), 6.93 (s, 1H), 7.27-7.49 (m, 17H), 7.87 (d, *J* = 8.6 Hz, 2H); ^13^C NMR (100 MHz, CDCl_3_): δ = 25.9, 32.9, 35.9, 49.4, 62.8, 66.6, 69.1, 71.0, 74.1, 78.4, 127.5, 127.5, 128.2, 128.3, 128.4, 128.5, 128.7, 128.7, 129.4, 130.0, 133.5, 134.4, 135.8, 139.6, 140.3, 144.7, 162.5, 166.7, 174.2, 197.3; HRMS (ESI-TOF) m/z: [M+NH_4_]^+^ Calcd C_40_H_38_ClN_2_O_6_S 709.2134, found 709.2131.

(1’*R*,2’*R*)-Benzhydryl spiro[(2-(4-bromobenzoyl)-3-benzyloxycarbonylcyclopent-3-ene)-1’,6-penicillanate] (**12c**) and (1’*R*,2’*R*)-Benzhydryl spiro[(2-(4-bromobenzoyl)-5-benzyloxycarbonylcyclopent-4-ene)-1’,6-penicillanate] (**13c**)

Obtained from allene **8p** (65 mg, 0.375 mmol) and 6-alkylidenepenicillanate **11c** (211 mg, 0.375 mmol) as described in the general procedure (reaction time: 6.5 h). Purification of the crude product by flash chromatography (hexane/ethyl acetate, 4:1), gave, in order of elution, **12c** as a colourless solid (129 mg, 0.175 mmol, 47%) and **13c** as a colourless solid (109 mg, 0.148 mmol, 39%).

Compound **12c**: mp 156.5-158.5 ºC; ${[\alpha]}_{D}^{25}$ = + 360 (c 0.5 in CH_2_Cl_2_); IR (ATR): $\tilde{\nu}$ = 998, 1068, 1177, 1201, 1237, 1330, 1456, 1583, 1670, 1718 and 1773 cm^–1^; ^1^H NMR (400 MHz, CDCl_3_): δ = 1.11 (s, 3H), 1.50 (s, 3H), 3.11 (dd, *J* = 18.6 and 3.1 Hz, 1H), 3.52 (dt, *J* = 18.6 and 2.2 Hz, 1H), 4.52 (s, 1H), 4.95 (d, *J* = 12.2 Hz, 1H), 5.00 (d, *J* = 12.2 Hz, 1H), 5.08 (d, *J* = 1.1 Hz, 1H), 5.40 (s, 1H), 6.91 (s, 1H), 7.08 (s, 1H), 7.14 (dd, *J* = 7.5 and 1.8 Hz, 2H), 7.27-7.37 (m, 14H), 7.47 (d, *J* = 8.6 Hz, 2H), 7.89 (d, *J* = 8.6 Hz, 2H); ^13^C NMR (100 MHz, CDCl_3_): δ = 26.1, 32.5, 40.8, 52.8, 64.4, 67.0, 69.1, 70.8, 71.1, 77.2, 78.5, 127.1, 127.7, 128.4, 128.6, 128.7, 128.8, 129.0, 130.8, 131.8, 135.2, 135.7, 136.1, 139.2, 139.3, 146.3, 162.9, 166.9, 176.3, 200.3; HRMS (ESI-TOF) m/z: [M-H]^+^ Calcd C_40_H_33_BrNO_6_S 734.1217, found 734.1229.

Compound **13c**: mp 115.9-117.9 ºC; ${[\alpha]}_{D}^{25}$ = + 380 (c 0.5 in CH_2_Cl_2_); IR (ATR): $\tilde{\nu}$ = 976, 1007, 1069, 1176, 1199, 1305, 1374, 1453, 1584, 1676, 1718, 1744 and 1774 cm^–1^; ^1^H NMR (400 MHz, CDCl_3_): δ = 1.11 (s, 3H), 1.51 (s, 3H), 2.47 (dd, *J* = 18.5 and 2.7 Hz, 1H), 3.18 (dd, *J* = 18.6 and 9.0 Hz, 1H), 4.48 (d, *J* = 8.9 Hz, 1H), 4.54 (s, 1H), 5.21 (s, 2H), 6.26 (s, 1H), 6.90 (s, 1H), 6.93 (s, 1H), 7.27-7.37 (m, 11H), 7.44 (dd, *J* = 7.3 and 17.0 Hz, 4H), 7.63 (d, *J* = 8.4 Hz, 2H), 7.79 (d, *J* = 8.4 Hz, 2H); ^13^C NMR (100 MHz, CDCl_3_): δ = 25.9, 32.9, 35.8, 49.4, 62.8, 66.6, 69.1, 71.0, 74.1, 78.4, 127.5, 127.5, 128.2, 128.3, 128.4, 128.5, 128.7, 128.7, 129.0, 130.0, 132.4, 133.9, 134.4, 135.8, 139.6, 144.7, 162.5, 166.7, 174.1, 197.4; HRMS (ESI-TOF) m/z: [M+H]^+^ Calcd C_40_H_35_BrNO_6_S 736.1363, found 736.1362.

(1’*R*,2’*R*)-Benzhydryl spiro[(2-(4-nitrobenzoyl)-3-benzyloxycarbonylcyclopent-3-ene)-1’,6-penicillanate] (**12d**) and (1’*R*,2’*R*)-Benzhydryl spiro[(2-(4-nitrobenzoyl)-5-benzyloxycarbonylcyclopent-4-ene)-1’,6-penicillanate] (**13d**)

Obtained from allene **8p** (65 mg, 0.375 mmol) and 6-alkylidenepenicillanate **11d** (198 mg, 0.375 mmol) as described in the general procedure (reaction time: 5.5 h). Purification of the crude product by flash chromatography (hexane/ethyl acetate, 3:1), gave, in order of elution, **12d** as a colourless oil (138 mg, 0.196 mmol, 52%) and **13d** as a colourless solid (77 mg, 0.109 mmol, 29%).

Compound **12d**: mp 122.0-123.7 ºC; ${[\alpha]}_{D}^{25}$ = + 310 (c 0.5 in CH_2_Cl_2_); IR (ATR): $\tilde{\nu}$ = 845, 950, 1004, 1066, 1179, 1316, 1521, 1675, 1734 and 1759 cm^–1^; ^1^H NMR (400 MHz, CDCl_3_): δ = 1.11 (s, 3H), 1.49 (s, 3H), 3.14 (dd, *J* = 18.7 and 3.1 Hz, 1H), 3.52 (dt, *J* = 18.7 and 2.3 Hz, 1H), 4.54 (s, 1H), 4.92 (d, *J* = 12.1 Hz, 1H), 4.99 (d, *J* = 12.1 Hz, 1H), 5.13 (d, *J* = 1.1 Hz, 1H), 5.40 (s, 1H), 6.91 (s, 1H), 7.09-7.13 (m, 3H), 7.26-7.37 (m, 13H), 8.12 (s, 4H); ^13^C NMR (100 MHz, CDCl_3_): δ = 26.1, 32.8, 40.7, 53.3, 64.8, 67.1, 69.1, 71.1, 71.1, 78.6, 123.6, 127.1, 127.7, 128.4, 128.6, 128.7, 128.7, 128.8, 130.1, 134.9, 135.4, 139.1, 139.2, 141.7, 146.7, 150.4, 162.8, 166.8, 175.8, 200.2; HRMS (ESI-TOF) m/z: [M+H]^+^ Calcd C_40_H_38_N_3_O_8_S 720.2374, found 720.2368.

Compound **13d**: mp 122.7-124.7 ºC; ${[\alpha]}_{D}^{25}$ = + 330 (c 0.5 in CH_2_Cl_2_); IR (ATR): $\tilde{\nu}$ = 1013, 1154, 1179, 1201, 1313, 1347, 1528, 1686, 1723, 1743 and 1776 cm^–1^; ^1^H NMR (400 MHz, CDCl_3_): δ = 1.11 (s, 3H), 1.51 (s, 3H), 2.48 (dd, *J* = 18.5 and 2.5 Hz, 1H), 3.18 (ddd, *J* = 18.6, 9.1 and 2.0 Hz, 1H), 4.53 (d, *J* = 8.5 Hz, 1H), 4.56 (s, 1H), 5.22 (s, 2H), 6.26 (s, 1H), 6.90-6.92 (s, 1H), 6.94 (s, 1H), 7.27-7.39 (m, 11H), 7.41 (d, *J* = 7.1, 2H), 7.46 (d, *J* = 7.1, 2H), 8.09 (d, *J* = 8.9 Hz, 2H), 8.34 (d, *J* = 8.9 Hz, 2H); ^13^C NMR (100 MHz, CDCl_3_): δ = 25.8, 33.1, 35.5, 49.8, 63.2, 66.7, 69.1, 71.0, 74.5, 78.5, 124.3, 127.4, 127.5, 128.3, 128.3, 128.5, 128.5, 128.7, 128.7, 128.7, 129.6, 134.4, 135.7, 139.6, 139.9, 144.3, 150.7, 162.4, 166.6, 173.7, 197.0; HRMS (ESI-TOF) m/z: [M+H]^+^ Calcd C_40_H_38_N_3_O_8_S 720.2374, found 720.2366.

(1’*R*,2’*R*)-Benzhydryl spiro[(2-(4-trifluoromethylbenzoyl)-3-benzyloxycarbonylcyclopent-3-ene)-1’,6-penicillanate] (**12e**) and (1’*R*,2’*R*)-Benzhydryl spiro[(2-(4-trifluoromethylbenzoyl)-5-benzyloxycarbonylcyclopent-4-ene)-1’,6-penicillanate] (**13e**)

Obtained from allene **8p** (65 mg, 0.375 mmol) and 6-alkylidenepenicillanate **11e** (207 mg, 0.375 mmol) as described in the general procedure (reaction time: 5.5 h). Purification of the crude product by flash chromatography (hexane/ethyl acetate, 4:1), gave, in order of elution, **12e** as a colourless solid (143 mg, 0.196 mmol, 52%) and **13e** as a colourless solid (96 mg, 0.133 mmol, 35%).

Compound **12e**: mp 134.1-135.5 ºC; ${[\alpha]}_{D}^{25}$ = + 320 (c 0.5 in CH_2_Cl_2_); IR (ATR): $\tilde{\nu}$ = 1004, 1065, 1135, 1320, 1675, 1735 and 1760 cm^–1^; ^1^H NMR (400 MHz, CDCl_3_): δ = 1.12 (s, 3H), 1.50 (s, 3H), 3.13 (dd, *J* = 18.7 and 3.1 Hz, 1H), 3.53 (dt, *J* = 18.6 and 2.3 Hz, 1H), 4.54 (s, 1H), 4.95 (d, *J* = 12.2 Hz, 1H), 4.99 (d, *J* = 12.2 Hz, 1H), 5.14 (d, *J* = 1.1 Hz, 1H), 5.41 (s, 1H), 6.92 (s, 1H), 7.10-7.14 (m, 3H), 7.27-7.35 (m, 12H), 7.60 (d, *J* = 8.3 Hz, 2H), 8.13 (d, *J* = 8.3 Hz, 2H); ^13^C NMR (100 MHz, CDCl_3_): δ = 26.1, 32.7, 40.7, 53.0, 64.6, 67.0, 69.1, 70.1, 71.1, 78.5, 123.8 (d, *J* = 272.8 Hz, 1C), 125.5, 125.5, 127.1, 127.7, 128.4, 128.6, 128.6, 128.6, 128.7, 128.8, 129.5, 134.4, 134.7, 135.1, 135.6, 139.2, 139.3, 140.0, 146.4, 162.8, 166.9, 176.1, 200.5; ^19^F NMR (376 MHz, CDCl_3_): δ = -63.03 (s, 3F); HRMS (ESI-TOF) m/z: [M+NH_4_]^+^ Calcd C_41_H_38_F_3_N_2_O_6_S 743.2397, found 743.2387.

Compound **13e**: mp 141.1-143.1 ºC; ${[\alpha]}_{D}^{25}$ = + 330 (c 0.5 in CH_2_Cl_2_); IR (ATR): $\tilde{\nu}$ = 1013, 1067, 1131, 1171, 1319, 1453, 1684, 1719, 1744 and 1776 cm^–1^; ^1^H NMR (400 MHz, CDCl_3_): δ = 1.11 (s, 3H), 1.52 (s, 3H), 2.48 (dd, *J* = 19.0 and 2.7 Hz, 1H), 3.20 (ddd, *J* = 18.6, 9.1 and 2.0 Hz, 1H), 4.54 (d, *J* = 8.6 Hz, 1H), 4.56 (s, 1H), 5.22 (s, 2H), 6.27 (s, 1H), 6.89-6.92 (s, 1H), 6.94 (s, 1H), 7.27-7.38 (m, 11H), 7.42 (d, *J* = 7.2, 2H), 7.46 (d, *J* = 7.2, 2H), 7.76 (d, *J* = 8.3 Hz, 2H), 8.04 (d, *J* = 8.3 Hz, 2H); ^13^C NMR (100 MHz, CDCl_3_): δ = 25.9, 33.0, 35.6, 49.6, 63.0, 66.7, 69.1, 71.0, 74.2, 78.4, 123.6 (d, *J* = 272.9 Hz, 1C), 126.2, 126.2, 127.5, 127.5, 128.2, 128.3, 128.4, 128.5, 128.7, 128.7, 128.7, 128.9, 134.4, 134.8, 135.1, 135.8, 138.0, 139.6, 144.5, 162.4, 166.7, 173.9, 197.4; ^19^F NMR (376 MHz, CDCl_3_): δ = -66.18 (s, 3F); HRMS (ESI-TOF) m/z: [M+NH_4_]^+^ Calcd C_41_H_38_F_3_N_2_O_6_S 743.2397, found 743.2386.

(1’*R*,2’*R*)-Benzhydryl spiro[(2-(3,5-difluorobenzoyl)-3-benzyloxycarbonylcyclopent-3-ene)-1’,6-penicillanate] (**12f**) and (1’*R*,2’*R*)-Benzhydryl spiro[(2-(3,5-difluorobenzoyl)-5-benzyloxycarbonylcyclopent-4-ene)-1’,6-penicillanate] (**13f**)

Obtained from allene **8p** (65 mg, 0.375 mmol) and 6-alkylidenepenicillanate **11f** (195 mg, 0.375 mmol) as described in the general procedure (reaction time: 6.5 h). Purification of the crude product by flash chromatography (hexane/ethyl acetate, 4:1), gave, in order of elution, **12f** as a colourless solid (138 mg, 0.198 mmol, 53%) and **13f** as a colourless solid (116 mg, 0.167 mmol, 44%).

Compound **12f**: mp 69.5-71.5 ºC; ${[\alpha]}_{D}^{25}$ = + 320 (c 0.5 in CH_2_Cl_2_); IR (ATR): $\tilde{\nu}$ = 984, 1065, 1120, 1333, 1438, 1593, 1676, 1716, 1741 and 1773 cm^–1^; ^1^H NMR (400 MHz, CDCl_3_): δ = 1.13 (s, 3H), 1.52 (s, 3H), 3.12 (dd, *J* = 18.7 and 3.1 Hz, 1H), 3.50 (dt, *J* = 18.7 and 2.3 Hz, 1H), 4.54 (s, 1H), 4.97 (d, *J* = 12.2 Hz, 1H), 4.99 (s, 1H), 5.04 (d, *J* = 12.2 Hz, 1H), 5.39 (s, 1H), 6.92 (s, 1H), 6.93-6.98 (m, 1H), 7.08 (s, 1H), 7.15-7.18 (m, 2H), 7.29-7.35 (m, 13H), 7.55 (dd, *J* = 7.9 and 2.1 Hz, 2H); ^13^C NMR (100 MHz, CDCl_3_): δ = 26.1, 32.8, 40.7, 53.2, 64.6, 66.9, 69.1, 71.0, 71.1, 78.5, 108.8 (t, *J* = 25.4 Hz, 1C), 112.2 (d, *J* = 16.6 Hz, 1C), 112.2 (d, *J* = 26.2 Hz, 1C), 127.1, 127.7, 128.4, 128.6, 128.7, 128.8, 135.1, 135.5, 139.1, 139.3, 140.1, 140.5 (t, *J* = 7.9 Hz, 1C), 146.3, 162.8, 162.8 (d, *J* = 250.2 Hz, 1C), 162.8 (d, *J* = 250.0 Hz, 1C), 166.8, 175.9, 199.2; ^19^F NMR (376 MHz, CDCl_3_): δ = -108.43 (s, 2F); HRMS (ESI-TOF) m/z: [M+NH_4_]^+^ Calcd C_40_H_37_F_2_N_2_O_6_S 711.2335, found 711.2333.

Compound **13f**: mp 134.8-136.8 ºC; ${[\alpha]}_{D}^{25}$ = + 270 (c 0.5 in CH_2_Cl_2_); IR (ATR): $\tilde{\nu}$ = 987, 1119, 1257, 1304, 1333, 1438, 1593, 1686, 1718 and 1769 cm^–1^; ^1^H NMR (400 MHz, CDCl_3_): δ = 1.11 (s, 3H), 1.53 (s, 3H), 2.47 (dd, *J* = 19.0 and 2.7 Hz, 1H), 3.19 (ddd, *J* = 18.6, 9.1 and 2.1 Hz, 1H), 4.40 (d, *J* = 8.3 Hz, 1H), 4.56 (s, 1H), 5.21 (s, 2H), 6.25 (s, 1H), 6.90 (t, *J* = 2.5 Hz, 1H), 6.94 (s, 1H), 7.05 (tt, *J* = 8.4 and 2.3 Hz, 1H), 7.27-7.38 (m, 11H), 7.39-7.49 (m, 6H); ^13^C NMR (100 MHz, CDCl_3_): δ = 25.9, 33.0, 35.6, 49.7, 63.0, 66.7, 69.1, 71.0, 74.2, 78.4, 109.1 (t, *J* = 25 Hz, 1C), 111.4 (d, *J* = 8 Hz, 1C), 111.6 (d, *J* = 7 Hz, 1C), 127.5, 127.5, 128.2, 128.3, 128.4, 128.5, 128.7, 128.7, 128.7, 134.4, 135.8, 138.2 (t, *J* = 8 Hz, 1C), 139.6, 144.4, 162.4, 163.3 (d, *J* = 252 Hz, 1C), 163.4 (d, *J* = 252 Hz, 1C), 166.6, 173.8, 196.0; ^19^F NMR (376 MHz, CDCl_3_): δ = -107.21 (s, 2F); HRMS (ESI-TOF) m/z: [M+H]^+^ Calcd C_40_H_34_F_2_NO_6_S 694.2069, found 694.2067.

(1’*R*,2’*R*)-Benzhydryl spiro[(2-(3,5-bis(trifluoromethyl)benzoyl)-3-benzyloxycarbonylcyclopent-3-ene)-1’,6-penicillanate] (**12g**) and (1’*R*,2’*R*)-Benzhydryl spiro[(2-(3,5-bis(trifluoromethyl)benzoyl)-5-benzyloxycarbonylcyclopent-4-ene)-1’,6-penicillanate] (**13g**)

Obtained from allene **8p** (65 mg, 0.375 mmol) and 6-alkylidenepenicillanate **11g** (232 mg, 0.375 mmol) as described in the general procedure (reaction time: 5 h). Purification of the crude product by flash chromatography (hexane/ethyl acetate, 4:1), gave a mixture of **12g**/**13g** as a yellow oil (197 mg, 0.248 mmol, 66%), in a 59/41 ratio.

Compound **12g**: ^1^H NMR (400 MHz, CDCl_3_): δ = 1.12 (s, 3H), 1.51 (s, 3H), 3.15 (dd, *J* = 18.7 and 3.1 Hz, 1H), 3.51 (dt, *J* = 18.7 and 2.2 Hz, 1H), 4.58 (s, 1H), 4.88 (d, *J* = 12.1 Hz, 1H), 5.23 (s, 1H), 5.05 (d, *J* = 12.2 Hz, 1H), 5.41 (s, 1H), 6.91 (s, 1H), 7.06-7.10 (m, 2H), 7.23-7.36 (m, 14H), 7.98 (s, 1H), 8.46 (s, 2H); HRMS (ESI-TOF) m/z: [M+Cl]^+^ Calcd C_42_H_33_NO_6_SF_6_Cl 828.1627, found 828.1638.

Compound **13g**: ^1^H NMR (400 MHz, CDCl_3_): δ = 1.12 (s, 3H), 1.54 (s, 3H), 2.46 (dd, *J* = 19.0 and 3.3 Hz, 1H), 3.22 (ddd, *J* = 18.4, 9.1 and 1.8 Hz, 1H), 4.52 (d, *J* = 8.3 Hz, 1H),

4.58 (s, 1H), 5.12 (s, 2H), 6.26 (s, 1H), 6.94 (s, 1H), 7.06-7.10 (m, 2H), 7.23-7.26 (m, 14H), 8.10 (s, 1H), 8.34 (s, 2H); HRMS (ESI-TOF) m/z: [M+Cl]^+^ Calcd C_42_H_33_NO_6_SF_6_Cl 828.1627, found 828.1638.

(1’*R*,2’*R*)-Benzhydryl spiro[(2-naphthoyl-3-benzyloxycarbonylcyclopent-3-ene)-1’,6-penicillanate] (**12h**) and (1’*R*,2’*R*)-Benzhydryl spiro[(2-naphthoyl-5-benzyloxycarbonylcyclopent-4-ene)-1’,6-penicillanate] (**13h**)

Obtained from allene **8p** (65 mg, 0.375 mmol) and 6-alkylidenepenicillanate **11h** (200 mg, 0.375 mmol) as described in the general procedure (reaction time: 4 h). Purification of the crude product by flash chromatography (hexane/ethyl acetate, 5:1), gave, in order of elution, **12h** as a yellow solid (111 mg, 0.158 mmol, 42%) and **13h** as a fluffy yellow solid (114 mg, 0.161 mmol, 43%).

Compound **12h**: mp 81.5-83.5 ºC; ${[\alpha]}_{D}^{25}$ = + 340 (c 0.25 in CH_2_Cl_2_); IR (ATR): $\tilde{\nu}$ = 1175, 1247, 1330, 1457, 1654, 1707, 1718 and 1773 cm^–1^; ^1^H NMR (400 MHz, CDCl_3_): δ = 1.07 (s, 3H), 1.49 (s, 3H), 3.15 (dd, *J* = 18.6 and 3.1 Hz, 1H), 3.58 (dt, *J* = 18.6 and 2.2 Hz, 1H), 4.53 (s, 1H), 4.91 (s, 2H), 5.33 (s, 1H), 5.44 (s, 1H), 6.91 (s, 1H), 7.00-7.02 (m, 2H), 7.10-7.16 (m, 4H), 7.28-7.34 (m, 10H), 7.52-7.56 (m, 1H), 7.59-7.63 (m, 1H), 7.80-7.92 (m, 3H), 8.05 (dd, *J* = 1.7 and 8.7 Hz, 1H), 8.66 (s, 1H); ^13^C NMR (100 MHz, CDCl_3_): δ = 26.1, 32.4, 40.9, 52.9, 64.3, 66.7, 69.1, 70.7, 71.2, 78.4, 124.7, 126.8, 127.1, 127.7, 127.8, 128.2, 128.3, 128.3, 128.4, 128.5, 128.5, 128.8, 128.8, 130.2, 131.8, 132.6, 134.8, 135.2, 136.0, 136.0, 139.2, 139.3, 146.0, 162.9, 167.0, 176.6, 201.0; HRMS (ESI-TOF) m/z: [M+NH_4_]^+^ Calcd C_44_H_41_N_2_O_6_S 725.2680, found 725.2676.

Compound **13h**: mp 90.8-92.8 ºC; ${[\alpha]}_{D}^{25}$ = + 340 (c 0.25 in CH_2_Cl_2_); IR (ATR): $\tilde{\nu}$ = 984, 1118, 1251, 1323, 1457, 1670, 1718 and 1763 cm^–1^; ^1^H NMR (400 MHz, CDCl_3_): δ = 1.09 (s, 3H), 1.52 (s, 3H), 2.59 (dd, *J* = 18.8 and 2.5 Hz, 1H), 3.27 (ddd, *J* = 18.6, 9.1 and 2.0 Hz, 1H), 4.56 (s, 1H), 4.72 (d, *J* = 8.5 Hz, 1H), 5.23 (s, 2H), 6.32 (s, 1H), 6.94-6.95 (m, 2H), 7.27-7.40 (m, 11H), 7.41-7.44 (m, 2H), 7.46-7.48 (m, 2H), 7.56-7.65 (m, 2H), 7.88-7.94 (m, 2H), 7.97-8.01 (m, 2H), 8.46 (s, 1H); ^13^C NMR (100 MHz, CDCl_3_): δ = 25.9, 32.7, 36.3, 49.6, 62.7, 66.6, 69.2, 71.1, 74.1, 78.4, 124.2, 127.2, 127.5, 127.5, 128.0, 128.2, 128.2, 128.4, 128.5, 128.7, 128.7, 129.0, 129.8, 130.2, 132.5, 132.7, 134.4, 135.9, 139.7, 145.0, 162.6, 166.8, 174.5, 198.4; HRMS (ESI-TOF) m/z: [M+H]^+^ Calcd C_44_H_38_NO_6_S 708.2414, found 708.2410.

(1’*R*,2’*R*)-Benzhydryl spiro[(2-furoyl-3-benzyloxycarbonylcyclopent-3-ene)-1’,6-penicillanate] (**12i**) and (1’*R*,2’*R*)-Benzhydryl spiro[(2-furoyl-5-benzyloxycarbonylcyclopent-4-ene)-1’,6-penicillanate] (**13i**)

Obtained from allene **8p** (65 mg, 0.375 mmol) and 6-alkylidenepenicillanate **11i** (177 mg, 0.375 mmol) as described in the general procedure (reaction time: 7 h). Purification of the crude product by flash chromatography (hexane/ethyl acetate, 2:1), gave, in order of elution, **12i** as a colourless solid (81 mg, 0.12 mmol, 33%) and **13i** as a colourless solid (134 mg, 0.21 mmol, 55%).

Compound **12i**: mp 76.5-78.5 ºC; ${[\alpha]}_{D}^{25}$ = + 360 (c 0.25 in CH_2_Cl_2_); IR (ATR): $\tilde{\nu}$ = 985, 1177, 1202, 1234, 1292, 1331, 1463, 1560, 1662, 1716 and 1773 cm^–1^; ^1^H NMR (400 MHz, CDCl_3_): δ = 1.14 (s, 3H), 1.51 (s, 3H), 3.09 (dd, *J* = 18.6 and 3.1 Hz, 1H), 3.48 (dt, *J* = 18.6 and 2.3 Hz, 1H), 4.53 (s, 1H), 4.92 (d, *J* = 1.0 Hz, 2H), 5.01 (d, *J* = 12.3 Hz, 1H), 5.06 (d, *J* = 12.3 Hz, 1H), 5.41 (s, 1H), 6.47 (dd, *J* = 3.6 and 1.6 Hz, 1H), 6.91 (s, 1H), 7.06 (s, 1H), 7.22 (dd, *J* = 6.7 and 2.8 Hz, 2H), 7.28-7.35 (m, 14H), 7.51 (d, *J* = 1.0, 2H); ^13^C NMR (100 MHz, CDCl_3_): δ = 26.0, 32.8, 41.0, 53.7, 64.1, 66.8, 69.1, 70.2, 71.1, 78.5, 112.8, 119.3, 127.1, 127.7, 128.3, 128.4, 128.5, 128.6, 128.7, 128.7, 128.8, 135.4, 135.6, 139.2, 139.3, 145.9, 147.0, 153.2, 162.9, 166.9, 176.1, 188.6; HRMS (ESI-TOF) m/z: [M+NH_4_]^+^ Calcd C_38_H_37_N_2_O_7_S 665.2316, found 665.2310.

Compound **13i**: mp 112.8-114.8 ºC; ${[\alpha]}_{D}^{25}$ = + 370 (c 0.5 in CH_2_Cl_2_); IR (ATR): $\tilde{\nu}$ = 985, 1055, 1133, 1177, 1254, 1330, 1457, 1654, 1720, 1749 and 1770 cm^–1^; ^1^H NMR (400 MHz, CDCl_3_): δ = 1.12 (s, 3H), 1.52 (s, 3H), 2.54 (dd, *J* = 18.8 and 2.2 Hz, 1H), 3.16 (ddd, *J* = 18.8, 9.0 and 2.1 Hz, 1H), 4.38 (d, *J* = 8.1 Hz, 1H), 4.55 (s, 1H), 5.20 (s, 2H), 6.25 (s, 1H), 6.57 (dd, *J* = 3.6 and 1.7 Hz, 1H), 6.93 (d, *J* = 4.7 Hz, 2H), 7.23 (d, *J* = 3.6 Hz, 1H), 7.27-7.37 (m, 11H), 7.42 (d, *J* = 7.2 Hz, 2H), 7.46 (d, *J* = 7.2 Hz, 2H), 7.60 (d, *J* = 1.0 Hz, 1H); ^13^C NMR (100 MHz, CDCl_3_): δ = 25.8, 33.1, 36.1, 49.9, 62.8, 66.5, 69.1, 71.0, 73.6, 78.4, 112.8, 117.8, 127.5, 127.5, 128.2, 128.2, 128.3, 128.4, 128.7, 128.7, 134.2, 135.9, 139.7, 145.1, 146.7, 151.7, 162.5, 166.8, 174.1, 187.8; HRMS (ESI-TOF) m/z: [M+NH_4_]^+^ Calcd C_38_H_37_N_2_O_7_S 665.2316, found 665.2309.

(1’*R*,2’*R*)-Benzhydryl spiro[(2-benzhydryloxycarbonyl-3-benzyloxycarbonylcyclopent-3-ene)-1’,6-penicillanate] (**12j**) and (1’*R*,2’*R*)-Benzhydryl spiro[(2-benzhydryloxycarbonyl-5-benzyloxycarbonylcyclopent-4-ene)-1’,6-penicillanate] (**13j**)

Obtained from allene **8p** (65 mg, 0.375 mmol) and 6-alkylidenepenicillanate **11j** (221 mg, 0.375 mmol) as described in the general procedure (reaction time: 5 h). Purification of the crude product by flash chromatography (hexane/ethyl acetate, 3:1), gave a mixture of **12j**/**13j** as a yellow oil (270 mg, 0.353 mmol, 94%) in a 50/50 ratio.

Compound **12j**: ^1^H NMR (400 MHz, CDCl_3_): δ = 1.11 (s, 3H), 1.47 (s, 3H), 3.02 (dd, *J* = 18.5 and 3.1 Hz, 1H), 3.30 (dt, *J* = 18.4 and 2.1 Hz, 1H), 4.19 (d, *J* = 0.9 Hz, 1H), 4.49 (s, 1H), 5.06 (d, *J* = 12.4 Hz, 1H), 5.15 (d, *J* = 12.4 Hz, 1H), 5.33 (s, 1H), 6.82 (s, 1H), 6.90 (s, 1H), 6.97-6.99 (m, 1H), 7.24-7.40 (m, 24H), 7.43-4.47 (m, 1H); HRMS (ESI-TOF) m/z: [M+NH_4_]^+^ Calcd C_47_H_45_N_2_O_7_S 781.2942, found 781.2932.

Compound **13j**: ^1^H NMR (400 MHz, CDCl_3_): δ = 1.07 (s, 3H), 1.50 (s, 3H), 2.55 (dd, *J* = 18.8 and 2.5 Hz, 1H), 3.10 (ddd, *J* = 18.7, 8.6 and 2.1 Hz, 1H), 3.65 (d, *J* = 7.9 Hz, 1H), 4.52 (s, 1H), 5.19 (d, *J* = 3.7 Hz, 1H), 5.22 (d, *J* = 3.6 Hz, 1H), 6.11 (s, 1H), 6.83 (s, 1H), 6.90 (s, 1H), 6.97-6.99 (m, 1H), 7.24-7.40 (m, 24H), 7.43-4.47 (m, 1H); HRMS (ESI-TOF) m/z: [M+NH_4_]^+^ Calcd C_47_H_45_N_2_O_7_S 781.2942, found 781.2932.

(1’*R*,2’*R*)-Benzhydryl spiro[(2-(4-fluorobenzoyl)-3-(4-methylbenzyloxycarbonyl)cyclopent-3-ene)-1’,6-penicillanate] (**14b**) and (1’*R*,2’*R*)-Benzhydryl spiro[(2-(4-fluorobenzoyl)-5-(4-methylbenzyloxycarbonyl)cyclopent-4-ene)-1’,6-penicillanate] (**15b**)

Obtained from allene **8j** (47 mg, 0.250 mmol) and 6-alkylidenepenicillanate **11a** (125 mg, 0.250 mmol) as described in the general procedure (reaction time: 7 d). Purification of the crude product by flash chromatography (hexane/ethyl acetate, 3:1), gave, in order of elution, **14b** as a colourless solid (40 mg, 0.058 mmol, 23%) and **15b** as a colourless solid (55 mg, 0.079 mmol, 32%).

Compound **14b**: mp 71.4-73.4 ºC; ${[\alpha]}_{D}^{25}$ = + 310 (c 0.5 in CH_2_Cl_2_); IR (ATR): $\tilde{\nu}$ = 986, 1000, 1103, 1155, 1201, 1331, 1450, 1508, 1593, 1671, 1711 and 1773 cm^–1^; ^1^H NMR (400 MHz, CDCl_3_): δ = 1.11 (s, 3H), 1.50 (s, 3H), 2.35 (s, 3H), 3.10 (dd, *J* = 18.6 and 3.1 Hz, 1H), 3.52 (dt, *J* = 18.5 and 2.2 Hz, 1H), 4.52 (s, 1H), 4.91 (d, *J* = 12.0 Hz, 1H), 4.96 (d, *J* = 12.0 Hz, 1H), 5.09 (d, *J* = 1.1 Hz, 1H), 5.41 (s, 1H), 6.92 (s, 1H), 6.98-7.10 (m, 7H), 7.29-7.35 (m, 10H), 8.06 (dd, *J* = 5.4 and 8.9 Hz, 2H); ^13^C NMR (100 MHz, CDCl_3_): δ = 21.3, 26.1, 32.4, 40.8, 52.8, 64.3, 66.8, 69.1, 70.7, 71.1, 78.5, 115.5 (d, *J* = 21.9 Hz, 2C), 127.1, 127.7, 128.3, 128.5, 128.7, 128.8, 128.8, 129.3, 132.0 (d, *J* = 9.5 Hz, 2C), 132.2, 133.9 (d, *J* = 2.8 Hz, 1C), 135.8, 138.4, 139.2, 139.3, 146.0, 162.9, 166.10 (d, *J* = 255.4 Hz, 1C), 167.0, 176.4, 199.6; ^19^F NMR (376 MHz, CDCl_3_): δ = -104.52 (s, 1F); HRMS (ESI-TOF) m/z: [M+NH_4_]^+^ Calcd C41H40FN2O6S 707.2586, found 707.2580.

Compound **15b**: mp 71.3-73.3 ºC; ${[\alpha]}_{D}^{25}$ = + 330 (c 0.5 in CH_2_Cl_2_); IR (ATR): $\tilde{\nu}$ = 981, 1015, 1122, 1210, 1330, 1508, 1595, 1676, 1716 and 1764 cm^–1^; ^1^H NMR (400 MHz, CDCl_3_): δ = 1.11 (s, 3H), 1.51 (s, 3H), 2.34 (s, 3H), 2.48 (dd, *J* = 18.6 and 2.5 Hz, 1H), 3.52 (ddd, *J* = 18.6, 9.1 and 2.0 Hz, 1H), 4.50 (d, *J* = 8.5 Hz, 1H), 4.54 (s, 1H), 5.16 (d, *J* = 12.4 Hz, 1H), 5.19 (d, *J* = 12.3 Hz, 1H), 6.28 (s, 1H), 6.88 (t, *J* = 2.5 Hz, 1H), 6.95 (s, 1H), 7.13-7.18 (m, 4H), 7.25-7.36 (m, 8H), 7.42 (d, *J* = 7.1 Hz, 2H), 7.47 (d, *J* = 7.3 Hz, 2H), 7.96 (dd, *J* = 8.8 and 5.3 Hz, 2H); ^13^C NMR (100 MHz, CDCl_3_): δ = 21.4, 25.9, 32.8, 35.9, 49.4, 62.7, 66.5, 69.1, 71.0, 74.0, 78.4, 116.2 (d, *J* = 22.0 Hz, 2C), 127.5, 127.5, 128.2, 128.2, 128.7, 128.7, 129.4, 131.2 (d, *J* = 9.4 Hz, 2C), 131.6 (d, *J* = 2.7 Hz, 1C), 132.8, 134.5, 138.2, 139.7, 144.6, 162.5, 166.10 (d, *J* = 256.0 Hz, 1C), 166.8, 174.3, 196.9; ^19^F NMR (376 MHz, CDCl_3_): δ = -104.22 (s, 1F); HRMS (ESI-TOF) m/z: [M+NH_4_]^+^ Calcd C_41_H_40_FN_2_O_6_S 707.2586, found 707.2579.

(1’*R*,2’*R*)-Benzhydryl spiro[(2-(4-fluorobenzoyl)-3-cinnamyloxycarbonylcyclopent-3-ene)-1’,6-penicillanate] (**14c**) and (1’*R*,2’*R*)-Benzhydryl spiro[(2-(4-fluorobenzoyl)-5-cinnamyloxycarbonylcyclopent-4-ene)-1’,6-penicillanate] (**15c**)

Obtained from allene **8n** (50 mg, 0.250 mmol) and 6-alkylidenepenicillanate **11a** (125 mg, 0.250 mmol) as described in the general procedure (reaction time: 4 d). Purification of the crude product by flash chromatography (hexane/ethyl acetate, 4:1), gave, in order of elution, **14c** as a colourless solid (28 mg, 0.040 mmol, 16%) and **15c** as a colourless solid (23 mg, 0.033 mmol, 13%).

Compound **14c**: mp 76.5-78.5 ºC; ${[\alpha]}_{D}^{25}$ = + 300 (c 0.25 in CH_2_Cl_2_); IR (ATR): $\tilde{\nu}$ = 986, 1060, 1155, 1201, 1235, 1330, 1449, 1593, 1670, 1716 and 1773 cm^–1^; ^1^H NMR (400 MHz, CDCl_3_): δ = 1.12 (s, 3H), 1.51 (s, 3H), 3.12 (dd, *J* = 18.6 and 3.1 Hz, 1H), 3.54 (dt, *J* = 2.2 and 18.5 Hz, 1H), 4.53 (s, 1H), 4.58 (ddd, *J* = 12.7, 6.8 and 1.1 Hz, 1H), 4.64 (ddd, *J* = 12.6, 6.6 and 1.1 Hz, 1H), 5.11 (d, *J* = 1.1 Hz, 1H), 5.42 (s, 1H), 6.01 (dt, *J* = 15.9 and 6.7 Hz, 1H), 6.50 (d, *J* = 15.8 Hz, 1H), 6.92 (s, 1H), 7.08 (t, *J* = 8.6 Hz, 3H), 7.31-7.35 (m, 15H), 8.13 (dd, *J* = 8.8 and 5.4 Hz, 2H); ^13^C NMR (100 MHz, CDCl_3_): δ = 26.1, 32.5, 40.8, 53.0, 64.3, 65.6, 69.1, 70.7, 71.2, 78.5, 115.6 (d, *J* = 21.9 Hz, 2C), 122.5, 126.8, 127.1, 127.7, 128.3, 128.6, 128.8, 131.2 (d, *J* = 9.5 Hz, 2C), 134.0 (d, *J* = 2.6 Hz, 1C), 134.9, 135.8, 136.1, 139.2, 139.3, 146.0, 162.8, 166.2 (d, *J* = 255.6 Hz, 1C), 167.0, 176.4, 199.6; ^19^F NMR (376 MHz, CDCl_3_): δ = -104.34 (s, 1F); HRMS (ESI-TOF) m/z: [M+NH_4_]^+^ Calcd C_42_H_40_FN_2_O_6_S 719.2586, found 719.2581.

Compound **15c**: mp 79.3-81.3 ºC; ${[\alpha]}_{D}^{25}$ = + 350 (c 0.5 in CH_2_Cl_2_); IR (ATR): $\tilde{\nu}$ = 982, 1124, 1152, 1211, 1235, 1263, 1331, 1449, 1596, 1677, 1716 and 1764 cm^–1^; ^1^H NMR (400 MHz, CDCl_3_): δ = 1.11 (s, 3H), 1.51 (s, 3H), 2.50 (dd, *J* = 18.8 and 2.8 Hz, 1H), 3.20 (ddd, *J* = 18.6, 9.1 and 2.0 Hz, 1H), 4.51 (d, *J* = 8.5 Hz, 1H), 4.55 (s, 1H), 4.83 (dd, *J* = 6.5 and 1.0 Hz, 2H), 6.28 (s, 1H), 6.33 (dt, *J* = 15.9 and 6.5 Hz, 1H), 6.65 (d, *J* = 15.9 Hz, 1H), 6.92-6.93 (m, 2H), 7.16 (t, *J* = 8.6 Hz, 2H), 7.25-7.41 (m, 13H), 7.45 (d, *J* = 7.3 Hz, 2H), 7.97 (dd, *J* = 8.8 and 5.3 Hz, 2H); ^13^C NMR (100 MHz, CDCl_3_): δ = 25.9, 32.8, 36.0, 49.5, 62.8, 66.6, 69.1, 71.0, 74.0, 78.4, 116.3 (d, *J* = 21.9 Hz, 2C), 123.2, 126.9, 127.4, 127.6, 128.2, 128.2, 128.7, 128.7, 131.2 (d, *J* = 9.3 Hz, 2C), 131.6 (d, *J* = 2.8 Hz, 1C), 134.5, 134.6, 136.4, 139.6, 144.6, 162.5, 166.1 (d, *J* = 256.0 Hz, 1C), 166.8, 174.2, 196.9; ^19^F NMR (376 MHz, CDCl_3_): δ = -104.20 (s, 1F); HRMS (ESI-TOF) m/z: [M+NH_4_]^+^ Calcd C_42_H_40_FN_2_O_6_S 719.2586, found 719.2582.

(1’*R*,2’*R*)-Benzhydryl spiro[(2-(4-chlorobenzoyl)-3-(4-methoxybenzyloxycarbonyl)cyclopent-3-ene)-1’,6-penicillanate] (**14d**) and (1’*R*,2’*R*)-Benzhydryl spiro[(2-(4-chlorobenzoyl)-5-(4-methoxybenzyloxycarbonyl)cyclopent-4-ene)-1’,6-penicillanate] (**15d**)

Obtained from allene **8i** (51 mg, 0.250 mmol) and 6-alkylidenepenicillanate **11b** (130 mg, 0.250 mmol) as described in the general procedure (reaction time: 22.5 h). Purification of the crude product by flash chromatography (hexane/ethyl acetate, 4:1), gave, in order of elution, **14d** as a colourless solid (77 mg, 0.106 mmol, 42%) and **15d** as a colourless solid (65 mg, 0.090 mmol, 36%).

Compound **14d**: mp 76.1-78.1 ºC; ${[\alpha]}_{D}^{25}$ = +260 (c 0.5 in CH_2_Cl_2_); IR (ATR): $\tilde{\nu}$ = 999, 1092, 1175, 1244, 1331, 1457, 1514, 1587, 1671, 1708, 1742 and 1773 cm^–1^; ^1^H NMR (400 MHz, CDCl_3_): δ = 1.11 (s, 3H), 1.49 (s, 3H), 3.10 (dd, *J* = 18.6 and 3.1 Hz, 1H), 3.17 (dt, *J* = 18.6 and 2.3 Hz, 1H), 3.81 (s, 3H), 4.52 (s, 1H), 4.88 (d, *J* = 11.9 Hz, 1H), 4.93 (d, *J* = 11.9 Hz, 1H), 5.07 (d, *J* = 1.0 Hz, 1H), 5.39 (s, 1H), 6.80-6.82 (m, 2H), 6.91 (s, 1H), 7.05-7.07 (m, 3H), 7.26-7.34 (m, 12H), 7.96 (d, *J* = 8.6 Hz, 2H); ^13^C NMR (100 MHz, CDCl_3_): δ = 26.1, 32.5, 40.7, 52.8, 55.4, 64.4, 66.7, 69.1, 70.8, 71.1, 78.5, 114.0, 127.1, 127.3, 127.7, 128.4, 128.5, 128.7, 130.5, 130.7, 135.8, 135.8, 139.2, 139.3, 140.0, 146.1, 159.9, 162.9, 166.9, 176.3, 200.1; HRMS (ESI-TOF) m/z: [M+NH_4_]^+^ Calcd C_41_H_40_ClN_2_O_7_S 739.2239, found 739.2235.

Compound **15d**: mp 83.8-85.8 ºC; ${[\alpha]}_{D}^{25}$ = + 350 (c 0.5 in CH_2_Cl_2_); IR (ATR): $\tilde{\nu}$ = 982, 1011, 1090, 1123, 1248, 1330, 1513, 1587, 1676, 1712 and 1763 cm^–1^; ^1^H NMR (400 MHz, CDCl_3_): δ = 1.11 (s, 3H), 1.51 (s, 3H), 2.46 (dd, *J* = 18.7 and 2.8 Hz, 1H), 3.17 (ddd, *J* = 18.5, 9.1 and 2.0 Hz, 1H), 3.80 (s, 3H), 4.48 (d, *J* = 8.6 Hz, 1H), 4.54 (s, 1H), 5.13 (d, *J* = 12.0 Hz, 1H), 5.17 (d, *J* = 12.0 Hz, 1H), 6.26 (s, 1H), 6.85-6.87 (m, 3H), 6.95 (s, 1H), 7.27-7.36 (m, 8H), 7.42-7.43 (m, 2H), 7.45-7.48 (m, 3H), 7.87 (d, *J* = 8.6 Hz, 1H); ^13^C NMR (100 MHz, CDCl_3_): δ = 25.9, 32.8, 35.8, 49.4, 55.4, 62.8, 66.4, 69.1, 71.0, 74.0, 78.4, 114.1, 127.5, 127.5, 128.0, 128.2, 128.3, 128.7, 128.7, 129.4, 129.9, 130.4, 133.5, 134.5, 139.6, 140.2, 144.5, 159.8, 162.5, 166.8, 174.2, 197.2; HRMS (ESI-TOF) m/z: [M+NH_4_]^+^ Calcd C_41_H_40_ClN_2_O_7_S 739.2239, found 739.2236.

(1’*R*,2’*R*)-Benzhydryl spiro[(2-(4-chlorobenzoyl)-3-(4-methylbenzyloxycarbonyl)cyclopent-3-ene)-1’,6-penicillanate] (**14e**) and (1’*R*,2’*R*)-Benzhydryl spiro[(2-(4-chlorobenzoyl)-5-(4-methylbenzyloxycarbonyl)cyclopent-4-ene)-1’,6-penicillanate] (**15e**)

Obtained from allene **8j** (37 mg, 0.197 mmol) and 6-alkylidenepenicillanate **11b** (102 mg, 0.197 mmol) as described in the general procedure (reaction time: 67 h). Purification of the crude product by flash chromatography (hexane/ethyl acetate, 4:1), gave, in order of elution, **14e** as a colourless solid (39 mg, 0.055 mmol, 28%) and **15e** as a colourless solid (38 mg, 0.054 mmol, 27%).

Compound **14e**: mp 79.5-81.5 ºC; ${[\alpha]}_{D}^{25}$ = + 340 (c 0.25 in CH_2_Cl_2_); IR (ATR): $\tilde{\nu}$ = 999, 1092, 1179, 1202, 1237, 1262, 1331, 1457, 1588, 1671, 1713 and 1774 cm^–1^; ^1^H NMR (400 MHz, CDCl_3_): δ = 1.11 (s, 3H), 1.49 (s, 3H), 2.35 (s, 3H), 3.10 (dd, *J* = 18.6 and 3.1 Hz, 1H), 3.51 (dt, *J* = 18.5 and 2.2 Hz, 1H), 4.52 (s, 1H), 4.90 (d, *J* = 12.0 Hz, 1H), 4.95 (d, *J* = 12.0 Hz, 1H), 5.07 (s, 1H), 5.39 (s, 1H), 6.91 (s, 1H), 7.02 (d, *J* = 8.0 Hz, 2H), 7.06 (s, 1H), 7.10 (d, *J* = 7.9 Hz, 2H), 7.28-7.34 (m, 12H), 7.95 (d, *J* = 8.7 Hz, 2H); ^13^C NMR (100 MHz, CDCl_3_): δ = 21.4, 26.1, 32.5, 40.7, 52.8, 64.4, 66.9, 69.1, 70.8, 71.1, 78.5, 127.1, 127.7, 128.4, 128.6, 128.7, 128.8, 128.8, 129.3, 130.7, 132.1, 135.7, 135.8, 138.4, 139.2, 139.3, 140.1, 146.1, 162.9, 166.9, 176.3, 200.1; HRMS (ESI-TOF) m/z: [M+NH_4_]^+^ Calcd C_41_H_40_ClN_2_O_6_S 723.2290, found 723.2287.

Compound **15e**: mp 81.0-83.0 ºC; ${[\alpha]}_{D}^{25}$ = + 360 (c 0.25 in CH_2_Cl_2_); IR (ATR): $\tilde{\nu}$ = 982, 1012, 1091, 1123, 1210, 1261, 1331, 1456, 1588, 1676, 1716 and 1767 cm^–1^; 1H NMR (400 MHz, CDCl3): δ = 1.11 (s, 3H), 1.51 (s, 3H), 2.34 (s, 3H), 2.46 (dd, *J* = 18.6 and 2.4 Hz, 1H), 3.17 (ddd, *J* = 18.5, 9.1 and 2.0 Hz, 1H), 4.48 (d, *J* = 8.5 Hz, 1H), 4.54 (s, 1H), 5.17 (s, 2H), 6.26 (s, 1H), 6.88 (t, *J* = 2.5, 1H), 6.94 (s, 1H), 7.14 (d, *J* = 7.9 Hz, 2H), 7.25-7.35 (m, 10H), 7.41-7.47 (m, 6H), 7.87 (d, *J* = 8.6 Hz, 2H); ^13^C NMR (100 MHz, CDCl_3_): δ = 21.4, 25.9, 32.8, 35.9, 49.4, 62.8, 66.6, 69.1, 71.0, 74.1, 78.4, 127.5, 127.5, 128.2, 128.3, 128.7, 128.7, 129.4, 129.4, 129.9, 132.8, 133.5, 134.5, 138.2, 139.6, 140.2, 144.5, 162.5, 166.8, 174.2, 197.3; HRMS (ESI-TOF) m/z: [M+NH_4_]^+^ Calcd C_41_H_40_ClN_2_O_6_S 723.2290, found 723.2291.

(1’*R*,2’*R*)-Benzhydryl spiro[(2-(4-chlorobenzoyl)-3-cinnamyloxycarbonylcyclopent-3-ene)-1’,6-penicillanate] (**14f**) and (1’*R*,2’*R*)-Benzhydryl spiro[(2-(4-chlorobenzoyl)-5-cinnamyloxycarbonylcyclopent-4-ene)-1’,6-penicillanate] (**15f**)

Obtained from allene **8n** (50 mg, 0.250 mmol) and 6-alkylidenepenicillanate **11b** (130 mg, 0.250 mmol) as described in the general procedure (reaction time: 4 d). Purification of the crude product by flash chromatography (hexane/ethyl acetate, 3:1), gave, in order of elution, **14f** as a colourless solid (34 mg, 0.047 mmol, 19%) and **15f** as a colourless solid (31 mg, 0.043 mmol, 17%).

Compound **14f**: mp 81.7-83.7 ºC; ${[\alpha]}_{D}^{25}$ = + 280 (c 0.25 in CH_2_Cl_2_); IR (ATR): $\tilde{\nu}$ = 999, 1092, 1176, 1200, 1236, 1261, 1330, 1457, 1588, 1671, 1716 and 1773 cm^–1^; ^1^H NMR (400 MHz, CDCl_3_): δ = 1.12 (s, 3H), 1.51 (s, 3H), 3.12 (dd, *J* = 18.6 and 3.1 Hz, 1H), 3.53 (dt, *J* = 18.5 and 2.2 Hz, 1H), 4.53 (s, 1H), 4.57 (ddd, *J* = 12.7, 6.7 and 1.1 Hz, 1H), 4.64 (ddd, *J* = 12.6, 6.7 and 1.1 Hz, 1H), 5.10 (d, *J* = 1.1 Hz, 1H), 5.42 (s, 1H), 6.00 (dt, *J* = 15.8 and 6.7 Hz, 1H), 6.50 (d, *J* = 15.9 Hz, 1H), 6.91 (s, 1H), 7.08 (s, 1H), 7.29-7.38 (m, 15H), 7.39 (d, *J* = 8.6 Hz, 2H), 8.04 (d, *J* = 8.7 Hz, 2H); ^13^C NMR (100 MHz, CDCl_3_): δ = 26.1, 32.6, 40.8, 52.9, 64.4, 65.7, 69.1, 70.8, 71.2, 78.5, 122.5, 126.8, 127.1, 127.7, 128.4, 128.6, 128.8, 128.8, 129.0, 130.8, 135.0, 135.8, 135.9, 136.1 139.2, 139.3, 140.2, 146.0, 162.8, 166.9, 176.3, 200.1; HRMS (ESI-TOF) m/z: [M+NH_4_]^+^ Calcd C_42_H_40_ClN_2_O_6_S 735.2290, found 735.2287.

Compound **15f**: mp 85.5-87.5 ºC; ${[\alpha]}_{D}^{25}$ = + 320 (c 0.25 in CH_2_Cl_2_); IR (ATR): $\tilde{\nu}$ = 983, 1012, 1091, 1126, 1210, 1261, 1331, 1449, 1492, 1589, 1677, 1716 and 1767 cm^–1^; ^1^H NMR (400 MHz, CDCl_3_): δ = 1.10 (s, 3H), 1.51 (s, 3H), 2.49 (dd, *J* = 18.7 and 3.0 Hz, 1H), 3.20 (ddd, *J* = 18.6, 9.1 and 2.0 Hz, 1H), 4.49 (d, *J* = 8.5 Hz, 1H), 4.55 (s, 1H), 4.83 (dd, *J* = 6.5 and 1.0 Hz, 2H), 6.27 (s, 1H), 6.33 (dt, *J* = 15.9 and 6.5 Hz, 1H), 6.65 (d, *J* = 15.9 Hz, 1H), 6.92 (s, 2H), 7.25-7.40 (m, 13H), 7.46 (d, *J* = 7.8 Hz, 4H), 7.88 (d, *J* = 8.6 Hz, 2H); ^13^C NMR (100 MHz, CDCl_3_): δ = 25.9, 32.9, 35.9, 49.4, 62.8, 65.6, 69.1, 71.1, 74.1, 78.4, 123.2, 126.9, 127.4, 127.6, 128.2, 128.7, 128.7, 129.4, 130.0, 133.6, 134.5, 134.6, 136.3, 139.6, 139.6, 140.3, 144.5, 162.5, 166.8, 174.1, 197.3; HRMS (ESI-TOF) m/z: [M+NH_4_]^+^ Calcd C_42_H_40_ClN_2_O_6_S 735.2290, found 735.2288.

(1’*R*,2’*R*)-Benzhydryl spiro[(2-(4-bromobenzoyl)-3-(4-methoxybenzyloxycarbonyl)cyclopent-3-ene)-1’,6-penicillanate] (**14g**) and (1’*R*,2’*R*)-Benzhydryl spiro[(2-(4-bromobenzoyl)-5-(4-methoxybenzyloxycarbonyl)cyclopent-4-ene)-1’,6-penicillanate] (**15g**)

Obtained from allene **8i** (51 mg, 0.250 mmol) and 6-alkylidenepenicillanate **11c** (141 mg, 0.250 mmol) as described in the general procedure (reaction time: 24 h). Purification of the crude product by flash chromatography (hexane/ethyl acetate, 4:1), gave, in order of elution, **14g** as a colourless solid (81 mg, 0.105 mmol, 42%) and **15g** as a colourless solid (69 mg, 0.090 mmol, 36%).

Compound **14g**: mp 82.6-84.6 ºC; ${[\alpha]}_{D}^{25}$ = + 320 (c 0.25 in CH_2_Cl_2_); IR (ATR): $\tilde{\nu}$ = 998, 1069, 1175, 1243, 1331, 1458, 1584, 1671, 1708 and 1774 cm^–1^; ^1^H NMR (400 MHz, CDCl_3_): δ = 1.11 (s, 3H), 1.49 (s, 3H), 3.10 (dd, *J* = 18.6 and 3.1 Hz, 1H), 3.50 (dt, *J* = 18.6 and 2.3 Hz, 1H), 3.82 (s, 3H), 4.52 (s, 1H), 4.88 (d, *J* = 11.9 Hz, 1H), 4.93 (d, *J* = 11.9 Hz, 1H), 5.06 (d, *J* = 1.1 Hz, 1H), 5.39 (s, 1H), 6.82 (d, *J* = 8.7 Hz, 2H), 6.91 (s, 1H), 7.05-7.07 (m, 3H), 7.29-7.34 (m, 10H), 7.48 (d, *J* = 8.6 Hz, 2H), 7.88 (d, *J* = 8.6 Hz, 2H); ^13^C NMR (100 MHz, CDCl_3_): δ = 26.1, 32.6, 40.7, 52.7, 55.4, 64.4, 66.8, 69.1, 70.8, 71.1, 78.5, 114.0, 127.1, 127.3, 127.7, 128.4, 128.6, 128.8, 128.9, 130.5, 130.8, 131.7, 135.8, 136.2, 139.2, 139.3, 146.1, 159.9, 162.9, 166.9, 176.3, 200.3; HRMS (ESI-TOF) m/z: [M+H]^+^ Calcd C_41_H_37_BrNO_7_S 766.1469, found 766.1462.

Compound **15g**: mp 86.5-88.5 ºC; ${[\alpha]}_{D}^{25}$ = + 320 (c 0.5 in CH_2_Cl_2_); IR (ATR): $\tilde{\nu}$ = 983, 1009, 1071, 1123, 1249, 1331, 1514, 1584, 1676, 1715 and 1766 cm^–1^; ^1^H NMR (400 MHz, CDCl_3_): δ = 1.11 (s, 3H), 1.51 (s, 3H), 2.45 (dd, *J* = 18.7 and 2.5 Hz, 1H), 3.16 (ddd, *J* = 18.5, 9.1 and 2.0 Hz, 1H), 3.79 (s, 3H), 4.47 (d, *J* = 8.5 Hz, 1H), 4.54 (s, 1H), 5.12 (d, *J* = 12.0 Hz, 1H), 5.17 (d, *J* = 12.0 Hz, 1H), 6.25 (s, 1H), 6.85-6.87 (m, 3H), 6.95 (s, 1H), 7.27-7.36 (m, 8H), 7.41-7.43 (m, 2H), 7.46-7.48 (m, 2H), 7.63 (d, *J* = 8.6 Hz, 1H), 7.79 (d, *J* = 8.6 Hz, 1H); ^13^C NMR (100 MHz, CDCl_3_): δ = 25.9, 32.8, 35.8, 49.4, 55.4, 62.8, 66.4, 69.1, 71.0, 74.1, 78.4, 114.1, 127.5, 127.5, 128.0, 128.2, 128.3, 128.7, 128.7, 129.0, 130.0, 130.4, 132.4, 134.0, 134.5, 139.6, 144.5, 159.8, 162.5, 166.8, 174.2, 197.4; HRMS (ESI-TOF) m/z: [M+H]+ Calcd C_41_H_37_BrNO_7_S 766.1469, found 766.1463.

(1’*R*,2’*R*)-Benzhydryl spiro[(2-(4-bromobenzoyl)-3-(4-methylbenzyloxycarbonyl)cyclopent-3-ene)-1’,6-penicillanate] (**14h**) and (1’*R*,2’*R*)-Benzhydryl spiro[(2-(4-bromobenzoyl)-5-(4-methylbenzyloxycarbonyl)cyclopent-4-ene)-1’,6-penicillanate] (**15h**)

Obtained from allene **8j** (47 mg, 0.250 mmol) and 6-alkylidenepenicillanate **11c** (141 mg, 0.250 mmol) as described in the general procedure (reaction time: 4 d). Purification of the crude product by flash chromatography (hexane/ethyl acetate, 4:1), gave, in order of elution, **14h** as a colourless solid (46 mg, 0.061 mmol, 25%) and **15h** as a colourless solid (43 mg, 0.057 mmol, 23%).

Compound **14h**: mp 74.9-76.9 ºC; ${[\alpha]}_{D}^{25}$ = + 270 (c 0.5 in CH_2_Cl_2_); IR (ATR): $\tilde{\nu}$ = 998, 1069, 1177, 1201, 1237, 1262, 1331, 1458, 1583, 1671, 1712 and 1774 cm^–1^; ^1^H NMR (400 MHz, CDCl_3_): δ = 1.11 (s, 3H), 1.49 (s, 3H), 2.36 (s, 3H), 3.10 (dd, *J* = 18.6 and 3.1 Hz, 1H), 3.51 (dt, *J* = 18.6 and 2.3 Hz, 1H), 4.52 (s, 1H), 4.91 (d, *J* = 12.0 Hz, 1H), 4.95 (d, *J* = 12.0 Hz, 1H), 5.07 (d, *J* = 1.1 Hz, 1H), 5.39 (s, 1H), 6.91 (s, 1H), 7.02 (d, *J* = 8.0 Hz, 2H), 7.07 (s, 1H), 7.11 (d, *J* = 7.9 Hz, 2H), 7.29-7.35 (m, 10H), 7.46 (d, *J* = 8.6 Hz, 2H), 7.88 (d, *J* = 8.6 Hz, 2H); ^13^C NMR (100 MHz, CDCl_3_): δ = 21.4, 26.1, 32.5, 40.7, 52.7, 64.4, 66.9, 69.1, 70.8, 71.1, 78.5, 127.1, 127.7, 128.4, 128.5, 128.7, 128.8, 128.8, 129.0, 129.4, 130.8, 131.7, 132.1, 135.8, 136.1, 138.4, 139.2, 139.3, 146.2, 162.9, 166.9, 176.3, 200.2; HRMS (ESI-TOF) m/z: [M+NH_4_]^+^ Calcd C_41_H_40_BrN_2_O_6_S 767.1785, found 767.1782.

Compound **15h**: mp 76.7-78.7 ºC; ${[\alpha]}_{D}^{25}$ = + 400 (c 0.25 in CH_2_Cl_2_); IR (ATR): $\tilde{\nu}$ = 982, 1008, 1070, 1121, 1256, 1330, 1449, 1583, 1676, 1716 and 1763 cm^–1^; ^1^H NMR (400 MHz, CDCl_3_): δ = 1.11 (s, 3H), 1.51 (s, 3H), 2.34 (s, 3H), 2.46 (dd, *J* = 18.7 and 2.5 Hz, 1H), 3.17 (ddd, *J* = 18.5, 9.1 and 2.0 Hz, 1H), 4.48 (d, *J* = 8.5 Hz, 1H), 4.54 (s, 1H), 5.17 (s, 2H), 6.26 (s, 1H), 6.88 (dd, *J* = 2.9 and 2.2 Hz, 1H), 6.94 (s, 1H), 7.14 (d, *J* = 7.9 Hz, 2H), 7.25-7.35 (m, 10H), 7.42 (d, *J* = 7.1 Hz, 2H), 7.47 (d, *J* = 7.2 Hz, 2H), 7.63 (d, *J* = 8.6 Hz, 2H), 7.79 (d, *J* = 8.6 Hz, 2H); ^13^C NMR (100 MHz, CDCl_3_): δ = 21.4, 25.9, 32.9, 35.8, 49.4, 62.8, 66.6, 69.1, 71.0, 74.1, 78.4, 127.5, 127.5, 128.2, 128.3, 128.7, 128.7, 129.0, 129.4, 130.0, 132.4, 132.8, 134.0, 134.5, 138.2, 139.6, 144.5, 162.5, 166.7, 174.2, 197.4; HRMS (ESI-TOF) m/z: [M+NH_4_]^+^ Calcd C_41_H_40_BrN_2_O_6_S 767.1785, found 767.1777.

(1’*R*,2’*R*)-Benzhydryl spiro[(2-(4-bromobenzoyl)-3-cinnamyloxycarbonylcyclopent-3-ene)-1’,6-penicillanate] (**14i**) and (1’*R*,2’*R*)-Benzhydryl spiro[(2-(4-bromobenzoyl)-5-cinnamyloxycarbonylcyclopent-4-ene)-1’,6-penicillanate] (**15i**)

Obtained from allene **8n** (50 mg, 0.250 mmol) and 6-alkylidenepenicillanate **11c** (141 mg, 0.250 mmol) as described in the general procedure (reaction time: 4 d). Purification of the crude product by flash chromatography (hexane/ethyl acetate, 3:1), gave, in order of elution, **14i** as a colourless solid (38 mg, 0.050 mmol, 20%) and **15i** as a colourless solid (31 mg, 0.040 mmol, 16%).

Compound **14i**: mp 89.1-91.1 ºC; ${[\alpha]}_{D}^{25}$ = + 360 (c 0.25 in CH_2_Cl_2_); IR (ATR): $\tilde{\nu}$ = 998, 1070, 1177, 1201, 1236, 1261, 1330, 1449, 1583, 1670, 1716 and 1773 cm^–1^; ^1^H NMR (400 MHz, CDCl_3_): δ = 1.12 (s, 3H), 1.51 (s, 3H), 3.12 (dd, *J* = 18.6 and 3.1 Hz, 1H), 3.53 (dt, *J* = 18.6 and 2.2 Hz, 1H), 4.53 (s, 1H), 4.58 (ddd, *J* = 12.8, 6.9 and 1.1 Hz, 1H), 4.64 (ddd, *J* = 12.6, 6.6 and 1.1 Hz, 1H), 5.10 (d, *J* = 1.0 Hz, 1H), 5.41 (s, 1H), 6.01 (dt, *J* = 15.8 and 6.7 Hz, 1H), 6.50 (d, *J* = 15.9 Hz, 1H), 6.92 (s, 1H), 7.08 (s, 1H), 7.29-7.35 (m, 15H), 7.57 (d, *J* = 8.6 Hz, 2H), 7.96 (d, *J* = 8.7 Hz, 2H); ^13^C NMR (100 MHz, CDCl_3_): δ = 26.1, 32.6, 40.8, 52.9, 64.4, 65.7, 69.1, 70.8, 71.2, 78.5, 122.5, 126.8, 127.1, 127.7, 128.4, 128.6, 128.8, 128.8, 129.0, 130.9, 131.8, 135.0, 135.8, 136.0, 136.3 139.2, 139.3, 146.1, 162.8, 166.9, 176.3, 200.3; HRMS (ESI-TOF) m/z: [M+NH_4_]^+^ Calcd C_42_H_40_BrN_2_O_6_S 779.1785, found 781.1761.

Compound **15i**: mp 91.2-93.2 ºC; ${[\alpha]}_{D}^{25}$ = + 400 (c 0.25 in CH_2_Cl_2_); IR (ATR): $\tilde{\nu}$ = 983, 1010, 1126, 1209, 1261, 1331, 1449, 1584, 1676, 1718 and 1767 cm^–1^; ^1^H NMR (400 MHz, CDCl_3_): δ = 1.10 (s, 3H), 1.51 (s, 3H), 2.49 (dd, *J* = 18.6 and 3.0 Hz, 1H), 3.19 (ddd, *J* = 18.6, 9.0 and 2.0 Hz, 1H), 4.49 (d, *J* = 8.5 Hz, 1H), 4.55 (s, 1H), 4.83 (dd, *J* = 6.5 and 1.0 Hz, 2H), 6.27 (s, 1H), 6.33 (dt, *J* = 15.9 and 6.5 Hz, 1H), 6.65 (d, *J* = 15.9 Hz, 1H), 6.92 (s, 2H), 7.25-7.40 (m, 9H), 7.46 (d, *J* = 7.2 Hz, 4H), 7.46 (d, *J* = 8.6 Hz, 2H), 7.80 (d, *J* = 8.6 Hz, 2H); ^13^C NMR (100 MHz, CDCl_3_): δ = 25.9, 32.9, 35.8, 49.4, 62.9, 65.6, 69.1, 71.1, 74.1, 78.4, 123.2, 126.9, 127.4, 127.6, 128.2, 128.7, 128.7, 129.0, 130.0, 131.8, 132.4, 134.0, 134.5, 134.7, 136.3, 139.6, 144.5, 162.5, 166.8, 174.1, 197.5; HRMS (ESI-TOF) m/z: [M+NH_4_]^+^ Calcd C_42_H_40_BrN_2_O_6_S 779.1785, found 779.1782.

**References:**

1. Mo, D.-L.; Wink, D. J.; Anderson, L. L. Solvent-Controlled Bifurcated Cascade Process for the Selective Preparation of Dihydrocarbazoles or Dihydropyridoindoles. *Chemistry – A European Journal* **2014,** 20, 13217-13225.

2. Conner, M. L.; Brown, M. K. Synthesis of 1,3-Substituted Cyclobutanes by Allenoate-Alkene [2 + 2] Cycloaddition. *The Journal of Organic Chemistry* **2016,** 81, 8050-8060.

3. Himbert, G.; Fink, D.; Diehl, K. Cycloadditionen, 12. Einfluß von Alkylgruppen am Aromaten auf die Intramolekulare Diels-Alder-Reaktion von allencarbonsäure-arylestern und Allencarboxaniliden. *Chem. Ber.*, **1988**, 121, 431-441.

4. Himbert, G.; Fink, D.; Diehl, K.; Rademacher, P.; Bittner, A.J. Cycloadditionen, 15. Einfluß von Substituenten in p-, m- und o-Position am Aromaten auf die intramolekulare Diels-Alder-Reaktion von Allencarbonsäure-aniliden und -phenylestern. *Chem. Ber.*, **1989**, 122, 1161-1173.

5. Santos, B. S.; Pinho e Melo, T. M. V. D. Synthesis of Chiral Spirocyclopentenyl-β-lactams through Phosphane-Catalyzed [3+2] Annulation of Allenoates with 6 Alkylidenepenicillanates. Eur. J. Org. Chem. 2013, 2013, 3901-3909.

**Copies of ^1^H, ^13^C NMR Spectra for New Compounds**


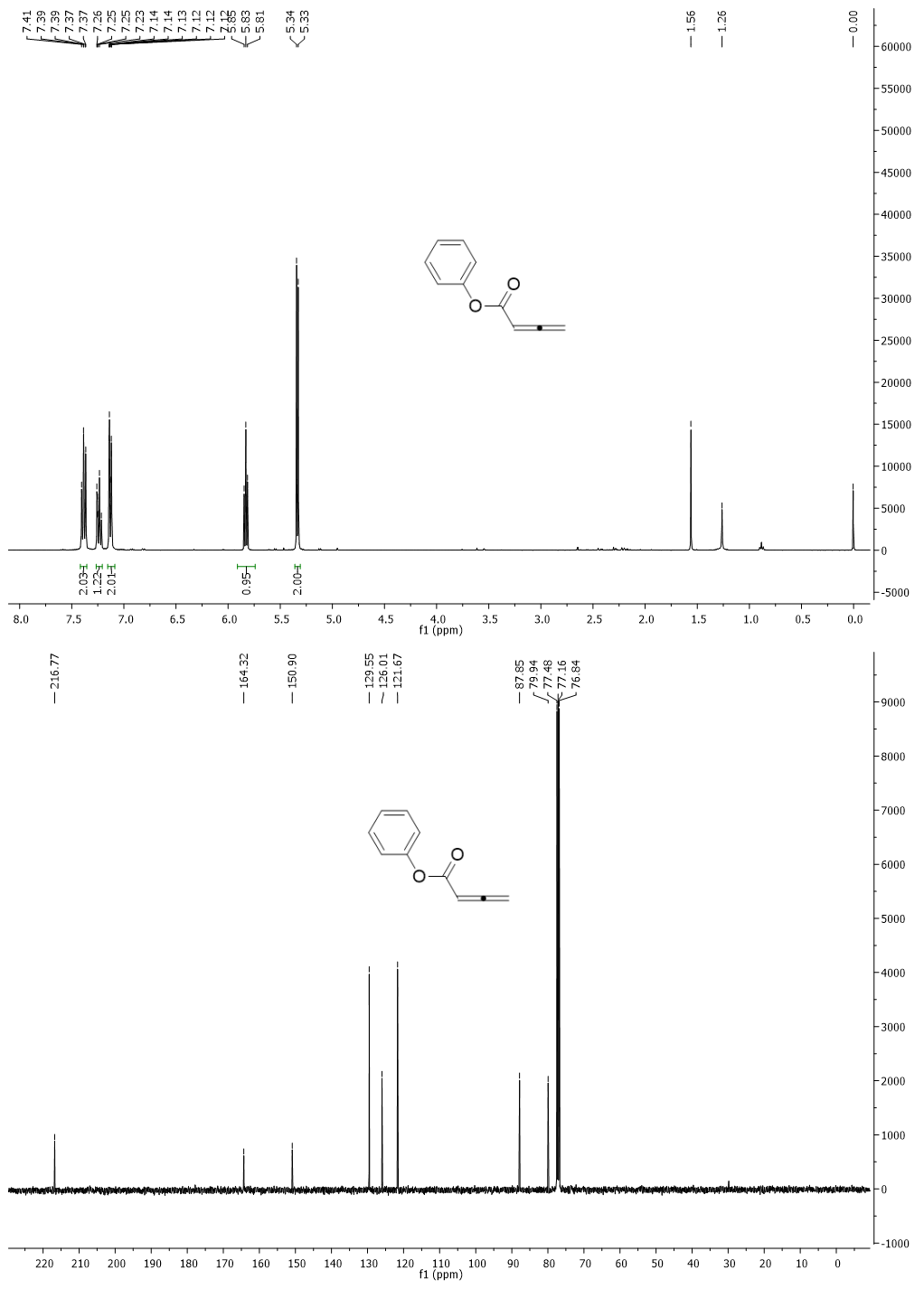


**Figure S1 -** ^1^H and ^13^C NMR spectra (CDCl_3_) of compound **8a**.


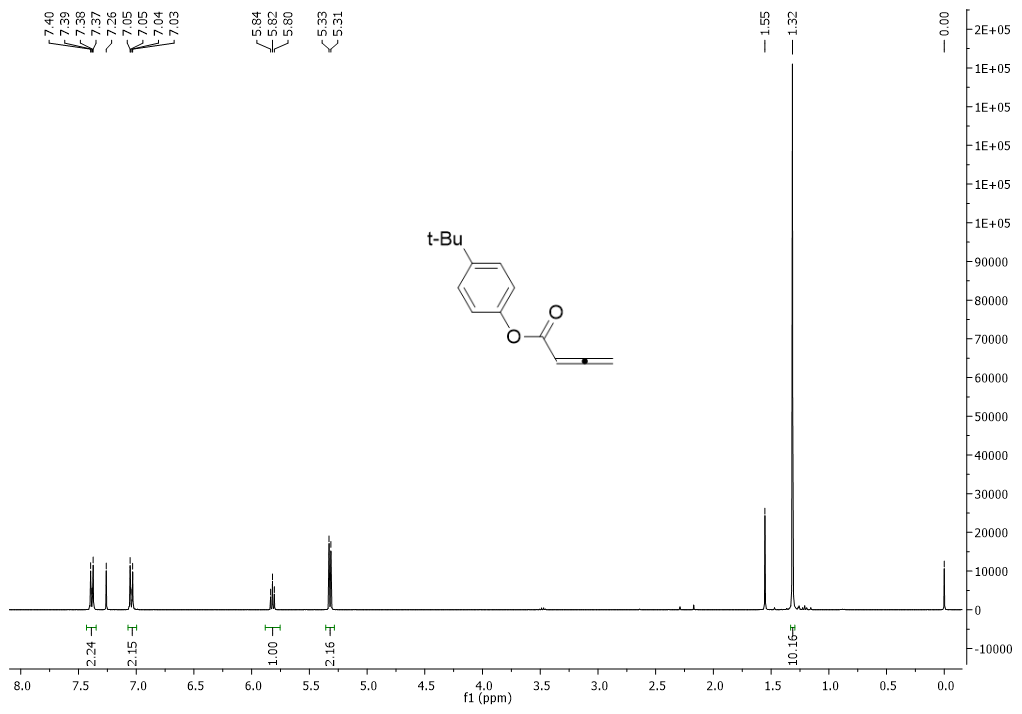

**Figure S2 -** ^1^H and ^13^C NMR spectra (CDCl_3_) of compound **8b**.

**Figure S3 -** ^1^H and ^13^C NMR spectra (CDCl_3_) of compound **8c**.

**Figure S4 -** ^1^H and ^13^C NMR spectra (CDCl_3_) of compound **8d**.

**Figure S5 -** ^1^H and ^13^C NMR spectra (CDCl_3_) of compound **8e**.

**Figure S6 -** ^1^H and ^13^C NMR spectra (CDCl_3_) of compound **8f**.

**Figure S7 -** ^1^H and ^13^C NMR spectra (CDCl_3_) of compound **8g**.

**Figure S8 -** ^1^H and ^13^C NMR spectra (CDCl_3_) of compound **8h**.

**Figure S9 -** ^1^H and ^13^C NMR spectra (CDCl_3_) of compound **8i**.

**Figure S10 -** ^1^H spectrum (CDCl_3_) of the mixture of compound **8j** and the corresponding 2-butyonate isomer.

**Figure S11 -** ^1^H spectrum (CDCl_3_) of the mixture of compound **8k** and the corresponding 2-butyonate isomer.

**Figure S12 -** ^1^H spectrum (CDCl_3_) of the mixture of compound **8l** and the corresponding 2-butyonate isomer.

**Figure S13 -** ^1^H and ^13^C NMR spectra (CDCl_3_) of compound **8m**.

**Figure S14 -** ^1^H spectrum (CDCl_3_) of the mixture of compound **8n** and the corresponding 2-butyonate isomer.

**Figure S15 -** ^1^H spectrum (CDCl_3_) of the mixture of compound **8o** and the corresponding 2-butyonate isomer.

**Figure S16 -** ^1^H and ^13^C NMR spectra (CDCl_3_) of compound **9a**.

**Figure S17 -** ^1^H and ^13^C NMR spectra (CDCl_3_) of compound **10a**.

**Figure S18 -** ^1^H and ^13^C NMR spectra (CDCl_3_) of compound **9b**.

**Figure S19 -** ^1^H and ^13^C NMR spectra (CDCl_3_) of compound **10b**.

**Figure S20 -** ^1^H and ^13^C NMR spectra (CDCl_3_) of compound **9c**.

**Figure S21 -** ^1^H and ^13^C NMR spectra (CDCl_3_) of compound **10c**.

**Figure S22 -** ^1^H and ^13^C NMR spectra (CDCl_3_) of compound **9d**.

**Figure S23 -** ^1^H and ^13^C NMR spectra (CDCl_3_) of compound **10d**.

**Figure S24 -** ^1^H and ^13^C NMR spectra (CDCl_3_) of compound **9e**.

**Figure S25 -** ^1^H and ^13^C NMR spectra (CDCl_3_) of compound **10e**.

**Figure S26 -** ^1^H and ^13^C NMR spectra (CDCl_3_) of compound **9f**.

**Figure S27 -** ^1^H and ^13^C NMR spectra (CDCl_3_) of compound **10f**.

**Figure S28 -** ^1^H and ^13^C NMR spectra (CDCl_3_) of compound **9g**.

**Figure S29 -** ^1^H and ^13^C NMR spectra (CDCl_3_) of compound **10g**.

**Figure S30 -** ^1^H and ^13^C NMR spectra (CDCl_3_) of compound **9h**.

**Figure S31 -** ^1^H and ^13^C NMR spectra (CDCl_3_) of compound **10h**.

**Figure S32 -** ^1^H and ^13^C NMR spectra (CDCl_3_) of compound **9i**.

**Figure S33 -** ^1^H and ^13^C NMR spectra (CDCl_3_) of compound **10i**.

**Figure S34 -** ^1^H and ^13^C NMR spectra (CDCl_3_) of compound **9j**.

**Figure S35 -** ^1^H and ^13^C NMR spectra (CDCl_3_) of compound **10j**.

**Figure S36 -** ^1^H and ^13^C NMR spectra (CDCl_3_) of compound **9k**.

**Figure S37 -** ^1^H and ^13^C NMR spectra (CDCl_3_) of compound **10k**.

**Figure S38 -** ^1^H and ^13^C NMR spectra (CDCl_3_) of compound **9l**.

**Figure S39 -** ^1^H and ^13^C NMR spectra (CDCl_3_) of compound **10l**.

**Figure S40 -** ^1^H and ^13^C NMR spectra (CDCl_3_) of compound **9m**.

**Figure S41 -** ^1^H and ^13^C NMR spectra (CDCl_3_) of compound **10m**.

**Figure S42 -** ^1^H and ^13^C NMR spectra (CDCl_3_) of compound **9n**.

**Figure S43 -** ^1^H and ^13^C NMR spectra (CDCl_3_) of compound **10n**.

**Figure S44 -** ^1^H and ^13^C NMR spectra (CDCl_3_) of compound **9o**.

**Figure S45 -** ^1^H and ^13^C NMR spectra (CDCl_3_) of compound **10o**.

**Figure S46 -** ^1^H and ^13^C NMR spectra (CDCl_3_) of compound **12a**.

**Figure S47 -** ^19^F NMR spectrum (CDCl_3_) of compound **12a**.

**Figure S48 -** ^1^H and ^13^C NMR spectra (CDCl_3_) of compound **13a**.

**Figure S49 -** ^19^F NMR spectrum (CDCl_3_) of compound **13a**.

**Figure S50 -** ^1^H and ^13^C NMR spectra (CDCl_3_) of compound **12b**.

**Figure S51 -** ^1^H and ^13^C NMR spectra (CDCl_3_) of compound **13b**.

**Figure S52 -** ^1^H and ^13^C NMR spectra (CDCl_3_) of compound **12c**.

**Figure S53 -** ^1^H and ^13^C NMR spectra (CDCl_3_) of compound **13c**.

**Figure S54 -** ^1^H and ^13^C NMR spectra (CDCl_3_) of compound **12d**.

**Figure S55 -** ^1^H and ^13^C NMR spectra (CDCl_3_) of compound **13d**.

**Figure S56 -** ^1^H and ^13^C NMR spectra (CDCl_3_) of compound **12e**.

**Figure S57 -** ^19^F NMR spectrum (CDCl_3_) of compound **12e**.

**Figure S58 -** ^1^H and ^13^C NMR spectra (CDCl_3_) of compound **13e**.

**Figure S59 -** ^19^F NMR spectrum (CDCl_3_) of compound **13e**.

**Figure S60 -** ^1^H and ^13^C NMR spectra (CDCl_3_) of compound **12f**.

**Figure S61 -** ^19^F NMR spectrum (CDCl_3_) of compound **12f**.

**Figure S62 -** ^1^H and ^13^C NMR spectra (CDCl_3_) of compound **13f**.

**Figure S63 -** ^19^F NMR spectrum (CDCl_3_) of compound **13f**.

**Figure S64 -** ^1^H and ^13^C NMR spectra (CDCl_3_) of mixture **12g:13g**.

**Figure S65 -** ^1^H and ^13^C NMR spectra (CDCl_3_) of compound **12h**.

**Figure S66 -** ^1^H and ^13^C NMR spectra (CDCl_3_) of compound **13h**.

**Figure S67 -** ^1^H and ^13^C NMR spectra (CDCl_3_) of compound **12i**.

**Figure S68 -** ^1^H and ^13^C NMR spectra (CDCl_3_) of compound **13i**.

**Figure S69 -** ^1^H NMR spectrum (CDCl_3_) of mixture **12j:13j**.

**Figure S70 -** ^1^H and ^13^C NMR spectra (CDCl_3_) of compound **14a**.

**Figure S71 -** ^19^F NMR spectrum (CDCl_3_) of compound **14a**.

**Figure S72 -** ^1^H and ^13^C NMR spectra (CDCl_3_) of compound **15a**.

**Figure S73 -** ^19^F NMR spectrum (CDCl_3_) of compound **15a**.

**Figure S74 -** ^1^H and ^13^C NMR spectra (CDCl_3_) of compound **14b**.

**Figure S75 -** ^19^F NMR spectrum (CDCl_3_) of compound **14b**.

**Figure S76 -** ^1^H and ^13^C NMR spectra (CDCl_3_) of compound **15b**.

**Figure S77 -** ^19^F NMR spectrum (CDCl_3_) of compound **15b**.

**Figure S78 -** ^1^H and ^13^C NMR spectra (CDCl_3_) of compound **14c**.

**Figure S79 -** ^19^F NMR spectrum (CDCl_3_) of compound **14c**.

**Figure S80 -** ^1^H and ^13^C NMR spectra (CDCl_3_) of compound **15c**.

**Figure S81 -** ^19^F NMR spectrum (CDCl_3_) of compound **15c**.

**Figure S82 -** ^1^H and ^13^C NMR spectra (CDCl_3_) of compound **14d**.

**Figure S83 -** ^1^H and ^13^C NMR spectra (CDCl_3_) of compound **15d**.

**Figure S84 -** ^1^H and ^13^C NMR spectra (CDCl_3_) of compound **14e**.

**Figure S85-** ^1^H and ^13^C NMR spectra (CDCl_3_) of compound **15e**.

**Figure S86 -** ^1^H and ^13^C NMR spectra (CDCl_3_) of compound **14f**.

**Figure S87 -** ^1^H and ^13^C NMR spectra (CDCl_3_) of compound **15f**.

**Figure S88 -** ^1^H and ^13^C NMR spectra (CDCl_3_) of compound **14g**.

**Figure S89 -** ^1^H and ^13^C NMR spectra (CDCl_3_) of compound **15g**.

**Figure S90 -** ^1^H and ^13^C NMR spectra (CDCl_3_) of compound **14h**.

**Figure S91 -** ^1^H and ^13^C NMR spectra (CDCl_3_) of compound **15h**.

**Figure S92 -** ^1^H and ^13^C NMR spectra (CDCl_3_) of compound **14i**.

**Figure S93 -** ^1^H and ^13^C NMR spectra (CDCl_3_) of compound **15i**.

**Preliminary screen of *in vitro* activity of compounds against the hepatic stage of *P. berghei* infection**


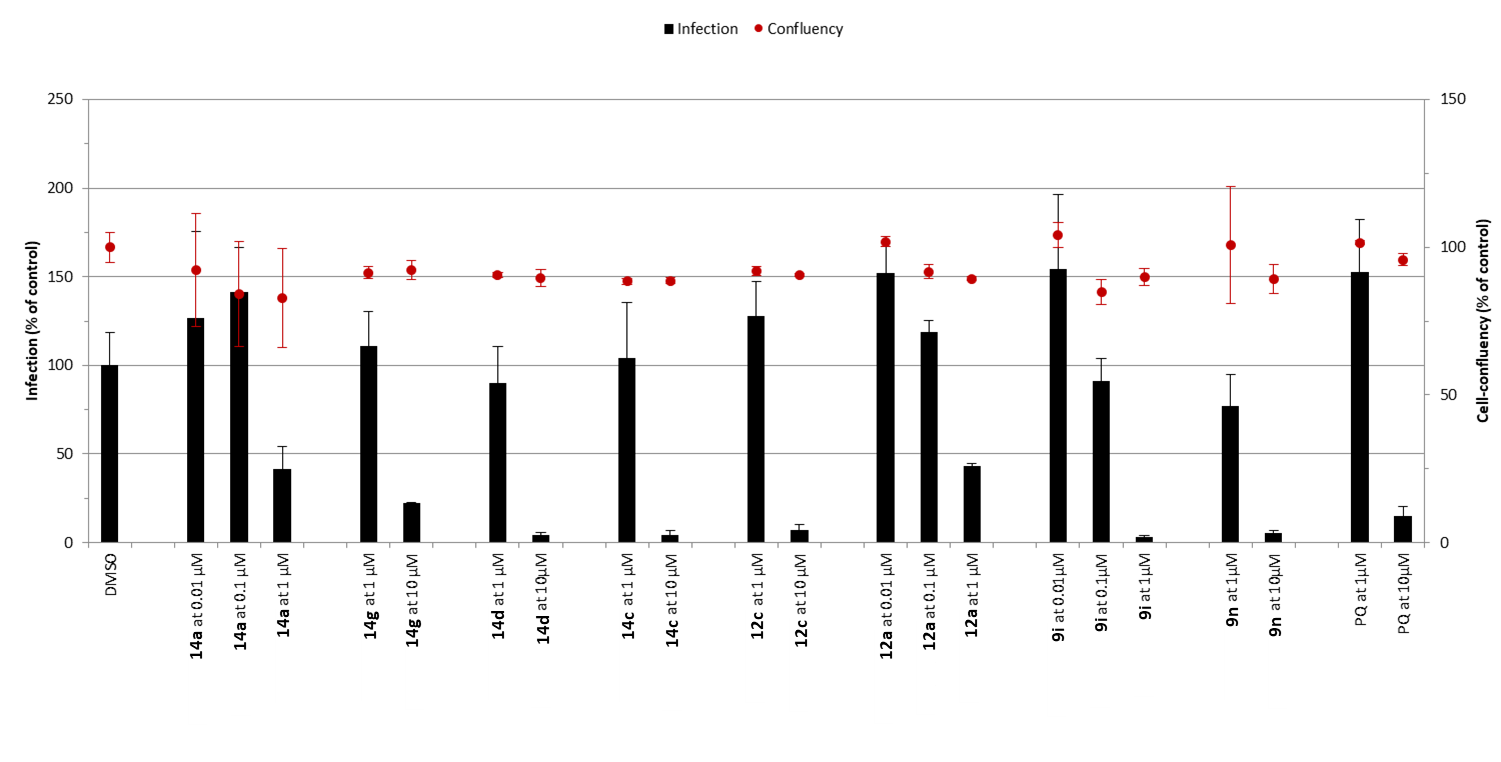


**Figure S94 -** Assessment of *in vitro* activity of compounds against the hepatic stage of *P. berghei* infection.

**qHNMR of selected compounds**

**Figure S95 –** qHNMR spectrum (CDCl_3_) of compound **9i**

##TITLE= Parameter file, TopSpin 3.6.4

##JCAMPDX= 5.0

##DATATYPE= Parameter Values

##NPOINTS= 8 $$ modification sequence number

##ORIGIN= Bruker BioSpin GmbH

##OWNER= nmrsu

$$ 2022-06-23 16:29:33.394 +0100 nmrsu@CZC1249YB0

$$ /nmrccc/service/data/asa/nmr/NGA336A/2/pdata/1/proc

$$ process /opt/topspin3.6.4/prog/mod/dataserver

##$ABSG= 5

##$DC= 2

##$FCOR= 0.5

##$FTSIZE= 65536

##$GB= 0.05

##$LB= 0.1

##$SI= 262144

m**_9i_** = 9.60 mg

MW**_9i_** = 687.80 g/mol

n**_9i_** = 1

Int**_9i_** ^a^ = 1.05

^a^ The integral of the target compound was calculated as the average of signals at 3.10, 3.53, 4.52, 5.16, 5.42 and 6.91 ppm.

The Internal Calibrant (IC) was 1,3,5-Trimethoxybenzene

M**_IC_** = 4.2 mg

MW**_IC_** = 168.19 g/mol

n**_IC_** = 3

Int**_IC_** = 5.82

Purity of **9i** = 96.8%

**Figure S96 –** qHNMR spectrum (CDCl_3_) of compound **12a**

##TITLE= Parameter file, TopSpin 3.6.4

##JCAMPDX= 5.0

##DATATYPE= Parameter Values

##NPOINTS= 14 $$ modification sequence number

##ORIGIN= Bruker BioSpin GmbH

##OWNER= nmrsu

$$ 2022-06-23 17:25:09.197 +0100 nmrsu@CZC1249YB0

$$ /nmrccc/service/data/asa/nmr/ASA550A/2/pdata/1/proc

$$ process /opt/topspin3.6.4/prog/au/bin/proc_1d

##$ABSG= 5

##$DC= 2

##$FCOR= 0.5

##$FTSIZE= 65536

##$GB= 0.05

##$LB= 0.1

##$SI= 262144

M**_12a_** = 9.70 mg

MW**_12a_** = 675.76 g/mol

n**_12a_** = 1

Int**_12a_** ^a^ = 1.05

^a^ The integral of the target compound was calculated as the average of signals at 3.11, 3.53, 4.52, 5.16, 5.41, 6.91 and 7.08 ppm.

The Internal Calibrant (IC) was 1,3,5-Trimethoxybenzene

M**_IC_** = 4.0 mg

MW**_IC_** = 168.19 g/mol

n**_IC_** = 3

Int**_IC_** = 5.35

Purity of **12a** = 97.6%

**Figure S97 –** qHNMR spectrum (CDCl_3_) of compound **14a**

##TITLE= Parameter file, TopSpin 3.6.4

##JCAMPDX= 5.0

##DATATYPE= Parameter Values

##NPOINTS= 11 $$ modification sequence number

##ORIGIN= Bruker BioSpin GmbH

##OWNER= nmrsu

$$ 2022-06-24 10:41:39.250 +0100 nmrsu@CZC1249YB0

$$ /nmrccc/service/data/asa/nmr/ASA-733A/2/pdata/1/proc

$$ process /opt/topspin3.6.4/prog/au/bin/proc_1d

##$ABSG= 5

##$DC= 2

##$FCOR= 0.5

##$FTSIZE= 65536

##$GB= 0.05

##$LB= 0.1

##$SI= 262144

m**_14a_** = 9.60 mg

MW**_14a_** = 705.79 g/mol

n**_14a_** = 1

Int**_14a_** ^a^ = 1.05

^a^ The integral of the target compound was calculated as the average of signals at 3.10, 3.51, 4.51, 5.08, 5.40 and 6.91 ppm.

The Internal Calibrant (IC) was 1,3,5-Trimethoxybenzene

M**_IC_** = 4.00 mg

MW**_IC_** = 168.19 g/mol

n**_IC_** = 3

Int**_IC_** = 5.66

Purity of **14a** = 97.3%
